# Exonic splicing code and coordination of divalent metals in proteins

**DOI:** 10.1093/nar/gkad1161

**Published:** 2023-12-06

**Authors:** Dara Bakhtiar, Katarina Vondraskova, Reuben J Pengelly, Martin Chivers, Jana Kralovicova, Igor Vorechovsky

**Affiliations:** University of Southampton, Faculty of Medicine, Southampton SO16 6YD, UK; Slovak Academy of Sciences, Centre of Biosciences, 840 05 Bratislava, Slovak Republic; University of Southampton, Faculty of Medicine, Southampton SO16 6YD, UK; University of Southampton, Faculty of Medicine, Southampton SO16 6YD, UK; University of Southampton, Faculty of Medicine, Southampton SO16 6YD, UK; Slovak Academy of Sciences, Centre of Biosciences, 840 05 Bratislava, Slovak Republic; University of Southampton, Faculty of Medicine, Southampton SO16 6YD, UK

## Abstract

Exonic sequences contain both protein-coding and RNA splicing information but the interplay of the protein and splicing code is complex and poorly understood. Here, we have studied traditional and auxiliary splicing codes of human exons that encode residues coordinating two essential divalent metals at the opposite ends of the Irving–Williams series, a universal order of relative stabilities of metal–organic complexes. We show that exons encoding Zn^2+^-coordinating amino acids are supported much less by the auxiliary splicing motifs than exons coordinating Ca^2+^. The handicap of the former is compensated by stronger splice sites and uridine-richer polypyrimidine tracts, except for position –3 relative to 3′ splice junctions. However, both Ca^2+^ and Zn^2+^ exons exhibit close-to-constitutive splicing in multiple tissues, consistent with their critical importance for metalloprotein function and a relatively small fraction of expendable, alternatively spliced exons. These results indicate that constraints imposed by metal coordination spheres on RNA splicing have been efficiently overcome by the plasticity of exon–intron architecture to ensure adequate metalloprotein expression.

## Graphical Abstract

**Graphical Abstract:**
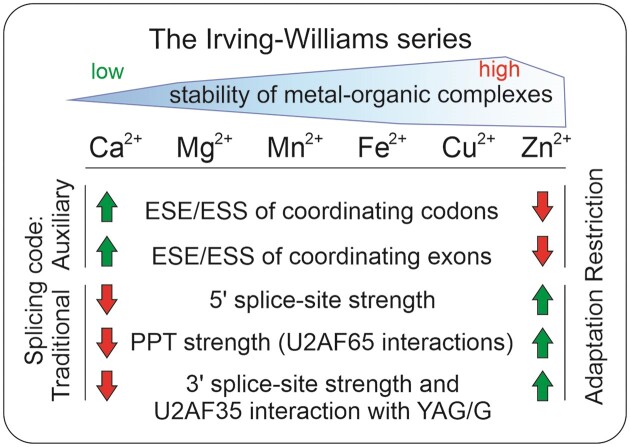

## Introduction

Metal-binding complements of eukaryotic proteomes, or metalloproteins, require metal ions to assist catalysis or impart structure (1). Metalloproteins account for a large proportion of gene products in many organisms (2–5). For example, the number of human proteins predicted to bind Ca^2+^, Mg^2+^ or Zn^2+^, the most abundant divalent ions in the human body, was estimated at ∼10^5^ each (6), while almost half of the enzymes structurally characterized in the Protein Data Bank (PDB) need metals (1,4). Because proteins are flexible, steric selection of metals is imperfect, especially by nascent polypeptides first emerging from the ribosome (7). Under such conditions the relative affinities of divalent metals for proteins have a tendency to follow ligand field stabilization energies of metals themselves, creating a universal order of binding preferences known as the Irving–Williams series (7–9), first proposed for a subset of transition metals 75 years ago (10). For essential divalent metals abundant in humans [ppm > 50 (9)], this order is Ca^2+^∼Mg^2+^<Mn^2+^ <Fe^2+^<Cu^2+^≥Zn^2+^, with Ca^2+^ and Mg^2+^ forming the weakest and Cu^2+^ and Zn^2+^ forming the tightest complexes with organic ligands (1,5,7,8,11). In equimolar mixtures of these and other divalent metals in the series (Co^2+^ and Ni^2+^), proteins that require weaker binding ions preferentially bind tighter metals. Although metalloproteins can bind incorrect metals *in vitro*, metalation *in vivo* is usually accurate (12,13), implying that cells evolved efficient strategies to overcome or reduce these constraints and avoid binding by tight non-cognate metals (mismetalation) to safeguard selective metal-binding properties of their polymers (7,14,15). Harmful mismetalation events can distort geometry of cognate metal-binding sites, recruit undesired ligands or exploit only a subset of native ligands (1). Hence, it is essential to understand how protein-coding genes and their products manage metal acquisition to avoid mismetalation. However, the underlying mechanisms and relative contributions of eukaryotic gene expression steps to strategies counteracting mismetalation and metal coordination constraints are poorly understood.

Eukaryotic gene expression involves a series of highly coordinated steps, starting from transcription and splicing of mRNA precursors (pre-mRNAs), followed by mRNA export, translation and decay (16). These steps require multi-component cellular machines that are tethered to each other (16). In humans, the most complex step is believed to be pre-mRNA splicing, which removes intervening sequences or introns and joins coding sequences or exons together with single-nucleotide precision. This process is orchestrated by the spliceosome, a 2.7 MDa RNA–protein complex consisting of small nuclear RNAs U1, U2 and U4–U6, and ∼125 associated proteins in spliceosomal assembly intermediates (17). The spliceosome forms *ad hoc* on each intron to recognize 3′ and 5′ splice sites (3′ss and 5′ss), lariat branchpoints and polypyrimidine tracts (PPTs) (17). Although conserved, these traditional or core signals are insufficient for accurate splicing, which requires additional pre-mRNA motifs in exons and introns, particularly in higher vertebrates and plants (18). These auxiliary motifs have become known as exonic or intronic splicing enhancers or silencers (ESEs/ESSs and ISEs/ISSs) (19–23). Because most exons are protein coding, and ESEs/ESSs and amino acids are encoded by the same nucleotides, ESE/ESS evolution had to be shaped by both splicing and protein-coding restrictions (24,25). Indeed, a large proportion of the splicing information in ESEs, recently estimated at ∼50%, is coincident with the protein-coding information (26) and can be characteristic of a protein domain (27). Many mutations that disrupt protein function were shown to alter pre-mRNA splicing (24). Although the exact relationship of the two selective forces remains obscure, the high number of metal-binding residues in the human proteome provides an opportunity to better understand the impact of metal coordination restrictions on the auxiliary splicing code in exons.

**Figure 1.**
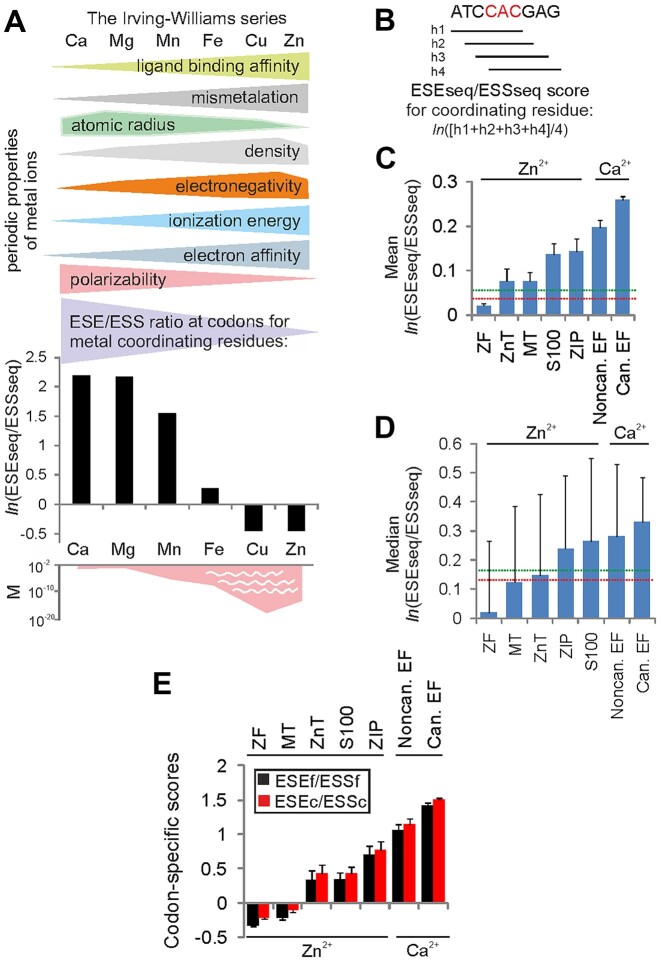
ESE/ESS profiles of codons for Zn^2+^- and Ca^2+^-coordinating residues in human proteins. (**A**) The Irving–Williams series and the auxiliary splicing code in exons: a hypothesis. The upper panel shows selected properties of six abundant and biologically important divalent metals in the human body and their non-linear trends in the Periodic Table of Elements. Atomic radius, the distance from atomic nucleus to outermost electron orbital; electronegativity, a tendency of an atom in a molecule to attract shared electrons; ionization energy, the amount of energy required to remove the first electron from neutral atoms; electron affinity, the energy released when an electron is added; polarizability, a tendency to acquire electric dipole moment in electric fields (133). The middle panel shows ESE/ESS profiles for codons encoding coordinating residues for the indicated metal [adapted from (28)]. The ESEseq/ESSseq values were weighted by amino acid frequencies at metal-binding sites estimated by fragment transformation methods (89). The bottom panel shows estimates of metal concentrations (mol/l) in ancient (sulfidic and anoxic) sea waters (>4000 million years ago). Concentrations were compiled from (11,134,135). (**B**) Average ESEseq/ESSseq score calculations across codons for Zn^2+^- and Ca^2+^-binding sites (red). ESEseq and ESSseq scores (22) for overlapping hexamers (h, horizontal bars) were averaged for each coordinating codon as indicated. Splicing-neutral hexamers (22) were ignored. (**C** and **D**) Average (**C**) and median (**D**) values of ESEseq/ESSseq ratios across metal-coordinating codons in major groups of Zn^2+^- and Ca^2+^-BPs: ZF, zinc fingers; ZnT, Zn^2+^ transporters; MT, metallothioneins; ZIP, Zrt/Irt-like proteins; EF, non-canonical and canonical EF-hand proteins. Error bars for means (**C**) are standard errors of the mean (SEMs), error bars for medians (**D**) are interquartile ranges. Horizontal dotted lines denote values for various control exon groups, with a maximum value denoted in green and minimum in red. Significant differences between groups were seen with both parametric [single-factor analysis of variance (ANOVA) followed by the Tukey–Kramer post-hoc test, *F*-value = 116.2, *P*< 10^−16^] and non-parametric (Kruskal–Wallis one-way ANOVA, H statistics = 651.3, *P*< 0.00001) tests. (**E**) Codon-specific ESE/ESS scores of the same protein groups. Columns denote means, error bars are SEMs.

The ESE/ESS profiles have been recently linked to amino acid frequencies at binding sites for divalent metals (28). The weakest binders of the Irving–Williams series (Ca^2+^, Mg^2+^) were shown to preferentially bind residues encoded by splice-enhancing codons, whereas the tight binders (Cu^2+^, Zn^2+^) are coordinated to amino acids encoded by splice-repressing codons, with moderate binders in the series (Mn^2+^, Fe^2+^) exhibiting intermediate codon-specific ESE/ESS values (28) (Figure 1A). This splicing dichotomy suggested that protein-binding sites for weak divalent metals may have been promoted during evolution at the exon level, whereas sites for competitive metals may have been repressed, potentially reducing mismetalation. Notably, codons for residues that coordinate calcium in Ca^2+^-binding proteins (CaBPs) have the capacity to promote exon inclusion in mature transcripts significantly more than the average (27). The high potential of codons for Ca^2+^-coordinating residues to be retained in mature transcripts may have facilitated the expansion of Ca^2+^-binding sites such as EF-hands during evolution (27). However, auxiliary splicing motifs in bona fide exons have not been studied for other metalloproteins, and it remains unclear if they have been influenced by constraints of the Irving–Williams series and periodic trends of divalent metal properties (Figure 1A).

In this study, we have characterized ESE/ESS profiles for sequences that encode protein-binding sites for Zn^2+^, a tight binder in the Irving–Williams series (1,7,8,11). As predicted (28), we demonstrate that codons for Zn^2+^-coordinating residues in human proteins confer a lower capacity to retain these coding sequences in mature transcripts than codons that coordinate weak Ca^2+^. We also show that entire exons encoding Zn^2+^-coordinating residues have significantly lower ESE/ESS ratios than exons encoding Ca^2+^-coordinating residues. However, both groups of exons show a relative scarcity of alternative splicing as compared with average exons, which was supported by RNA sequencing (RNA-seq) and expressed sequence tag (EST) datasets, and by polymerase chain reaction (PCR)-based validation of exon candidates for regulated splicing. We also demonstrate that the diminished inclusion capacity of Zn^2+^-coordinating codons and their exons to be included in mRNAs was compensated by the increased intrinsic strength of their splice sites, PPTs and the remaining ESE/ESS within their exons. In this respect, we have explored the role of pyrimidine transitions at position –3 relative to 3′ss, and provide evidence for their splicing preferences, supporting distinct interactions between each subunit of the auxiliary factor of U2 (U2AF) and exons encoding Zn^2+^- versus Ca^2+^-binding residues. Together, these results show how traditional and auxiliary splicing motifs in the pre-mRNA evolved to alleviate constraints imposed by coordination of essential divalent metals, providing new insights into the interplay of protein and splicing codes in human DNA.

## Materials and Methods

### Extraction and validation of exons that code for Zn^2+^-binding sites in proteins

A non-redundant set of DNA sequences encoding Zn^2+^-coordinating residues was compiled using the following resources: (i) PDB; (ii) UniprotID (29); (iii) ZincBind (30), (iv) the Database of Metal Binding Sites (31); and (v) GeneCards (https://www.genecards.org). We prepared two main datasets, termed stringent and extended (Table 1; Supplementary Datasets S1–S4). The stringent dataset contained a total of 3862 Zn^2+^-coordinating residues in ∼990 Zn^2+^-binding sites in 437 mammalian proteins with structural evidence for Zn^2+^ binding, as determined by nuclear magnetic resonance (NMR) spectroscopy or X-ray crystallography (average resolution 2.3 Å). The dataset also included structures investigated by electron microscopy (SLC30A8, USP39, RPL37, COX5B, POLR3K, POLR2I, RPS29, POLR2L, POLR2B, RPAP2, POLR3B, FBXO5 and POLR2K). Most structures were derived from human proteins, with just ∼19% from non-human mammals (Supplementary Table S1). To expand the sample size further while capitalizing on a high conservation of ESE/ESS motifs in higher vertebrates (18), we also compiled the extended dataset, which contained residues predicted to bind Zn^2+^ that were inferred from multiple sequence alignments of conserved groups of Zn^2+^-binding proteins (ZnBPs) (Table 1; Supplementary Figures S1–S4). In this dataset, amino acids were considered Zn^2+^ coordinating if their alignment positions in other species shared identical residues with human proteins. If the alignment showed distinct residues at this position, we included only residues that were previously shown to coordinate Zn^2+^, as exemplified by substitutions: H113 > E and H115 > S in KLK2; H183 > Q in NLN; D20 > G in OMP; or H231 > S or E236 > L in KLK3. Other residues at shared alignment positions were not considered in subsequent analyses, such as D20 > G in OMP or E236 > L in KLK3. As compared with the stringent dataset, the extended dataset contained sequences encompassing additional 603 Zn^2+^-coordinating amino acids (Table 1).

**Table 1.** Datasets of codons that encode Zn^2+^-coordinating residues

Supplementary Online Dataset	Extended through homology in multiple alignments	CMM filtered	No. of codons^a^	No. of proteins
S1 (stringent, without CMM filter)	No	No	3862	437
S2 (stringent, CMM filtered)	No	Yes	3348	395
S3 (extended, without CMM filter)	Yes	No	4465	482
S4 (extended, CMM filtered)	Yes	Yes	3951	440

^a^Residues annotated in PDB as Zn^2+^ binding but not metal coordinating were not included.

**Table 2.** ZnBPs included in the study

ZnBP group	No. of proteins	No. of Zn^2+^-coordinating codons	Zn^2+^-coordinating codons in the vicinity of splice sites^a^
ZnTs	10	142	3
ZIPs	14	128	4
MTs	11	220	11
S100	20	193	0
ZFs and other ZnBPs	427	3782	149
Total	482	4465	167

^a^< 3 nt from exon–intron junctions.

Because metal-containing structures in PDB may contain incorrect metal assignments (32), PDB identifiers with evidence for Zn^2+^ binding were subjected to validation. We employed CheckMyMetal (CMM) (33), a sophisticated algorithm, to categorize Zn^2+^-binding sites into three groups (termed plausible, problematic and not validated; coloured in green, orange and red in Supplementary Datasets S1–S4). This classification was based on six validation parameters, including valence, geometry, ligand and vacancy (32). The CMM validation was carried out for both stringent and extended datasets to create filtered datasets, each without low resolution CMM adjustments (Table 1). Residues not validated as metal-binding sites by CMM for at least two validation parameters were excluded from ESE/ESS profiling (Table 1).

Finally, amino acids included in the four datasets and their flanking sequences were matched to human coding sequences in Ensembl (http://www.ensembl.org, build 104) to obtain non-redundant Zn^2+^-coordinating codons and exons for ESE/ESS profiling, totalling 482 different ZnBPs (Supplementary Datasets S1–S4).

### Characterization of ESEs/ESSs that encode Zn^2+^-coordinating residues in proteins

Spliceosomes of higher vertebrates recognize small exons in the sea of very long introns in a process known as exon definition (34). Their choice to include (exons) or exclude (introns) a pre-mRNA sequence in or from mature transcripts is strongly influenced by the balance of ESEs and ESSs, which exhibit a gradient in exon–intron definition on a continuous scale of exon inclusion capacity (19,20,22,35). To characterize the auxiliary splicing code underlying Zn^2+^-binding sites in proteins, we employed a comprehensive set of ESE and ESS hexamers that were previously derived by *ex vivo* splicing promotion or repression afforded by 4096 synthetic oligomers inserted into two model exons at five different positions (22). The resulting duplicate minigene libraries were used to obtain hexamer ESEseq and ESSseq scores, which were calculated independently of metal binding affinities and which provide good estimates of exon inclusion activities (22).

To assign ESEseq and ESSseq scores to codons for residues coordinating Zn^2+^, we computed average scores of four overlapping hexamers using custom Microsoft Excel functions/formulas (Figure 1B; Supplementary Figure S5). We applied the same procedure to each of the four datasets and also to codons encoding Ca^2+^-coordinating residues, which were ascertained previously (27). Apart from ESEseq/ESSseq scores, we calculated frequency ratios for a total of 4728 ESE codons and 4360 ESS codons, shown here as ln(ESEf/ESSf) (Supplementary Dataset S5). We also determined codon counts in 1182 high-confidence ESEs and 1090 high-confidence ESSs (22) to compute the ESEc/ESSc ratios. The two measures estimate codon-specific splicing activities, perhaps barring stop codons since these translation termination signals may induce capricious nonsense-mediated mRNA decay in transient transfections of plasmid DNA (36), although this was considered unlikely (22). For control datasets, we extracted RefSeq sequences of human protein-coding exons as defined by the UCSC Table Browser (https://genome.ucsc.edu/cgi-bin/hgTables), comprising ∼35 million hexamers in ∼200 000 exonic segments, as described (27). Mean ESEseq/ESSseq, ESEf/ESSf and ESEc/ESSc values were computed for exons devoid of the first and the last three nucleotides (nt) since these exonic positions shape the 3′ss and 5′ss consensus, respectively.

### mRNA inclusion levels of exons that encode Zn^2+^- and Ca^2+^-coordinating residues

To determine PSI (percent spliced in) values (37) for our tested exons, we employed PSI tables (hg38) from the Vertebrate Alternative Splicing and Transcription Database (VastDB) (38). VastDB provides a comprehensive PSI resource across vertebrate exons in various tissues and developmental stages (38). In addition, we compared PSI values of tested and control exons using EST data in HEXEvent, a database of Human EXon splicing Events, which stores EST-derived inclusion levels for ∼200 000 exons (39). HEXEvent shows alternative splicing information for human internal exons but, unlike VastDB, HEXEvent does not include intron retention events, avoiding a bias toward short retained introns (25,39).

### Comparisons of traditional splicing signals

To determine the intrinsic strength of exon splice sites, we used maximum entropy scores defined previously (40), which successfully predict aberrant 3′ss and 5′ss induced by human pathogenic mutations (41,42). To compare the strength and location of lariat branchpoints of tested exons in ZnBPs and CaBPs, we employed BPP (43) and SVM-BPfinder (44), currently the best performing algorithms for *ab initio* branchpoint prediction that consider PPTs and AG dinucleotide exclusion zones (43,45). We ignored branchpoints outside the range between –13 and –60 nt relative to the 3′ss, which contains the vast majority of human branchpoints (44,46,47) but not distant branchpoints, which are rare (28,48). We used WebLogo (49) to display relative nucleotide frequencies of predicted branchpoints with the highest branchpoint scores in the two groups of exons and in controls. Location of branchpoint motifs in intronic sequence is shown in Supplementary Dataset S6. The dataset also compares BPP and SVM-BPfinder assignments with 59 359 high-confidence branchpoints identified by Mercer *et al.* (47) and identifies non-canonical introns, including AT–AC introns, whose branchpoint motifs may be recognized by U12 (50), and an AT–AA intron (51). The fraction of U12-type introns (52,53) was 0.2% in ZnBP exons and 3.5% in CaBP exons (Supplementary Dataset S6). Control introns were obtained from the UCSC browser using curated mRNA transcripts from NCBI RefSeq, totalling 195 903 unique entries (hg38).

### Validation of alternative splicing of exons encoding Zn^2+^- and Ca^2+^-binding residues

Selection of candidate exons for experimental validation and regulated splicing was based on average VastDB PSI values lower than 90% in neural or muscle tissue, testis or embryonal stem cells. In addition, total RNA was prepared from exponentially growing cell lines HEK293 (54) and SH-SY5Y [ATCC CRL-2266 (55)] that were derived from human embryonal kidney and human neuroblastoma, respectively. In addition, we used total RNA isolated from 16 human tissues, including brain and skeletal muscles (Ambion, cat. # 0912010 and 0905009). Total RNA samples were reverse transcribed with the Moloney murine leukaemia virus reverse transcriptase (SuperScript III RT, Invitrogen) and oligo d(T) primers according to the manufacturer's recommendation. Reverse transcription–PCR (RT–PCR) primers (Supplementary Table S2) were designed to target exons adjacent to tested exons that had average PSI values below the indicated limit. PCRs were carried out with DreamTaq Green PCR Master Mix (Thermo Scientific™) with 1.5 mM Mg^2+^ and at two annealing temperatures. PCR products were separated on 1.5% agarose gels. Signal intensities of the spliced products were measured using the Amersham Imager 600 (GE Healthcare) to estimate exon inclusion levels.

### *Ex vivo* splicing assays

Splicing reporters containing pyrimidine transitions at position –3 of 3′ss were first tested for three human genes: *F8* (56), *UBE2F* (57) and *HGD* (58). The reporters were selected from our minigene library owing to the presence of at least four contiguous pyrimidines at PPT, uridine or cytosine at position –3, and the presence of spliced products with and without the middle exon upon transient transfections. Plasmid mutagenesis was carried out using overlap extension PCR with primers shown in Supplementary Table S3 to obtain constructs with weaker and stronger PPTs and weaker and stronger exon positions +1 through +3 in the presence of CAG or TAG 3′ss. Mutated constructs were Sanger-sequenced to exclude undesired mutations (Eurofins). The minigene collection was then extended to include 19 additional constructs to test disease-associated single-nucleotide substitutions at position –3 reported in the literature and 24 additional reporters with mid-exons encoding coordinating residues for Zn^2+^ or Ca^2+^ (Supplementary Tables S4 and S5).

Plasmid DNA samples were transiently transfected into HEK293 or Chinese hamster ovary (CHO) cells. HEK293 cells were grown in Dulbecco’s modified Eagle’s medium (DMEM) supplemented with 10% (v/v) bovine calf serum (Biosera) under standard conditions. CHO cells were grown in Ham’s F12 supplemented with 2 mM glutamine and 10% bovine calf serum. Transfections were carried out in 12- or 24-well plates using 150 ng of reporter DNA and jetPRIME (Polyplus) according to the manufacturer's recommendations. The cells were lysed 24 h later for RNA extraction. Total RNA was isolated using TRI Reagent (Molecular Research Center) according to the manufacturer's protocol and used for the first-strand cDNA synthesis with the Moloney murine leukaemia virus reverse transcriptase (Promega) and oligo d(T) primers. RT–PCRs were carried out using vector-specific primers (59) to obtain spliced products from exogenous transcripts. PCR products were separated on agarose gels and the amount of exon inclusion and skipping was measured with the Amersham Imager 600 (GE Healthcare).

## Results

### Auxiliary splicing code in exons and protein-binding sites for Zn^2+^

To test the hypothesis shown in Figure 1A and to determine the extent to which codons that encode Zn^2+^-coordinating residues support exon inclusion in mature transcripts, we extracted and validated their nucleotide sequences from human proteomic, structure and genomic databases (see the Materials and Methods). To compare codons for tight (Zn^2+^) and weak (Ca^2+^) metal-binding sites using the same method (Figure 1B), we assigned hexamer ESEseq and ESSseq scores (22) to codons encoding Zn^2+^-coordinating residues (Supplementary Datasets S1–S4) and codons for Ca^2+^-coordinating residues obtained previously (27). Figure 1C and D shows that codons for Zn^2+^-coordinating residues have, in aggregate, a significantly lower ESEseq/ESSseq ratio than codons encoding Ca^2+^-coordinating residues, here exemplified by EF-hand proteins. The most reduced values were found for zinc fingers (ZFs), a copious group of human ZnBPs in which Zn^2+^ largely binds Cys or His residues (60). In contrast, ZnBPs where Zn^2+^ is increasingly coordinated by acidic amino acids showed higher predicted exon inclusion levels (Figure 1C, D). These ZnBPs included Zn^2+^ transporter proteins, which move Zn^2+^ out of (ZnTs) and into (ZIPs, Zrt/Irt-like proteins) the cell, acting in opposite directions and selectively binding Zn^2+^ to stabilize its cytosolic concentration (61,62), metallothioneins (MTs) and S100 proteins, which often bind divalent metals other than Zn^2+^, including Ca^2+^ (63) (Table 2). The exonization potential of Zn^2+^-coordinating codons in ZnBPs showed a hierarchy of ZIPs/S100 > ZnTs/MTs > ZFs (Figure 1C, D). This hierarchy did not change when removing Zn^2+^-coordinating codons located at the start or end of exons, which constituted ∼3.5% of the total sample size. Their replacement with extended native motifs yielded very similar values. As compared with the extended dataset, the stringent dataset devoid of ZIPs and many ZnTs, MTs and S100 proteins showed only a small reduction in mean and median values, consistent with a higher fraction of core Zn^2+^-coordinating residues (Cys and His) in this sample. Codon-specific scores computed for the same datasets confirmed the same hierarchy of ZnBPs and CaBPs (Figure 1E). Again, replacement of terminal triplets with extended native motifs or their removal did not significantly alter these values, or the order of the indicated protein groups.

Finally, to investigate the extent to which potentially incorrect assignment in a proportion of metal-containing structures in PDB can influence the hierarchy of ESE/ESS profiling, we removed PDB structures that could not be validated by CMM (32) from our datasets (Table 1). However, the hierarchy of the five groups of ZnBPs remained the same in the CMM filtered data, each group showing significantly lower ESEseq/ESSseq ratios than EF-hand proteins.

We conclude that codons coding for amino acids coordinating the two divalent metals in human proteins have the capacity to confer a significantly lower exon inclusion for tight Zn^2+^ than for weak Ca^2+^.

### mRNA inclusion of exons that encode Ca^2+^- and Zn^2+^-coordinating residues

**Figure 2.**
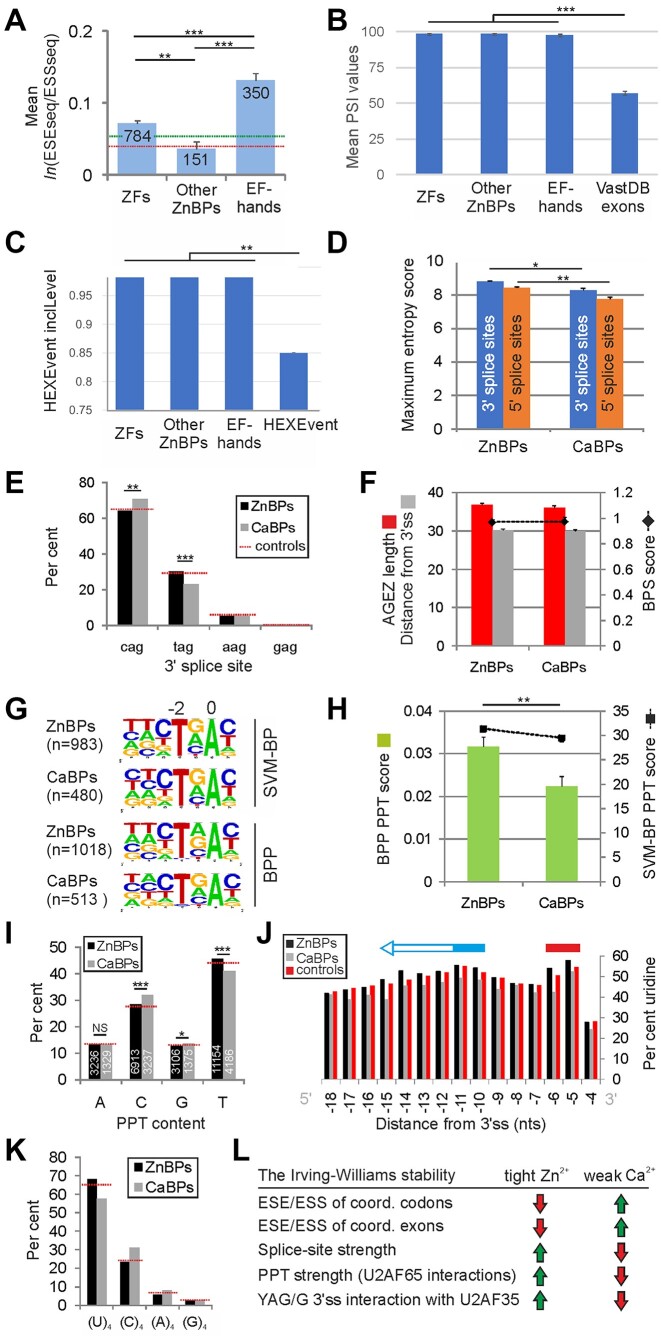
High inclusion of weak Zn^2+^ exons in mature transcripts is supported by both traditional and auxiliary splicing signals.(**A**) Mean ln(ESEseq/ESSseq) values for full exons that encode Zn^2+^- or Ca^2+^-binding sites in ZFs, other ZnBPs and canonical EF-hands. Significant differences between groups were confirmed by ANOVA (*F*-value 44.3, *P*< 0.0001). The number of exons in each group is shown at the top of each column. Error bars are SEMs. **P< 0.001, ***P< 0.0001. Horizontal dotted lines show the range for various control exon groups, demarcated by maximum (green) and minimum (red) values. (**B**) Mean PSI values in the same groups of exons. Human VastDB exons were used as controls. Error bars are SEMs. (**C**) Average EST-derived exon inclusion levels in the same groups. InclLevel values were defined previously (39). HEXEvent controls show mean inclLevel values for 227 179 human exons in the HEXEvent database. Error bars are SEMs. Additional EST-derived exon inclusion events are shown in Supplementary Figure S7. (**D**) Zn^2+^ exons have stronger splice sites than Ca^2+^ exons. The intrinsic strength of splice sites was determined by averaging maximum entropy scores (40). Error bars are SEMs. *P= 0.05, **P< 0.001 (Mann–Whitney U-test). (**E**) Frequency of (C/T/A/G)AG 3′ss in ZnBPs and CaBPs. χ^2^ for the 2 × 4 table = 12.4. **P< 0.001, ***P< 0.0001. (**F**) Average length of the AG dinucleotide exclusion zone and distance from 3′ss (left axis) for branchpoints with the highest SVM-BP scores (right axis) in introns that precede Zn^2+^ and Ca^2+^ exons. (**G**) Consensus branchpoint sequence for the two exon groups, as predicted by SVM-BP (44) and BPP (43). The number of introns is in parentheses. (**H**) Introns that precede Zn^2+^ exons have stronger PPTs than those preceding Ca^2+^ exons. Error bars are SEMs. **P< 0.001 (median test, both with SVM-BP and BPP). (**I**) Zn^2+^ exons are preceded by uridine-richer PPTs as compared with Ca^2+^ exons. The total number of nucleotides between the branchpoint and 3′ss is in white. ***P< 0.0001, **P< 0.001 (χ^2^ test). (**J**) Uridine content at positions –4 to –18 relative to 3′ss. Horizontal red and blue bars denote putative maxima of U2AF65 RRM1 and RRM2 interactions with the PPT. (**K**) Tetranucleotides in PPTs of introns preceding Zn^2+^ and Ca^2+^ exons (2 × 4 table χ^2^= 11.2, *P*< 0.0001). (**L**) Summary of compensatory trends in auxiliary and traditional splicing signals of Zn^2+^ and Ca^2+^ exons.

Next, we set out to test if the observed ESE/ESS dichotomy of codons for Zn^2+^- and Ca^2+^-coordinating amino acids can influence inclusion levels of entire exons. We disregarded exons >350 nt long to reduce a bias resulting from terminal or large exons where the fraction of coordinating codons was negligible. We also pooled ZnBPs other than ZFs into a single group to obtain a sufficient sample size. Figure 2A shows that exons that encode Ca^2+^-coordinating residues (termed Ca^2+^ exons) retained higher than average ESEseq/ESSseq scores as compared with those encoding Zn^2+^-coordinating residues (termed Zn^2+^ exons). Unlike the splice-inhibiting effect of codons for Zn^2+^-coordinating amino acids in ZFs (Figure 1C–E), the ESEseq/ESSseq values for entire ZF Zn^2+^ exons were not lower than the average but were somewhat elevated (Figure 2A). This suggests that the low exon inclusion capacity of codons encoding Zn^2+^-coordinating residues has been compensated by the auxiliary splicing code in the remaining portions of the same exons.

Next, we determined average PSI values for Zn^2+^ and Ca^2+^ exons and control exons by exploring existing, publicly available RNA-seq and EST data. Importantly, we found that average PSI values derived from RNA-seq experiments compiled in VastDB (38) were significantly higher for exons encoding residues that coordinate either metal than for controls (Figure 2B). The mean PSI values remained high across multiple tissues (Supplementary Figure S6). Because very small exons are generally less efficiently included in mature transcripts (25,34,64), we excluded small exons and microexons from VastDB PSI tables, but the difference in PSI values (Figure 2B) remained highly significant.

To further support these findings, we compared inclusion levels for the three exon groups and controls using independent EST data. We explored HEXEvent (39), which defines four types of EST-derived exon inclusion [defined as inclLevel, constitLevel, 3usageLevel and 5usageLevel in (39)], extending information from cassette exons to their major alternative splice sites. This comparison confirmed that for each exon inclusion category, Zn^2+^ and Ca^2+^ exons had higher average inclusion values than controls (Figure 2C; Supplementary Figure S7).

Together, these results reveal a close-to-constitutive inclusion and relative paucity of alternative splicing for exons that are critical for binding sites for the two arguably most important divalent metals in eukaryotic cell signalling.

### Balancing auxiliary and traditional splicing motifs by exons encoding Zn^2+^- and Ca^2+^-coordinating residues

Next, we set out to identify pre-mRNA features that overcome the low capacity of Zn^2+^-coordinating codons (Figure 1C–E) and Zn^2+^ exons (Figure 2A) to be spliced into mature transcripts in such a way that these exons are largely constitutive *in vivo* (Figure 2B, C). Comparison of the intrinsic strength of splice sites showed that Zn^2+^ exons in a pooled sample of all ZnBPs had, on average, significantly stronger 5′ss and 3′ss than Ca^2+^ exons (Figure 2D). We also observed an excess of CAG 3′ss in the latter group, largely at the expense of TAG 3′ss, while control introns showed intermediate values (Figure 2E). At the first exon position, Ca^2+^ exons had significantly fewer guanines and more adenines than Zn^2+^ exons (χ^2^= 16.1, *P*< 0.0001; Supplementary Figure S8).

Because the difference in the intrinsic splice site strength between Zn^2+^ and Ca^2+^ exons appeared smaller for 3′ss than for 5′ss (Figure 2D), and T/C mutations at position –3 relative to 3′ss may alter branchpoint selection (65), we tested if an increased strength of lariat branchpoints and/or PPTs can compensate the reduced ESE/ESS profiles of Zn^2+^ exons. However, introns preceding Zn^2+^ and Ca^2+^ exons differed neither in average distances between the strongest branchpoints and 3′ss, nor in the length of AG dinucleotide exclusion zones (Figure 2F) or PPTs (Supplementary Figure S9). Moreover, there was no significant departure from the branchpoint consensus (Figure 2G; Supplementary Dataset S5). In contrast, we found significantly higher PPT scores for Zn^2+^ exons as compared with their Ca^2+^ counterparts (Figure 2H), which was corroborated by a clear shift to uridine-richer PPTs in the former group (Figure 2I), consistent with the maximum entropy scoring of partial PPTs (20 nt preceding the 3′ss, Figure 2D). The excess of uridine in PPTs of Zn^2+^ exons over Ca^2+^ exons could be seen at each position between –4 and –18 nt relative to the 3′ss, with the first peak at position –5 and a second, broader peak extending toward branchpoints from position –11 in controls to position –15 upstream (blue arrow in Figure 2J).

The two uridine peaks at PPTs are thought to reflect interactions with RNA-recognition motifs RRM1 and RRM2 of the large U2AF subunit (U2AF65) (66, and references therein) and possibly with other RNA-binding proteins. We therefore compared the frequencies of (U)_4_, an optimal binding platform for U2AF65 *in vitro* and *in vivo* (67, and references therein), in the two groups of PPTs downstream of predicted branchpoints. We found that introns preceding Zn^2+^ exons had a significantly higher fraction of PPTs with at least one (U)_4_ motif than those preceding Ca^2+^ exons (47.5% versus 37.4%, χ^2^= 8.4, *P*< 0.0001), largely at the expense of (C)_4_ repeats (Figure 2K). In addition, distribution of distances between these motifs and 3′ss showed noticeable shifts at positions –6 through –15 for (U)_4_ and –6 through –25 for (C)_4_ (Supplementary Figure S10A, B). As compared with control introns, the fraction of PPTs with at least one (U)_4_ between BPP branchpoints and 3′ss was lower for Ca^2+^ exons and slightly higher for Zn^2+^ exons, whereas the opposite was observed for (C)_4_ motifs (Supplementary Figure S10C).

Finally, we also examined the overlap between Zn^2+^ and Ca^2+^ exons and those differentially expressed in cells depleted of the small subunit of U2AF (U2AF35) (54), and identified multiple genes and exons in common, exemplified by Ca^2+^-binding *SDF4* or *MCFD2* or Zn^2+^-binding *ZFAND1*. Figure 2L and the Graphical abstract summarize changes in traditional and auxiliary splicing signals for Zn^2+^ and Ca^2+^ exons and predicted U2AF interactions.

Taken together, these results suggest that core splicing signals for Zn^2+^ and Ca^2+^ exons evolved to compensate their metal-specific ESE/ESS signals. They also support distinct 3′ss interactions of the two exon groups with U2AF early during spliceosome assembly.

### Alternative splicing of exons encoding coordinating residues for Ca^2+^ and Zn^2+^

**Figure 3.**
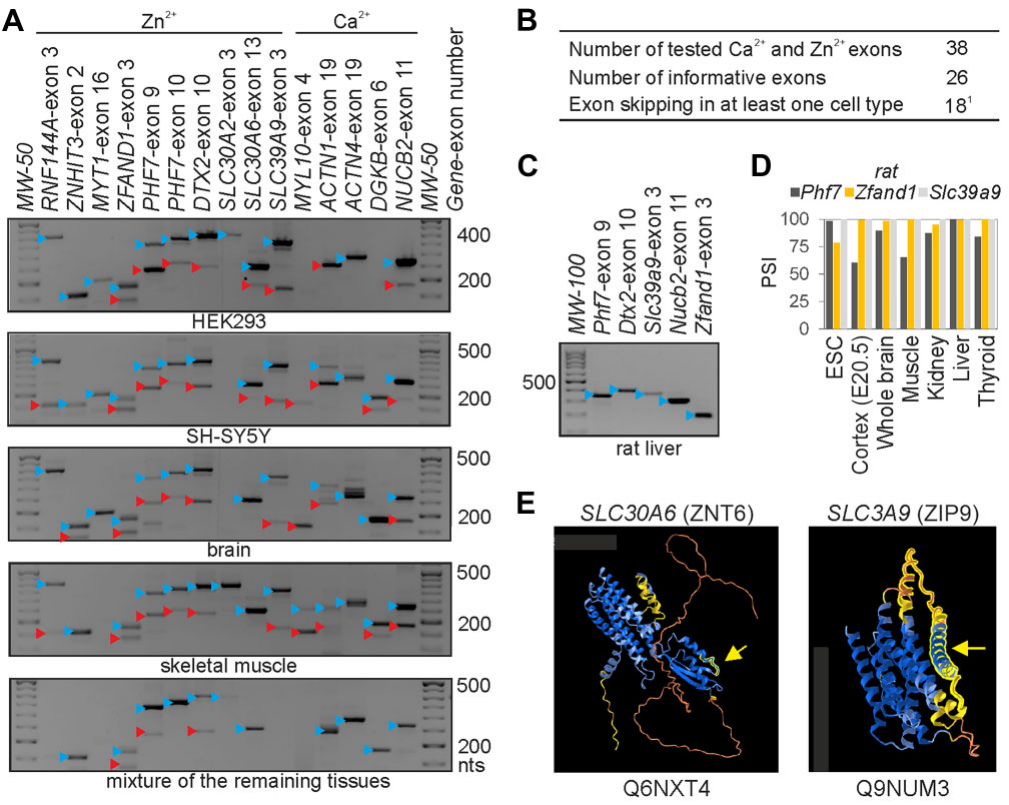
Validation of candidate Zn^2+^ or Ca^2+^ exons for regulated splicing.(**A**) Representative gel electrophoresis showing inclusion (blue triangles) or skipping (red triangles) of tested exons in the indicated cell lines and tissues. Sizes of spliced products (in nt) and primer sequences are given in Supplementary Table S2. The bottom panel shows splicing in a mixture of RNAs from adipose, bladder, cervix, colon, oesophagus, kidney, lung, ovary, pancreas, placenta, prostate, small intestine, testes and thymus. (**B**) Summary of validated alternatively spliced exons. ^1^Exons showing skipping in at least one tissue are identified in Supplementary Table S2. (**C**) Examples of a lack of exon skipping of rat orthologues. (**D**) VastDB PSI values for two rat exon homologues in a subset of rat tissues. (**E**) AlphaFold-predicted (136) structures of an efflux and influx Zn^2+^ transporter with alternatively spliced Zn^2+^ exons. Arrows point to accessible peptides (wrapped in yellow) that are absent in isoforms lacking the alternatively spliced exon.

Although alternative splicing of Zn^2+^ and Ca^2+^ exons is reduced compared with the average (Figure 2B, C), we detected a small subset of exons with low mean PSI values. To identify those that may be regulated, we tested 38 exons that had average VastDB PSI values < 90% in at least one of four tissues important for Ca^2+^/Zn^2+^ signalling. We designed RT–PCR primers (Supplementary Table S2) in exons adjacent to Zn^2+^ and Ca^2+^ exons and determined their mRNA inclusion in human neuroblastoma and embryonal kidney cell lines, and in human brain and skeletal muscle samples. The screening revealed exon skipping (red triangles in Figure 3A) for at least one transcriptome in 18 cases (47%; Figure 3B). Alternative splicing of these exons appeared to be more frequent in neural and muscle tissues than in the remaining tissues of the 16-tissue Ambion RNA panel. Only six exons revealed significant skipping in all four cell types. Alternative splicing of these exons was detected in other organisms for some but not all homologues or tissues (see example for the rat in Figure 3C and D; Supplementary Table S2), consistent with reduced transcriptomic diversity in less complex eukaryotes. Figure 3E shows examples of AlphaFold-predicted structures for two validated human exons in Zn^2+^ transporters, both with exposed alternatively spliced segments on the surface.

Together, these results identify exons encoding residues coordinating Zn^2+^ and Ca^2+^ that may be regulated by late-evolving alternative splicing events in a tissue- or developmental stage-specific manner.

### Selection of pyrimidines at position –3 relative to 3′ss of Zn^2+^ and Ca^2+^ exons

Although the poorer ESE/ESS profiles of Zn^2+^ exons as compared with Ca^2+^ exons were compensated by traditional splicing motifs (Figure 2D, H, I–L), there was one exception. We were puzzled by the excess of CAG 3′ss of Ca^2+^ exons over TAG 3′ss (Figure 2E). Human CAG 3′ss are more abundant than TAG 3′ss by a factor of > 2 (2.23 in our sample of introns) (68), but it is unclear why they are preferred. The two 3′ss are not functionally equivalent (65). Published case reports suggest that mutation –3C>T can induce exon skipping (65,69,70), promote exon inclusion (71,72), activate cryptic 3′ss (73) or remain silent (74–76). In line with these observations, mutation –3C>T induced opposite shifts in open and closed conformations of U2AF65 in different RNA contexts (66). However, binding of short CAG 3′ss RNAs to the wild-type yeast orthologue of U2AF35, which stabilizes U2AF65 PPT interactions (77,78, and references therein), was weaker as compared with UAG 3′ss RNAs, irrespective of the nucleotide identity at the first exon position (78). Can the observed difference in uridine and cytosine content at position –3 of Zn^2+^ and Ca^2+^ exons still be compensatory?

To address the question, we first compiled sequences of 38 published cases (Supplementary Table S5) and 39 unpublished ClinVar (79) records (Supplementary Table S6) of human –3C/T variants. In the latter collection, substitutions –3C>T were more prevalent than substitutions –3T>C (Supplementary Table S6, 32 versus 7, respectively, *P <*0.0001, binomial test). This might be due to a possible ascertainment bias toward more severe phenotypes or increased mutability of cytosines by deamination (80) and a progressive loss of cytosines at this position over time, but the resulting TAG might still provide a superior 3′ss in some cases. However, the bias was not observed for published phenotype-associated cases (Supplementary Table S5, 20 versus 18, respectively, *P*> 0.01) where the link between phenotype and mutation is tighter, further suggesting that substitutions –3T>C could create less efficient 3′ss in some transcripts.

**Figure 4.**
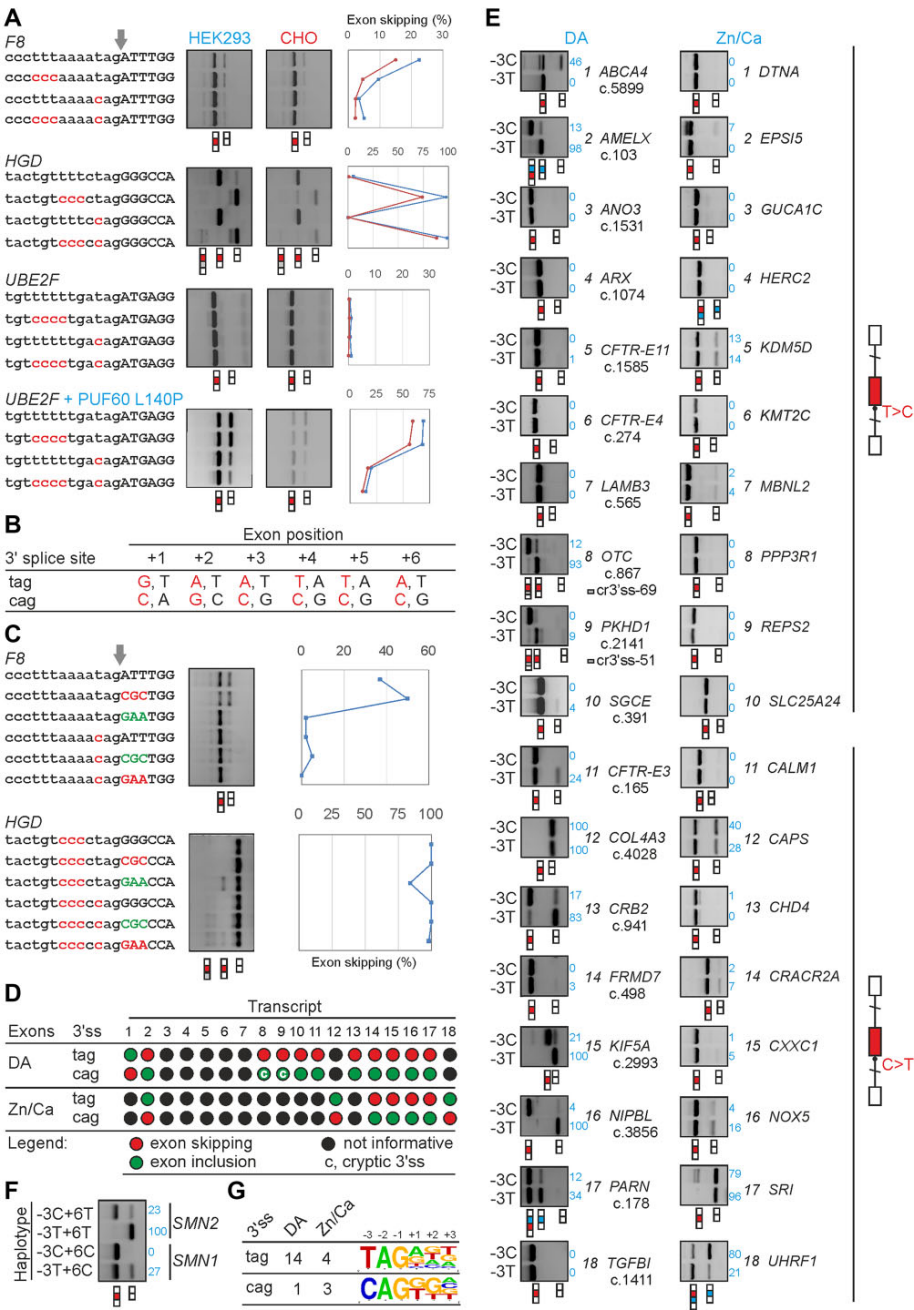
The human spliceosome prefers CAG 3′ss over TAG 3′ss. (**A**) Splicing outcome of mutations –3T> C and –3C>T in three minigene constructs in two cell lines. Arrow denotes 3′ss. Mutations are in red. Spliced products are shown at the bottom; tested mid-exons are red boxes. *HGD* produced cryptic 3′ss (grey box) 176 nt upstream (58) when the PPT was weakened. Differential skipping of *UBE2F* mid-exons was unmasked by co-expressing the reporter plasmid with equimolar amounts of uridine-binding PUF60 that had RRM1 substitution L140P or H169Y (57,99). Right panels show quantitation of exon skipping. (**B**) Allelic association between human TAG/CAG 3′ss and the first six exon positions. The most associated alleles are shown in red. The table is based on haplotypes in ∼195 000 introns of the reference human genome. (**C**) Exon skipping of reporters with TAG and CAG 3′ss carrying positively (green) and negatively (red) associated haplotypes at the first three exon positions. Right panels show exon skipping in HEK293 cells. (**D**) CAG 3′ss are preferred over TAG 3′ss in most informative transcripts. Supporting data are shown in (E). (**E**) Representative gels showing exon promotion or repression by mutations –3T>C (top) or –3C>T (bottom) introduced at the 3′ss of the indicated constructs. Wild-type and mutated constructs are schematically shown to the right: boxes are exons, vertical lines are introns, diagonal lines are restriction sites. The reporters were prepared for disease-associated (DA) mutations (left column) and Zn^2+^/Ca^2+^ exons (right column). Their numbering corresponds to (D). Spliced products are shown at the bottom of each panel: tested exons are in red, exons adjacent to tested exons in the pre-mRNA are in blue, exonic segments activated by cryptic 3′ss are in grey, and exon skipping measurements (%) are in blue. DA mutations are designated by c numbers that denote distances in nucleotides between the first position of the tested exon and adenine in the start codon of the longest open reading frame (Supplementary Table S5). (**F**) The impact of *SMN1/2* exon 7 variants at position +6 on the splicing outcome of closely linked C/T alleles at position –3 of exon 7 acceptor site. (**G**) Total number of informative TAG and CAG 3′ss that promoted exon skipping in tested constructs. The character height in logos depicts relative nucleotide frequencies at the indicated 3′ss positions.

To test this experimentally and to better understand the function of CAG and TAG 3′ss, we first explored our minigene library to select three informative reporters that produced skipping and inclusion of their mid-exons. We also weakened their PPTs in the presence of CAG or TAG 3′ss (Figure 4A; Supplementary Figure S11). Upon transient transfections into HEK293 or CHO cells, mutation –3T>C promoted exon inclusion in each transcript. In a PUF60-sensitive *UBE2F*, which has a very long PPT, we were able to see the distinct outcome of the two alleles only upon co-expression with mutated PUF60 (Figure 4A), possibly due to weakening 3′ss through reduced PUF60 binding to RNA or impaired balance between PUF60 and other splicing factors (57,99). Uridine-richer PPTs partially or fully restored the splicing defects, but they did not alter the direction of C>T mutations (Figure 4A). In *HGD*, weaker PPT was associated with the activation of upstream cryptic 3′ss, consistent with susceptibility of the *HGD* mid-exon to skipping in the presence of mutations in adjacent intronic sequences (58). The exon was fully skipped only in HEK293, but not in CHO cells (Figure 4A).

Positions within splice site consensus motifs are not independent (81). In the reference human genome, the –3T allele at 3′ss is positively associated with exon positions +1G, +2A, +3A, +4T, +5T and +6A (Figure 4B; Supplementary Figure S12). To test how the associated exon haplotypes influence the outcome of –3C/T variants and if exon mutations could reverse the observed differences in TAG and CAG 3′ss usage, we mutated minigenes *F8* and *HGD* at the first three exon positions in the presence of –3C or –3T (Figure 4C). In *F8*, the TAG 3′ss yielded stronger exon inclusion in the presence of the associated haplotype +G +2A +3A than the wild-type +1A +2T +3T or the CAG-associated haplotype +1C +2G +3C. Improved exon inclusion for TAG 3′ss in the presence of haplotype +1G +2A +3A was also found for *HGD*.

To expand the number of tested 3′ss, we then randomly selected a subset of disease-associated (DA) mutations –3C>T or –3T>C (Supplementary Table S5) and transcripts with Zn^2+^/Ca^2+^ exons (Supplementary Table S4). We cloned the tested exons and their flanking intronic sequences between two *U2AF1* exons to create hybrid reporters. We mutated the wild-type reporters to prepare an additional 18 DA and 24 Zn^2+^/Ca^2+^ minigene pairs that differed only by pyrimidine at position –3 (Figure 4D, E). Upon sequence validation and transfections into HEK293 cells, we found that of 11 informative DA exons, CAG 3′ss gave higher exon inclusion than TAG 3′ss in 10 transcripts whereas only one TAG 3′ss was clearly superior over CAG (binomial test, *P*= 0.005, Figure 4D). For *SMN1* haplotypes, mutation –3C>T at 3′ss of exon 7 produced exon skipping irrespective of exon 7 T/C variants at position +6, but the *SMN1*-specific exon 7 allele (+6C) partially rescued this defect (Figure 4F). We did not observe the bias for Zn^2+^/Ca^2+^ exons (Figure 4D, E, G) but the fraction of informative cases was much lower than for DA exons (29% versus 59%), consistent with their high PSI values in the native context (Figure 2B, C). Overall, counting all informative minigene pairs (*n* = 22), there was a significant excess of –3T over –3C among mutations leading to exon skipping (18 versus 4, *P*= 0.002, binomial test, Figure 4G).

Taken together, although a more abundant CAG is not always a 3′ss of choice for the human spliceosome, CAG 3′ss are preferred as they usually confer higher exon inclusion than TAG 3′ss. This information is fully encoded by minigene mid-exons and their native intronic flanks. Figure 4G also suggests that decisions as to which pyrimidine at position –3 activates or represses 3′ss are likely to be influenced by the auxiliary splicing code in exons, including the first exon position(s). We therefore suggest that the excess of CAG 3′ss in Ca^2+^ exons (Figure 2E) may still compensate the weaker exon position +1 in this exon group (Supplementary Figure S8), ensuring optimal U2AF35 binding to 3′ss YAG/R motifs.

### The Irving–Williams series, ESEs/ESSs and intrinsically disordered regions

**Figure 5.**
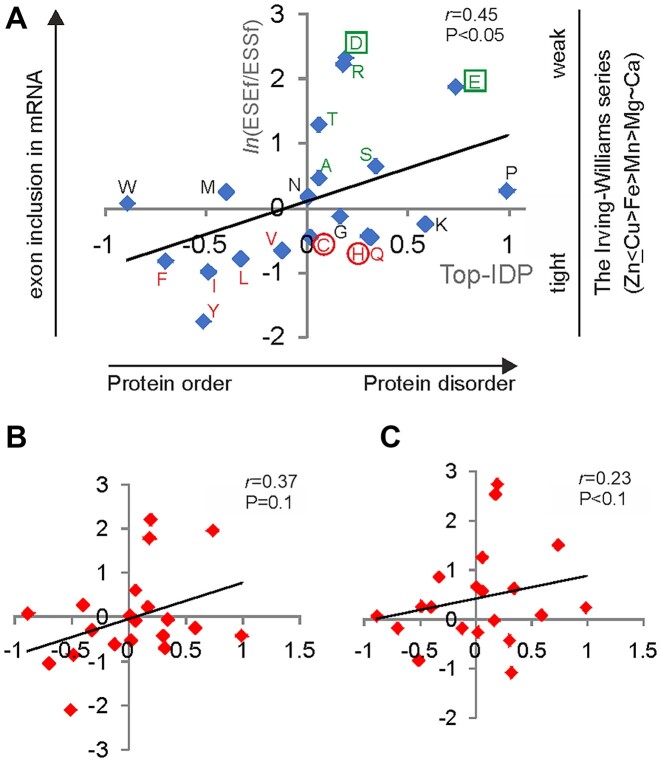
Auxiliary splicing code in exons, intrinsically disordered regions and the Irving–Williams series. (**A**) Correlation between the propensity to protein disorder and codon-specific exon inclusion levels assuming equal codon usage. Each diamond represents one amino acid. Codons for amino acids in green generally promote splicing, and codons for residues in red generally repress splicing on a continuous scale of ln(ESEf/ESSf) values (*y*-axis). Residues in green boxes are preferred ligands for weak divalent metals, and residues in red circles preferentially bind tighter metals in the Irving–Williams series. Top-IDP values (*x*-axis) predict IDRs (87). The correlation coefficient (top right) was similar for other disorder propensity scales (Table 3). (**B** and **C**) Diminished correlation of Top-IDP and *y*-weighted ln(ESE/ESS) values, reflecting codon usage in dividing (**B**) and differentiated cells (**C**). Codon usage data are from (88). The *y-* and *x*-axes are as in (A).

Alternative splicing tends to avoid protein domains while preferentially affecting intrinsically disordered regions (IDRs), polypeptide segments that do not acquire a defined tertiary structure autonomously but adopt diverse interconverting conformational states (82–86). Because this association may include domains that bind Zn^2+^ and Ca^2+^, we wished to explore the relationship between IDRs and codons for Zn^2+^- and Ca^2+^-coordinating residues. Under the assumption of an equal codon usage, codon-specific ESEf/ESSf values significantly correlated with various measures that predict protein disorder (Figure 5A). For example, one of the best performing measures, the TOP-IBD scale (87), showed a correlation coefficient of 0.45 while other measures of protein disorder yielded similar values (Table 3), confirming a general tendency of alternatively spliced regions to have higher ESE/ESS ratios and to be intrinsically disordered. The correlation is mainly driven by (i) exon-promoting codons for negatively charged amino acids (Asp, Glu), which coordinate weak divalent metals of the Irving–Williams series (Figure 1A); (ii) a subset of Arg, Thr, Ser and Ala codons, which were previously associated with protein disorder (87); and (iii) exon-repressing codons for Phe, Tyr and Ile/Leu/Val, which contribute to the protein order (Figure 5A). His and Cys residues, which coordinate tight metals, displayed inconsistent positions on some protein disorder scales (Supplementary Figure S13).

**Table 3.** Correlation between average ln(ESEf/ESSf) values for 20 amino acids and four measures of protein disorder

Average ln(ESEf/ESSf)^a^	Top-IDP	B-value	FoldUnfold	DisProt
Correlation coefficient	0.45	0.50	–0.56	0.55
*P*-value	0.047	0.020	0.009	0.011

^a^Average values were computed assuming equal codon usage. Weighted values were associated with increased *P*-values and diminished statistical significance (Figure 5B, C).

However, when the ESEf/ESSf values were weighted to reflect codon usage in gene categories involved in cell proliferation as opposed to differentiation (88), this correlation was reduced or disappeared (Figure 5B, C; Supplementary Figure S7). This observation is in line with a good correspondence between codon usage preferences and supply of tRNAs that were induced in proliferating cells and repressed in differentiating cells (88). There was no correlation between the molecular weight of amino acids and ESEf/ESSf values (Supplementary Figure S14A, B). Inclusion of both Zn^2+^ and Ca^2+^ exons is therefore influenced by codons encoding residues in the middle of the molecular weight spectrum and is largely supported by charged amino acids (D, E, R) while outermost residues tend to be splicing neutral (W, G).

## Discussion

### How splicing overcomes metal coordination constraints

This study demonstrates that exonic segments encoding Zn^2+^-coordinating amino acids have, on average, a significantly lower capacity to promote inclusion in mRNAs than those encoding Ca^2+^-coordinating residues. The two elements represent examples of tight and weak metals in the Irving–Williams series (Ca^2+^∼Mg^2+^ <Mn^2+^<Fe^2+^ <Cu^2+^≥Zn^2+^) (8). Because weak Mg^2+^ ions are preferentially coordinated to the same residues as Ca^2+^ and tight Cu^2+^ ions to the same residues as Zn^2+^ (89), it is reasonable to expect a similar ESE/ESS dichotomy for Mg^2+^ and Cu^2+^ (Figure 1A). However, exon datasets for protein-binding sites for these and other metals in the series are either much smaller or less well defined than those for Zn^2+^ or Ca^2+^. For example, the number of exons that encode Cu^2+^-coordinating residues in human copper proteins is currently an order of magnitude smaller than the number of Zn^2+^ exons in this study, but codon usage for their main coordinating amino acids is much more similar to Zn^2+^ than to Ca^2+^ (D.B. et al. ms in preparation).

These results support the existence of an ESE/ESS-mediated selection pressure that favours exons encoding binding sites for weak metals and disfavours exons encoding binding sites for tight metals. However, the extent to which such selection took place during evolution remains unknown. The Irving–Williams constraints were adopted by genetic codes of both prokaryotes and eukaryotes, which have similar residue preferences in their coordination spheres (His, Cys for tight metals, Glu, Asp for weak metals). However, pro- and eukaryotes have vastly distinct codon usage frequencies for these amino acids, which were linked to the expanding number of introns during eukaryotic evolution and which could affect ESE/ESS profiles. In addition, divalent metal bioavailability and cellular requirements dramatically changed during evolution, largely driven by photosynthesis-mediated oxygenation of euxinic sea and atmosphere (11). Arrival of Ca^2+^ signalling systems uniquely suitable for life came early and, at the exon level, they probably remained protected for over a billion years (Figure 2A). In contrast, addition of Zn^2+^ and Cu^2+^ to the environment took place later (11). These considerations warrant more detailed studies into the role of universal stabilities of these metals and their properties in the Periodic Table of Elements in shaping ESE/ESS-mediated splicing responses in other species (Figure 1A).

The ‘natural defence against tight metals’ embedded in the exonic splicing code is efficiently defeated by combinatorial diversity and flexibility of the splicing process *in vivo* (Figures 1 and 2). This would be consistent with the ability to overpower the Irving–Williams affinity order *in vitro* by flexible design of mutually exclusive metal-dependent protein conformation states (92). PSI values of both Zn^2+^ and Ca^2+^ exons were higher than those of average exons (Figure 2B, C), indicating that the cells evolved adaptive mechanisms at the exon level to safeguard Zn^2+^- and Ca^2+^-binding sites in the proteome. These adaptations over-ride the low capacity of ‘tight’ codons to promote inclusion of their exons in most, albeit not in all, cases (Figures 2 and 3). They include selection for stronger 5′ and 3′ss/PPTs but also stronger ESE/ESS profiles in the remaining exonic segments, at least for ZFs (Figure 2D, H–K). The compensation by splice site strength gauged by maximum entropy scoring appeared greater for 5′ss than for 3′ss (Figure 2D), in line with the central role of donor sites in splicing networks (90); nevertheless, we detected a robust effect of PPT composition albeit not of PPT length (Figure 2H–J). This suggests that the PPT position between the branchpoint and 3′ss is more important than PPT length when the number of pyrimidines is limiting, in line with a previous observation (91). Only ∼46% of control introns had optimal (U)_4_ motifs for U2AF65 binding downstream of predicted branchpoints and upstream of 3′ss (Supplementary Figure S10C).

### Candidate *trans*-acting factors involved in differential recognition of Zn^2+^ and Ca^2+^ exons

PPT/3′ss interact with U2AF, a tight and highly abundant heterodimer found in the early spliceosome assembly complex and required for recruitment of U2 small nuclear ribonucleoproteins (RNPs) (93,94). U2AF65 strongly favours uridine-rich sequences (94,95). Uridine excess and depletion preceding Zn^2+^ and Ca^2+^ exons, respectively, were observed for most PPT positions (Figure 2J). A significant depletion of (U)_4_ in strong (Ca^2+^) exons and over-representation in weak (Zn^2+^) exons (Supplementary Figure S10) also argues for a role for U2AF65, assuming that the (U)_4_ motif is the most favourable binding platform (67). In Zn^2+^ exons, the upstream peak of uridine frequencies extended towards branchpoints (Figure 2J) and a similar shift was observed as a compensation of poorly competing U2AF65 PPTs selected by enhanced cross-linking and immunoprecipitation (96). PPT uridines are preferred by both U2AF65 RRMs, but RRM1 appears to be more tolerant to cytidine and purine substitutions than RRM2, which is thought to interact with the upstream peak (97,98). Distinct PPTs preceding Zn^2+^ and Ca^2+^ exons may require distinct interactions with U2AF for accurate splicing, not excluding other factors involved in PPT-mediated splicing regulation, such as PUF60, TIA1/TIAR, PTB or hnRNP C (67,99, and references therein; *FAS* exon 6 in Supplementary Table S5). On the other hand, splicing *in vivo* may also benefit from mechanisms that delay multiple occupancy of candidate 3′ss by U2AF rather than from recruiting other factors (100). The compensation by intronic mutations improving PPTs of weak exons during evolution provides a more viable alternative to modifying exonic sequences. The auxiliary splicing code in exons *per se* may have been insufficient to compensate metal coordination constraints and ensure correct expression of mRNAs encoding metal-binding sites. This highlights thus far unknown function of introns in splicing of pre-mRNAs that encode metalloproteins.

Although 3′ss recognition depends, to a large extent, on uridine fractions in PPT (94), weak PPT may be partially overcome by strong branchpoints (95). Even if no obvious changes in branchpoint consensus and distance from 3′ss between ZnBPs and CaBPs were observed, we cannot formally exclude this possibility given the highly degenerate nature of a short branchpoint sequence consensus (101), suboptimal accuracy of branchpoint prediction tools (45), possible tissue-specific branchpoint use (102), discordant branchpoint assignments by computational versus experimental approaches (28) and the existence of multiple, distant and non-canonical branchpoints (28,47,48,103,104). The limited fraction of concordant branchpoints identified by the three methods (90 among 240 informative cases; Supplementary Dataset S6) reduces the exclusion power of branchpoint comparisons, but discordant branchpoint assignments did not invalidate the significant difference in uridine content in PPTs between Zn^2+^ and Ca^2+^ exons. On the other hand, a lack of evidence for a compensatory role for branchpoint motifs would be consistent with a lack of conformational shifts of U2AF65 in the presence of branchpoint-bound splicing factor 1 (77, and references therein).

Candidate *trans*-acting factors that recognize RNA motifs containing codons for main Zn^2+^- and Ca^2+^-coordinating residues are listed in Supplementary Table S8.

Together, we propose that U2AF65 interactions with stronger PPTs of Zn^2+^ exons may help achieve high exon inclusion and defy ESE/ESS restrictions imposed by Zn^2+^ coordination. Future studies should address if stronger splice sites of Zn^2+^ exons are sufficient to overcome their poor ESE/ESS profiles or if they require additional features in their intron–exon architecture, such as pre-mRNA structures.

### Position –3 relative to 3′ splice sites in Zn^2+^ and Ca^2+^ exons

The increased binding affinity of U2AF65 for uridine-richer PPTs correlates with a shift from closed to open or active conformation required for spliceosome assembly, which is stabilized by U2AF35 (77, and references therein). U2AF35 recognizes both UAG and CAG 3′ss (78). However, the higher binding affinity of U2AF35 for UAG than for CAG or RAG RNAs (R = purine), which was detected by isothermal calorimetry irrespective of the nucleotide identity at the first exon position (78), seems incompatible with a clear preference for human CAG 3′ss (Figure 4D–F) and it is unclear if it could explain their excess in Ca^2+^ exons either (Figure 2E). The 3′ss YAG/R motifs are bound by both U2AF35 ZFs, but position –3 mainly interacts with ZF1 via R28, H29, S34 and R35 (78). These residues are in the vicinity of Zn^2+^-coordinating cysteines C27 and C33. Adjacent S34 is a hot spot for cancer-associated substitutions S34F and S34Y (105). In the complex of wild-type *Saccharomyces cerevisiae* U2AF35 with UAG 3′ss, –3U is stacked with the imidazole ring of H29 and surrounded by S34, R35 and a short ZF1 helix, whereas the pocket accepting the –3 base is too shallow to accommodate purines (78). Replacement of –3U by –3C was associated with a loss of hydrogen bond to S34, with the S34 mutants showing less discrimination at position –3 (78). The distribution of CAG and UAG 3′ss was reported to be altered in tumours carrying mutation U2AF35 S34F, with the S34F mutant preferentially binding CAG 3′ss (106,107, and references therein). The higher affinity of the wild-type *S. cerevisiae* U2AF35 for UAG as compared with CAG RNAs (78) could reflect a predominance of UAG 3′ss in some yeasts, whereas multicellular organisms have been generally associated with a higher abundance of CAG 3′ss, including almost invariant CAG 3′ss in some nematodes (68,90,108,109). This notion is supported by a dramatically better splicing of UAG 3′ss over CAG 3′ss of a fission yeast *cdc2* intron (109). Although the excess of CAG 3′ss in human Ca^2+^ exons could compensate weaker adenines at position +1 (cf. Figure 2E and Supplementary Figure S8), adenine at this position was absent in a small number of CAG 3′ss that induced more exon skipping than UAG 3′ss (Figure 4G).

### Prediction of splicing defects for pyrimidine transitions at position –3 relative to 3′ss

Our results improve prediction of splicing and phenotypic outcomes of T/C variants at position –3. First, their effect on splicing clearly depends on intron type. This is exemplified by a 2 nt insertion between adjacent *SCN1A* exons due to mutation c.4477–3T>C in the AT–AC type intron (Supplementary Table S5). Since this intron ends with AC and not AG (ttttcta[–3t>c]/acTTTGGAGG, where / is a new 3′ss), the mutation replicates the TAC 3′ss 2 nt upstream, creating a frameshift by inserting an extra AC dinucleotide into the mRNA. In a canonical GT–AG intron, the same mutation could not create the new splice site. Second, mutations –3C>T are more likely to result in exon skipping than –3T>C (Figure 4D, E). Although it is unclear why it is not always the case, the compiled lists (Supplementary Tables S5 and S6) will be useful in future studies of functional differences of CAG and TAG 3′ss. Third, even if mutations –3T/C do not alter splicing of exogenous transcripts (Figure 4D, E), they may have other unforeseen effects *in vivo* that may be cell type specific, such as cryptic 3′ss activation (Figure 4A, E). Many disease-associated –3C/T mutations were found in the last introns (Supplementary Table S5), potentially affecting gene termination. Fourth, the PPT strength can partially or completely compensate for a weaker CAG or TAG 3′ss (Figure 4A, C). Fifth, the first position of exons as well as other exonic positions often modulate the splicing outcome of mutations –3T/C (Figure 4C). U2AF35 interactions with the 3′ss YAG/R consensus may extend to or depend on a downstream ESE (110), suggesting that exon variants closely linked to mutations –3C/T (111,112; Supplementary Table S5) could help identify U2AF35-mediated interactions between position –3 and exonic sequences. Although *ab initio* prediction of phenotypic consequences of pyrimidine transitions at position –3 relative to 3′ss is futile at present, this guidance should improve it.

### Evolution of exons encoding binding sites for divalent metals

Given the high ESE/ESS conservation (18), differences in ESE/ESS profiles in human Zn^2+^ or Ca^2+^ exons are likely to be replicated in other vertebrates. Alternative splicing of selected human Zn^2+^ or Ca^2+^ exons was, however, only partially recapitulated in the rat (Figure 3C, D) even though each rat coordinating codon in tested exons was identical to human counterparts.

The total content of the six metal elements (Figure 1A) is similar in animals and plants (except for a higher Mn^2+^ in the latter) (11), raising questions about metal-dependent evolution of the splicing code in other multicellular organisms. The number of proteins that bind four of the six elements (Ca^2+^, Mg^2+^, Cu^2+^and Zn^2+^) increased very rapidly in multicellular eukaryotes (11).

Compensatory interactions between ESEs/ESSs and splice sites of Zn^2+^ and Ca^2+^ exons (Figure 2) support exons as primary evolutionary units in eukaryotes (90). The greater promotion of exonic sequences for Ca^2+^-binding sites by their ESEs/ESSs than for Zn^2+^-binding sites may have contributed to their very rapid spread when the first eukarya emerged (113). Cell type-specific expression of CaBPs could be better regulated by a combination of weak traditional and strong auxiliary splicing signals (Figures 1 and 2). Eukaryotic proteomes contain a greater fraction of Zn^2+^- and Ca^2+^-binding structures than those binding iron, and the eschewal of iron for Zn^2+^ and Ca^2+^ during evolution was their defining feature (113).

The hypothesis that evolution of the auxiliary splicing code in exons was responsive to the Irving–Williams constraints (Figure 1A) relies on two assumptions. First, codon frequencies at coordination spheres for weak metals are distinct from those for tight metals (Figure 1A). Second, PDB structures for metalloproteins largely reflect Zn^2+^ and Ca^2+^ binding *in vivo*. While the first assumption appears to be solid (89, and references therein), there are multiple examples where the latter supposition does not hold. Existing structural models may have employed procedures that led to incorrect metal assignments, including the use of chelating agents, improper ion concentration ranges during protein purification procedures or artificial culture growth conditions (9). Apart from metal properties such as redox state, the metal content in a protein depends on species, cell type and subcellular location, restricting or promoting metal bioavailability (9). In eukarya, subcellular compartmentalization of protein interactions and their rewiring can be determined by alternative exon usage (114), but examples of functional isoforms that are alternatively spliced to include or exclude metal-binding sites have been scarce. Our screening of low-PSI Zn^2+^ and Ca^2+^ exons confirmed alternative splicing in 18 cases (Figure 3), suggesting that only a limited number of these exons is expendable without altering fitness. Known examples include alternative splicing of *SLC30A2* (ZnT2) exon 3 (Figure 3A), which generates two mRNA isoforms in mammary glands that differ by 49 amino acids and a transmembrane domain of the antiporter (115). Both isoforms are functional and have a distinct subcellular location (115). Alternative splicing regulates other antiporters and uniporters, such as KEA3 via the C-terminal KTN domain (116) or MICU1 via a small microexon (117). Canonical or major isoforms are highly abundant for some proteins (115,116) but not for others (117). The importance of subcellular location in differential metal binding was also supported for Mia40 (*CHCHD4*) (118), MitoNEET (*CISD1*) (119) or Miner1 (*CISD2*) (120), but the overall number of these cases remains low.

### A gradient of ESE/ESS profiles for codons encoding Zn^2+^-coordinating residues

A growing fraction of proteins has been reported to bind more than one metal element, and binding of one divalent metal can influence binding of another. For example, Zn^2+^-binding sites in S100 proteins can bind Cu^2+^, which could be displaced by Zn^2+^, but not by Ca^2+^ (121). Binding of tight Cu^2+^ to S100A5 impairs binding of the EF-hand to Ca^2+^ (122). High Zn^2+^ concentrations can displace Mg^2+^ from the EF-hands and alter stoichiometry of Ca^2+^ binding (123). Elevation of extracellular Zn^2+^ can increase intracellular Ca^2+^ and inhibit calmodulin (124, and references therein). Zn^2+^ binding may enhance the affinity for target peptides over the effect of Ca^2+^ alone (125). Zn^2+^ binding to S100 EF-hands can alter the geometry of the Ca^2+^-binding loop and Ca^2+^ affinity (126) although the effects of Zn^2+^ binding on the structure of S100 proteins appear to be generally more modest than those elicited by Ca^2+^ binding (121). Binding of both tight and weak metals by a single protein might reduce selection pressures to exclude tight codons from mature transcripts. The intermediate ESE/ESS score values for S100 and other proteins that bind non-Zn^2+^ metals (Figure 1C–E) suggest that codons for acidic residues compensate low exon inclusion levels typical of coordinating codons for Zn^2+^ (Figure 1C–E) and other tight binders (Figure 1A). During evolution, the proportion of ZFs in proteomes increased from ∼0.2% in archaea to 1.9% in yeast and > 3% in eukarya (127), yet their low exon inclusion potential did not impede ZF spread in intron-containing organisms and was compensated to some degree at the ESE/ESS level (Figure 2A). Crystal structures of the best studied bacterial ZIP ancestor, ZIPB, showed coordination spheres distinct from a classical Zn^2+^ tetrahedral site, with a more complex binuclear site and coordination numbers ranging from 4 to 6 (62). The Zn^2+^ transport sites of ZIPB or YiiP employ acidic residues rather His/Cys (Supplementary Figures S2 and S3), bridging separate ions and giving the coordination spheres higher flexibility. In addition, alternative acidic residues may confer promiscuous metal selectivity (62). Thus, acidic residues have important roles in ZnBPs, and their codons would compensate low exon inclusion levels of associated Cys/His codons. Codons for acidic residues at Zn^2+^ interfaces may account for 18–19% of the total (128,129), a figure that may have a global impact on exon inclusion.

### Does the exonic splicing code reduce mismetalation?

To safeguard codons for residues coordinating both weak and tight metals in mature transcripts, the splicing code evolved to repress codons for tighter metals and promote codons for weak metals, potentially reducing mismetalation in the cell. Mismetalation coincides with altered metal bioavailability, which is believed to be a crucial contributor to its avoidance (1, and references therein). Even in prokaryotes, bioavailability in cyto- and periplasmic compartments can restrict competition between tighter binding Cu^2+^ and weaker Mn^2+^ cupins (130). In higher eukaryotes, bioavailability can be substantially controlled by regulated splicing decisions that restrict or permit protein–protein interactions by introducing distinct protein isoforms with distinct post-translation modification, folding or intrinsically disordered patterns (86) (Figure 3E). Zn^2+^ may bind differently to ordered and disordered regions (Supplementary Table S9), nevertheless it is as yet unclear if post-translational modifications of metal-coordinating residues such as His phosphorylation could play a role in reducing mismetalation. The location of protein folding can also determine the specificity of metalation, with correct or incorrect metals kinetically trapped within folded proteins (130). Importantly, metal availability follows, or tends toward, the inverse of the Irving–Williams series (131) and also appears to reflect metal concentrations estimated for euxinic sea waters (Figure 1A, bottom).

In conclusion, our results provide new insights into the intertwined protein-coding, splicing and metal codes in human DNA and most probably in DNA of other mammals and higher vertebrates. The universal order of metal binding preferences is difficult for evolution to circumvent (132), forcing the cell to exploit both the ‘invisible’ exonic splicing code and traditional splicing motifs in introns to ensure adequate expression of metalloproteins that bind weak or competitive divalent metals.

## Supplementary Material

gkad1161_supplemental_files

## Data Availability

The authors confirm that the data supporting the findings in this study are available within the article and its supplementary data.
