# Supplementary material for: Exonic splicing code and coordination of divalent metals in proteins
Source: Nucleic Acids Res. 2023 Dec 6;52(3):1090–106. doi: 10.1093/nar/gkad1161 (PMC10853796; doi:10.1093/nar/gkad1161)
Supplement: gkad1161_supplemental_files [file gkad1161_supplemental_files.zip › Supplementary information-FORMATTED-revised.pdf]

SUPPLEMENTARY INFORMATION

Exonic splicing code and coordination of divalent metals in proteins

Dara Bakhtiar[1], Katarina Vondraskova[2], Reuben Pengelly[1], Martin Chivers[1], Jana Kralovicova[1,2], Igor Vorechovsky[1,¶]

[1]University of Southampton
Faculty of Medicine
Southampton SO16 6YD
United Kingdom

[2]Slovak Academy of Sciences
Centre of Biosciences
840 05 Bratislava
Slovak Republic

SUPPLEMENTARY FIGURES

Figure S1 Amino acid alignment of ZnBPs: human S100 proteins

In all alignments in Figures S1-S4, ESE/ESS scores were computed for codons coding for residues highlighted in yellow as shown in Figure 1B. Codons are identified in Datasets S1-S4. The alignments were carried out with Clustal Omega (v. 1.2.4) (1), allowing for a maximum number of iterations. Colour-coding for Zn^{2+}-coordinating residues is as follows: Protein Databank (PDB) access code 4YBH (S100A6), 2PSR (S100A7), 4AQI (S100A7A), 4GGF (S100A8 and S100A9), 2WC8 (S100A12) and 3D0Y (S100B). N-terminal pseudo-EF hands and C-terminal canonical EF hands are highlighted in grey. Because amino acids other than C, H, E and D may coordinate Zn^{2+}, including Q (2), R (3) and S (4), their codons were included in ESE/ESS profiling if they occupied the same alignment positions as in Zn^{2+}-binding PDB structures.

```
S100A1  |-------------MGSELETAMETLINVFHAHSGKEG-DKYKLSKKELKELLQTELSGFL
S100A2  |------------MMCSSLEQALAVLVTTFHKYSCQEG-DKFKLSKGEMKELLHKELPSFV
S100A3  |-------------MARPLEQAVAAIVCTFQEYAGRCG-DKYKLCQAELKELLQKELATWT
S100A4  |-------------MACPLEKALDVMVSTFHKYSGKEG-DKFKLNKSELKELLTRELPSFL
S100A5  |-------------METPLEKALTTMVTTFHKYSGREG-SKLTLSRKELKELIKKELC--L
S100A6  |-------------MACPLDQAIGLLVAIFHKYSGREG-DKHTLSKKELKELIQKELT--I
S100A7  |------------MSNTQAERSIIGMIDMFHKYTRRDD----KIEKPSLLTMMKENFPNFL
S100A7A |------------MSNTQAERSIIGMIDMFHKYTGRDG----KIEKPSLLTMMKENFPNFL
S100A8  |-------------MLTELEKALNSIIDVYHKYSLIKG-NFHAVYRDDLKKLLETECPQYI
S100A9  |----------MTCKMSQLERNIETIINTFHQYSVKLG-HPDTLNQGEFKELVRKDLQNFL
S100A10 |-------------MPSQMEHAMETMMFTFHKFAGDKG----YLTKEDLRVLMEKEFPGFL
S100A11 |--------MAKISSPTETERCIESLIAVFQKYAGKDG-YNYTLSKTEFLSFMNTELAAFT
S100A12 |--------------MTKLEEHLEGIVNIFHQYSVRKG-HFDTLSKGELKQLLTKELANTI
S100A13 |---------MAAEPLTELEESIETVVTTFFTFARQEG-RKDSLSVNEFKELVTQQLPHLL
S100A14 |MGQCRSANAEDAQEFSDVERAIETLIKNFHQYSVE-G-GKETLTPSELRDLVTQQLPHLM
S100A16 |----------MSDCYTELEKAVIVLVENFYKYVSKYSLVKNKISKSSFREMLQKELNHML
S100B   |--------------MSELEKAMVALIDVFHQYSGREG-DKHKLKKSELKELINNELSHFL
S100G   |---------------MSTKKSPEELKRIFEKYAAKEG-DPDQLSKDELKLLIQAEFPSLL
S100P   |--------------MTELETAMGMIIDVFSRYSGSEG-STQTLTKGELKVLMEKELPGFL
S100Z   |-------------MPTQLEMAMDTMIRIFHRYSGKER-KRFKLSKGELKLLLQRELTEFL

S100A1  |DAQ--KDVDAVDKVMKELDENGDGEVDFQEYVVLVAALTVACNNFFWENS----------
S100A2  |GEK--VDEEGLKKLMGSLDENSDQQVDFQEYAVFLALITVMCNDFFQGCPDRP-------
S100A3  |PTE--FRECDYNKFMSVLDTNKDCEVDFVEYVRSLACLCLYCHEYFKDCPSEPPCSQ---
S100A4  |GKR--TDEAAFQKLMSNLDSNRDNEVDFQEYCVFLSCIAMMCNEFFEGFPDKQPRKK---
S100A5  |GE---MKESSIDDLMKSLDKNSDQEIDFKEYSVFLTMLCMAYNDFFLEDNK---------
S100A6  |GSK--LQDAEIARLMEDLDRNKDQEVNFQEYVTFLGALALIYNEALKG------------
S100A7  |SACDKKGTNYLADVFEKKDKNEDKKIDFSEFLSLLGDIATDYHKQSHGAAPCSGGSQ---
S100A7A |SACDKKGIHYLATVFEKKDKNEDKKIDFSEFLSLLGDIAADYHKQSHGAAPCSGGSQ---
S100A8  |RKK------GADVWFKELDINTDGAVNFQEFLILVIKMGVAAHKKSHEESHKE-------
S100A9  |KKEN-KNEKVIEHIMEDLDTNADKQLSFEEFIMLMARLTWASHEKMHEGDEGPGHHHKPGLGEGTP
S100A10 |ENQ--KDPLAVDKIMKDLDQCRDGKVGFQSFFSLIAGLTIACNDYFVVHMKQKGKK----
S100A11 |KNQ--KDPGVLDRMMKKLDTNSDGQLDFSEFLNLIGGLAMACHDSFLKAVPSQKRT----
S100A12 |KNI--KDKAVIDEIFQGLDANQDEQVDFQEFISLVAIALKAAHYHTHKE-----------
S100A13 |KD-----VGSLDEKMKSLDVNQDSELKFNEYWRLIGELAKEIRKKKDLKIRKK-------
S100A14 |PS-----NCGLEEKIANLGSCNDSKLEFRSFWELIGEAAKSVKLE------RPVRGH---
S100A16 |SDT--GNRKAADKLIQNLDANHDGRISFDEYWTLIGGITGPIAKLIHEQEQQSSS-----
S100B   |EEI--KEQEVVDKVMETLDNDGDGECDFQEFMAFVAMVTTACHEFFEHE-----------
S100G   |KG-----PNTLDDLFQELDKNGDGEVSFEEFQVLVKKISQ--------------------
S100P   |QSG--KDKDAVDKLLKDLDANGDAQVDFSEFIVFVAAITSACHKYFEKAGLK--------
S100Z   |SCQ--KETQLVDKIVQDLDANKDNEVDFNEFVVMVAALTVACNDYFVEQLKKKGK-----
```

Figure S2 Amino acid alignment of ZnBPs: human Zn^{2+} transporters and bacterial YiiP

Zn^{2+}-coordinating residues in ZnT5 (5), ZnT8 (6) and YiiP (PDB access code 3H90) are shown in red. YiiP contains three unique binding sites, termed ZnA, ZnB and ZnC (7). Zn^{2+}-coordinating residues in ZnA are highlighted in blue (D45, D49 in TM2 and H153 and D157 in TM5); Zn^{2+}-coordinating residues in ZnB are highlighted in green (D68, H71 and H75). Coordinating residues in ZnC are highlighted in light grey, with H261 and H283 interacting with the first Zn^{2+} ion and H232 and H248 with the second Zn^{2+} ion. The binuclear core of ZnC is bridged by D285 (dark grey).

Human ZnT8 contains four binding sites in each subunit (6). In the primary binding site in the transmembrane domain (TMD), Zn^{2+} is coordinated by H106, D110, H220 and D224 (6). In the site on the interface of TMD and C-terminal domain (CTD), Zn^{2+} is contacted by H137 and H345. At CTD1 and CTD2 sites, Zn^{2+} is coordinated by H52, H54, C361 and C364, and H301, H318, E352 and C53, respectively (6). D599 in ZnT5 was proposed as a Zn^{2+} binding residue following mutagenesis (5).

```
ZNT1          -----------------------------------------------MGCWGRNRGRLLC
ZNT2          ---------------DLQAIELAAQSNHHCH-------AQKGPDSHCDPKKGKAQRQLYV
ZNT3          ---------------------PVEMPFHHCH-------RDPLPPPGLTPERLHARRQLYA
ZNT4          DDDSLLDQDL-----PLTNSQL-SLKVDSC---------DNCSKQREILKQRKVKARLTI
ZNT5          EGTPLYNFM-----------GDAFQHSSQSI-------PRFIKESLKQILEESDSRQIFY
ZNT6          -----------------MGTIHLFRKPQRSF-------FGKLLREFRLVAADRRSWKILL
ZNT7          --MLPLSIK-----------DDEYKPPKFNL-------FGKISGWFRSILSDKTSRNLFF
ZNT8          ---------------E----ELESGGMYHCH-------SGSKPTEKGANEYAYAKWKLCS
ZNT9          SPEALAREKKLRKEAEIEYRERLFRNQKILREYRDFLGNTKPRSRTASVFFKGPGKVVMV
ZNT10         -----------------------------------------------MGRYSGKTCRLLF
YIIP          ---------------------------------------------MNQSYGRLVSRAAIA


ZNT1          MLALTFMFMVLEVVVSRVTSSLAMLSDSFHMLSDVL------------------------
ZNT2          ASAICLLFMIGEVVGGYLAHSLAVMTDAAHLLTDFA------------------------
ZNT3          ACAVCFVFMAGEVVGGYLAHSLAIMTDAAHLLADVG------------------------
ZNT4          AAVLYLLFMIGELVGGYIANSLAIMTDALHMLTDLS------------------------
ZNT5          FLCLNLLFTFVELFYGVLTNSLGLISDGFHMLFDCS------------------------
ZNT6          FGVINLICTGFLLMWCSSTNSIALTAYTYLTIFDLFRDGVSPFWLGWSQTPDLKWSTHLG
ZNT7          FLCLNLSFAFVELLYGIWSNCLGLISDSFHMFFDST------------------------
ZNT8          ASAICFIFMIAEVVGGHIAGSLAVVTDAAHLLIDLT------------------------  (H106,D110)
ZNT9          AICINGLNCFFKFLAWIYTGSASMFSEAIHSLSDTC------------------------
ZNT10         MLVLTVAFFVAELVSGYLGNSIALLSDSFNMLSDLI------------------------
YiiP          ATAMASLLLLIKIFAWWYTGSVSILAALVDSLVDIG------------------------
                 :        ..      . .: :     : *

ZNT1          ----------------ALVVALVAERFARRTHATQKNTFGWIRAEVMGALVNAIFL-TGL
ZNT2          ----------------SMLISLFSLWMSSRP-ATKTMNFGWQRAEILGALVSVLSI-WVV
ZNT3          ----------------SMMGSLFSLWLSTRP-ATRTMTFGWHRSETLGALASVVSL-WMV
ZNT4          ----------------AIILTLLALWLSSKS-PTKRFTFGFHRLEVLSAMISVLLV-YIL
ZNT5          ----------------ALVMGLFAALMSRWK-ATRIFSYGYGRIEILSGFINGLFL-IVI
ZNT6          LPKCWDNRRELPCLSNSLMTCLISYWVTLRK-PSPVYSFGFERLEVLAVFASTVLA-QLG
ZNT7          ----------------AILAGLAASVISKWR-DNDAFSYGYVRAEVLAGFVNGLFL-IFT
ZNT8          ----------------SFLLSLFSLWLSSKP-PSKRLTFGWHRAEILGALLSILCI-WVV  (H137)
ZNT9          ----------------NQGLLALGISKSVQT-PDPSHPYGFSNMRYISSLISGVGIFMMG
ZNT10         ----------------SLCVGLSAGYIARRPTRGFSATYGYARAEVVGALSNAVFL-TAL
YiiP          ----------------ASLTNLLVVRYSLQP-ADDNHSFGHGKAESLAALAQSMFI-SGS
                                         :          :*  . . :. : . :
```

```
ZNT1       CFAILLEAIERFIEPHEMQQPLVVLGVGVAGLLVNVLGLCLFHHHSGFSQDSGHGHSHGG
ZNT2       TGVLVYLAVERLISGDYEIDGGTMLITSGCAVAVNIIMGLTLHQ-------SGHGHSHGT
ZNT3       TGILLYLAFVRLLHSDYHIEGGAMLLTASIAVCANLLMAFVLHQ-------AGPPHSHGS
ZNT4       MGFLLYEAVQRTIHMNYEINGDIMLITAAVGVAVNVIMGFLLNQ-------SGHRHSHSH
ZNT5       AFFVFMESVARLIDPPELD-THMLTPVSVGGLIVNLIGICAFSH--------AHSHAHGA
ZNT6       ALFILKESAERFLEQPEIH-TGRLLVGTFVALCFNLFTMLSIRN-------KPFAYVSEA
ZNT7       AFFIFSEGVERALAPPDVH-HERLLLVSILGFVVNLIGIFVFKH-------GGHGHSHGS
ZNT8       TGVLVYLACERLLYPDYQIQATVMIIVSSCAVAANIVLTVVLHQ-------RCLGHNHKE
ZNT9       AGLSWYHGVMGLLHPQPIESLLWAYCILAGSLVSEGATLLVAVNEL--------------
ZNT10      CFTIFVEAVLRLARPERIDDPELVLIVGVLGLLVNVVGLLIFQDCAAWFACCLRGRSRRL
YiiP       ALFLFLTGIQHLISPTPMTDPGVGVIVTIVALICTIILVSFQRWVV--------------
                  .                      ..

ZNT1       -------HGHGHGLPKGPRVKSTRPGSSDINVAPGEQGPDQEETNTLVANTSNSNGLKLD
ZNT2       TNQQ--------------------------------------------------------
ZNT3       RGAE--------------------------------------------------------
ZNT4       SLPS--------------------------------------------------------
ZNT5       ------SQGSCHS-----------------------------------SDHSHSHHMHG-
ZNT6       A------STSWLQE----------------------------------------------
ZNT7       ------GHGHSHSLFNGALDQA-----------------------HGHVDHCHSHEVKHG
ZNT8       VQ----------------------------------------------------------
ZNT9       ------------------------------------------------------------
ZNT10      QQRQQLAEGCVPGAFGGPQGAE-DPRRAADPTAPGSDSAVT----------------LRG
YiiP       ------------------------------------------------------------

ZNT1       PADPENPRSGDTVEVQVNGN---LVREPDHMELEEDRAGQLNMRGVFLHVLGDALGSVIV
ZNT2       -----------------------E--------------ENPSVRAAFIHVIGDFMQSMGV
ZNT3       -----------------------YAP-LEEGPEEPLPLGNTSVRAAFVHVLGDLLQSFGV
ZNT4       -----------------------NSPTRGSGCERNHGQDSLAVRAAFVHALGDLVQSVGV
ZNT5       -----HSDHGHGHSHGSAGG----------G-------MNANMRGVFLHVLADTLGSIGV
ZNT6       ----------------------HVADLSRSLCGIIPGLSSIFLPRMNPFVLIDLAGAFAL
ZNT7       AAHSHDHAHGHGHFHSHDGP----------SLKETTGPSRQILQGVFLHILADTLGSIGV
ZNT8       --------------------------------------ANASVRAAFVHALGDLFQSISV  (H220,D224)
ZNT9       -----------------------RRNARAKGMSFYKYVMESRDPSTNVILLEDTAAVLGV
ZNT10      TSVERKREKGATVFANVAGDSFNTQNEPEDMMKKEKKSEALNIRGVLLHVMGDALGSVVV
YiiP       ----------------------------------RRTQSQAVRADMLHYQSDVMMNGAI
                                                               *      :

ZNT1       VVNALVFYFSWKGCSEGDFCVNPCFPDPCKAFVEIINSTHASVYEAGPCWVLYLDPTLCV
ZNT2       LVAAYILYFKP--------------------------------------EYKYVDPICTF
ZNT3       LAASILIYFKP--------------------------------------QYKAADPISTF
ZNT4       LIAAYIIRFKP--------------------------------------EYKIADPICTY
ZNT5       IVSTVLIEQF---------------------------------------GWFIADPLCSL
ZNT6       CITYMLIEIN---------------------------------------NYFAVDTASAI
ZNT7       IASAIMMQNF---------------------------------------GLMIADPICSI
ZNT8       LISALIIYFKP--------------------------------------EYKIADPICTF
ZNT9       IIAATCMGLTSIT------------------------------------GNPLYDSLGSL
ZNT10      VITAIIFYVLPLK------------------------------SEDPCNWQCYIDPSLTV
YiiP       LLALGLSWYG----------------------------------------WHRADALFAL
                                                                 *

ZNT1       VMVCILLYTTYPLLKESALILLQTVPKQID--IRNLIKELRNVEGVEEVHELHVWQLAGS
ZNT2       VFSILVLGTTLTILRDVILVLMEGTPKGVD--FTAVRDLLLSVEGVEALHSLHIWALTVA
ZNT3       LFSICALGSTAPTLRDVLRILMEGTPRNVG--FEPVRDTLLSVPGVRATHELHLWALTLT
ZNT4       VFSLLVAFTTFRIIWDTVVIILEGVPSHLN--VDYIKEALMKIEDVYSVEDLNIWSLTSG
ZNT5       FIAILIFLSVVPLIKDACQVLLLRLPPEYEKELHIALEKIQKIEGLISYRDPHFWRHSAS
ZNT6       AIALMTFGTMYPMSVYSGKVLLQTTPPHVIGQLDKLIREVSTLDGVLEVRNEHFWTLGFG
ZNT7       LIAILIVVSVIPLLRESVGILMQRTPPLLENSLPQCYQRVQQLQGVYSLQEQHFWTLCSD
ZNT8       IFSILVLASTITILKDFSILLMEGVPKSLN--YSGVKELILAVDGVLSVHSLHIWSLTMN  (H301)
ZNT9       GVGTLLGMVSAFLIYTNTEALLGRSIQPEQ--VQRLTELLENDPSVRAIHDVKATDLGLG
ZNT10      LMVIIILSSAFPLIKETAAILLQMVPKGVN--MEELMSKLSAVPGISSVHEVHIWELVSG
YiiP       GIGIYILYSALRMGYEAVQSLLDRALPDEE--RQEIIDIVTSWPGVSGAHDLRTRQSGPT
            .                  ::                 :    .:   .. .
```

```
ZNT1            RIIATAHIKCED--PTSYMEVAKTIKDVFH-NHGIHATTIQPEFASVGSKSSVVPCELAC
ZNT2            QPVLSVHIAIAQ--NTDAQAVLKTASSRLQGKFHFHTVTIQIEDYSE-----DMKDCQAC
ZNT3            YHVASAHLAIDS--TADPEAVLAEASSRLYSRFGFSSCTLQVEQYQP-----EMAQCLRC
ZNT4            KSTAIVHIQLIPGSSSKWEEVQSKANHLLLNTFGMYRCTIQLQSYRQ----EVDRTCANC
ZNT5            IVAGTIHIQVTS--DVLEQRIVQQVTGILK-DAGVNNLTIQVEKEAYFQHMSGLSTGF--
ZNT6            SLAGSVHVRIRR--DANEQMVLAHVTNRLY--TLVSTLTVQIFKDDWIRP-------ALL
ZNT7            VYVGTLKLIVAP--DADARWILSQTHNIFT-QAGVRQLYVQIDFAAM-------------
ZNT8            QVILSAHVATAA--SRDSQVVRREIAKALSKSFTMHSLTIQMESPVD-----QDPDCLFC
ZNT9            KVRFKAEVDFDG-RVVTRSYLEKQDFDQMLQEIQEVKTPEELE------------TFMLK
ZNT10           KIIATLHIKYPK--DRGYQDASTKIREIFH-HAGIHNVTIQFENVDLKEPLEQKDLLLLC
YiiP            RFIQIHLEMEDSLPLVQAHMVADQVEQAILRRFPGSDVIIHQDPCSVVPREGKRSMLS--
                                            :           .
```

Figure S3 Amino acid alignment of ZnBPs: human ZIPs and *Bordetella bronchiseptica* ZIPB
Residues in red show Zn^{2+}-coordinating residues (PDB access code 5TSA, accessed July 2022). Red residues highlighted in blue coordinate Cd^{2+} (PDB 5TSA and 5TSB).

```
ZIP1       -------------------KLGALVLLLVLTLLCSLVPICVLRRPGANHEGSASRQKALS
ZIP2       -------------------KLGCLFALLALTLGCGLTPICFKWFQIDAARG--HHRLVLR
ZIP3       -------------------KILCMVGVFFFMLLGSLLPVKIIETDFE---KAHRSKKILS
ZIP4       --------------------------------------LLLLTCTGCRG----VTHYILQ
ZIP5       --------------------------------------LLLLRLLG-PR----LLRPLLG
ZIP6       --------------------------------------VILVPLMN-RV----FFKFLLS
ZIP7       --------------------------------------LFLIPVESNSP----RHRSLLQ
ZIP8       --------------------------------------LILTPLIK-KS----YFPKILT
ZIP9       --------------------------------------CYVAGIIP---LAVNFSEERLK
ZIP10      --------------------------------------VILVPIIN-QG----CFKFLLT
ZIP11      -------------MLQGHSSVFQALLGTFFTWGMTAAGAALVFVFS------SGQRRILD
ZIP12      --------------------------------------TALVLFHSCEE----NYRLILQ
ZIP13      --------------------------------------LLVIPLEMGTMLRSEAGAWRLK
ZIP14      --------------------------------------ASVVPFMK-KT----FYKRLLL
ZIPB       VVLLLVAGIVNALTGENRVHVGYAVLGGAAGFAATALGALMALGLR------AISARTQD
                                                   .

ZIP1       LVSCFAGGVFLATCLLDLLPDYLAAIDEALAALHV-------------------------
ZIP2       LLGCISAGVFLGAGFMHMTAEALEEIESQIQKFMVQNRSASERNSSGDAD----------
ZIP3       LCNTFGGGVFLATCFNALLPAVREKLQKVLSLGHI-------------------------
ZIP4       TFLSLAVGAVTGDAVLHLTPKVLG--------LHTH------------------------
ZIP5       FLGALAVGTLCGDALLHLLPHAQE--------GRH------------------------A
ZIP6       FLVALAVGTLSGDAFLHLLPHSHA--------SHHHSHSHEEPAMEMKRGPLFSHLSSQN
ZIP7       ILLSFASGGLLGDAFLHLIPHALE--------PHSHHTLEQPGHGHSHSG----------
ZIP8       FFVGLAIGTLFSNAIFQLIPEAFG--------FDP-------------------------
ZIP9       LVTVLGAGLLCGTALAVIVPEGVHALYEDILEGKHHQASETHNVIASDKAAEKSVVHEHE
ZIP10      FLVALAVGTMSGDALLHLLPHSQG--------GHDHSHQHAHGHGHSHGH--------ES
ZIP11      GSLGFAAGVMLAASYWSLLAPAVE--------MA------------------------TS
ZIP12      LFVGLAVGTLSGDALLHLIPQVLG--------LHKQEAP-------------------EF
ZIP13      QLLSFALGGLLGNVFLHLLPEAWA--------YTCSASPGGEGQS---------------
ZIP14      YFIALAIGTLYSNALFQLIPEAFG--------FNP-------------------------
ZIPB       AMLGFAAGMMLAASAFSLILPGLD--------AA------------------------GT
               :. * . .     :

ZIP1       -----TLQFPLQEFILAMGFFLVLVMEQITLAYKEQSGPS---------PLEETRALL--
ZIP2       ---SAHMEYPYGELIISLGFFFVFFLESLALQCCPGAAGG---------STVQDEE----
ZIP3       -----STDYPLAETILLLGFFMTVFLEQLILTFRKEKPSF---------IDLETFNAGSD
ZIP4       SEEGLSPQPTWRLLAMLAGLYAFFLFENLFNLLLPRDP-----------EDLEDGPCGHS
ZIP5       GPGGLPEKDLGPGLSVLGGLFLLFVLENMLGLLRHRGLRP-RCCRRKRRNL---------
ZIP6       IEESAYFDSTWKGLTALGGLYFMFLVEHVLTLIKQFKDKK--KKNQKKPENDDDVEIKKQ
ZIP7       -----QGPILSVGLWVLSGIVAFLVVEKFVRHVKGGHGHS-HGHGHAHS-----------
ZIP8       ----KVDSYVEKAVAVFGGFYLLFFFERMLKMLLKTYGQN--GHTHFGNDN---------
ZIP9       HSHDHTQLHAYIGVSLVLGFVFMLLVDQIGNSHVHSTDDP--------------------
ZIP10      NKFLEEYDAVLKGLVALGGIYLLFIIEHCIRMFKHYKQQR-GKQKWFMKQNTEESTIGRK
ZIP11      SGGFGAFAFFPVAVGFTLGAAFVYLADLLMPHLGAAEDPQ----------TTLALNFGST
ZIP12      GHFHESKGHIWKLMGLIGGIHGFFLIEKCFILLVSPNDKQ--------GLSLVNGHVGHS
ZIP13      -----LQQQQQLGLWVIAGILTFLALEKMFLDSKEEGTSQ-APNKDPTA-----------
ZIP14      ----LEDYYVSKSAVVFGGFYLFFFTEKILKILLKQKNEHHHGHSHYASESL--------
ZIPBB      IVGPGPAAAAVVALGLGLGVLLMLGLDYFTPHEHERTGHQ--------------------
                             *       :
```

```
ZIP1       ----------GTVN----------------------------------------------
ZIP2       -------------W----------------------------------------------
ZIP3       VGSDSEYESPFMGG----------------------------------------------
ZIP4       SHSHGGHSHG-VS----------------------LQLAPSELRQPKPPHEGSRA-----
ZIP5       --ETRNLDPENGSGMALQPLQAAPEPGAQGQREKN-------------------------
ZIP6       LSKYESQLSTNEE------KVDTDDRTEGYLRADSQEPSHFDSQQPA-----VLEEEEVM
ZIP7       ----------------------------------HTRGSHGHGRQERSTKEKQSSEEEEK
ZIP8       ----------------------------------------------------FGPQEKTH
ZIP9       ------------------------------------------------------------
ZIP10      LSDHKLNNTPDSDWLQLKPLAGTDDSVVSEDRLNETELTDLEGQQESPPKNYLCIEEEKI
ZIP11      LMKKKSDPEGPAL-----------------------------------------------
ZIP12      HHLALNSELSDQA----------------------GRGKSASTIQLKSPEDSQAA-----
ZIP13      ------------------------------------------------------------
ZIP14      ---------------------------------------------------PSKKDQEEG
ZIPBB      ------------------------------------------------------------

ZIP1       -----------------------------------------------GGPQHWHDGPGVP
ZIP2       -----------------------------------------------GGAHIFELHSHGH
ZIP3       -----------------------------------------------ARGHALYVEPHGH
ZIP4       ----------------------------------------------------DLVAEESP
ZIP5       ----------------------------------------------------SQHPPALA
ZIP6       IAHAHPQEVYNEYVPRGCKNKCHSHFHDTLGQSDDLIHHHHDYHHILHHHHHQNHHPHSH
ZIP7       ETRGVQKRRGGSTVPKD----------------------------------------GPV
ZIP8       QPKALPAINGVTCYANPAVTEANGHIHF-------------DN----VSVVSLQDGKKEP
ZIP9       ------------------------------------------------------------
ZIP10      IDHSHSDGLH-----TIHEHDLHAAAHNHHGENKTVLRKHNHQWH-------HKHSHHSH
ZIP11      ----------------LFPESELSIRIGRAGLLSDKSENGEAYQRKKAAATGLPEGPAVP
ZIP12      ---------------------------------------------------------EMP
ZIP13      ---AAAALNGGHCLA--------------------------------------------Q
ZIP14      VMEKLQNGDLDHMIPQHCSSELDGKAPM-------------VDEKVIVGSLSVQDLQASQ
ZIPBB      ------------------------------------------------------------

ZIP1       QASGA----PATPSALRACVLVFSLALHSVFEGLAVGLQRDRAR---------AMELCLA
ZIP2       LPSP-------SKGPLRALVLLLSLSFHSVFEGLAVGLQPTVAA---------TVQLCLA
ZIP3       GPSLSVQG-LSRASPVRLLSLAFALSAHSVFEGLALGLQEEGEK---------VVSLFVG
ZIP4       ELLNPEPRRLSPELRLLPYMITLGDAVHNFADGLAVGAAFASSW-------KTGLATSLA
ZIP5       PPGHQGHSHGHQGGTDITWMVLLGDGLHNLTDGLAIGAAFSDGF-------SSGLSTTLA
ZIP6       SQRYSREELKDAGVATLAWMVIMGDGLHNFSDGLAIGAAFTEGL-------SSGLSTSVA
ZIP7       RPQNAEEEKRGLDLRVSGYLNLAADLAHNFTDGLAIGASFRGGR-------GLGILTTMT
ZIP8       SSCTCLKGPKLSEIGTIAWMITLCDALHNFIDGLAIGASCTLSL-------LQGLSTSIA
ZIP9       ---------EAARSSNSKITTTLGLVVHAAADGVALGAAASTSQT------SVQLIVFVA
ZIP10      GPCHSGSDLKETGIANIAWMVIMGDGIHNFSDGLAIGAAFSAGL-------TGGISTSIA
ZIP11      VPSRGNLAQPGGSSWRRIALLILAITIHNVPEGLAVGVGFGAIEKTASATFESARNLAIG
ZIP12      IGSMTASNRKCKAISLLAIMILVGDSLHNFADGLAIGAAFSSSS-------ESGVTTTIA
ZIP13      PAAEPGLGAVVRSIKVSGYLNLLANTIDNFTHGLAVAASFLVSK-------KIGLLTTMA
ZIP14      SACYWLKGVRYSDIGTLAWMITLSDGLHNFIDGLAIGASFTVSV-------FQGISTSVA
ZIPBB      --------GPEAARVNRVWLFVLTIILHNLPEGMAIGVSFATGDL------RIGLPLTSA
                                      .   .*:*:.
ZIP1       LLLHKGILAVSLSLRLLQSHLRAQVVAGCGILFS-------CMTPLGIGLGAALAESAGP
ZIP2       VLAHKGLVVFGVGMRLVHLGTSSRWAVFSILLLA-------LMSPLGLAVGLAVTGGDSE
ZIP3       VAVHETLVAVALGISMARSAMPLRDAAKLAVTVS-------AMIPLGIGLGLGIESAQGV
ZIP4       VFCHELPHELGDFAALLHAGLSVRQALLLNLASA-------LTAFAGLYVALAV------
ZIP5       VFCHELPHELGDFAMLLQSGLSFRRLLLLSLVSG-------ALGLGGAVLGVGL------
ZIP6       VFCHELPHELGDFAVLLKAGMTVKQAVLYNALSA-------MLAYLGMATGIFI------
ZIP7       VLLHEVPHEVGDFAILVQSGCSKKQAMRLQLLTA-------VGALAGTACALLT--EGGA
ZIP8       ILCEEFPHELGDFVILLNAGMSTRQALLFNFLSA-------CSCYVGLAFGILV------
ZIP9       IMLHKAPAAFGLVSFLMHAGLERNRIRKHLLVFALAAPVMSMVTYLGLSKSSKE------
ZIP10      VFCHELPHELGDFAVLLKAGMTVKQAIVYNLLSA-------MMAYIGMLIGTAV------
ZIP11      IGIQNFPEGLAVSLPLRGAGFSTWRAFWYGQLSG-------MVEPLAGVFGAFA------
ZIP12      ILCHEIPHEMGDFAVLLSSGLSMKTAILMNFISS-------LTAFMGLYIGLSV------
ZIP13      ILLHEIPHEVGDFAILLRAGFDRWSAAKLQLSTA-------LGGLLGAGFAICTQSPKGV
ZIP14      ILCEEFPHELGDFVILLNAGMSIQQALFFNFLSA-------CCCYLGLAFGILA------
ZIPBB      IAIQDVPEGLAVALALRAVGLPIGRAVLVAVASG-------LMEPLGALVGVGI------
           :  ..   ..    :                .           .   .
```

```
ZIP1            LH-Q----LAQSVLEGMAAGTFLYITFLEILPQELASS-----------EQRILKVILLL
ZIP2            GGRG----LAQAVLEGVAAGTFLYVTFLEILPRELASP-----------EAPLAKWSCVA
ZIP3            PG-S----VASVLLQGLAGGTFLFITFLEILAKELEEK-----------SDRLLKVLFLV
ZIP4            ---G-VSEESEAWILAVATGLFLYVALCDMLPAMLKVR------DPRPWLLFLLHNVGLL
ZIP5            ---SLGPVPLTPWVFGVTAGVFLYVALVDMLPALLRPP------EPLPTPHVLLQGLGLL
ZIP6            ---GHYAENVSMWIFALTAGLFMYVALVDMVPEMLHND--ASDHGCSRWGYFFLQNAGML
ZIP7            VGSEIAGGAGPGWVLPFTAGGFIYVATVSVLPELLREA--------SPL-QSLLEVLGLL
ZIP8            ---G--NNFAPNIIFALAGGMFLYISLADMFPEMNDMLREKVTGRKTDFTFFMIQNAGML
ZIP9            ---ALSEVNATGVAMLFSAGTFLYVATVHVLPEVGGIGHSHKPDATGGRGLSRLEVAALV
ZIP10           ---GQYANNITLWIFAVTAGMFLYVALVDMLPEMLHGDGDNEEHGFCPVGQFILQNLGLL
ZIP11           ---VVLAEPILPYALAFAAGAMVYVVMDDIIPEAQISG------------NGKLASWASI
ZIP12           ---S-ADPCVQDWIFTVTAGMFLYLSLVEMLPEMTHVQ------TQRPWMMFLLQNFGLI
ZIP13           VGCSPAAEETAAWVLPFTSGGFLYIALVNVLPDLLEEE--------DPW-RSLQQLLLLC
ZIP14           ---G--SHFSANWIFALAGGMFLYISLADMFPEMNEVCQEDERKG-SILIPFIIQNLGLL
ZIPBB           ---SSGFALAYPISMGLAAGAMIFVVSHEVIPETHRNG------------HETTATVGLM
                                .: * ::::    :.
```

Figure S4 Amino acid alignment of ZnBPs: human metallothioneins
Colour codes for PDB structures: 2MHU, 1MHU, and 2F5H.

```
MT1A      MDPNCSCAT-GGSCTCTGSCKCKECKCTSCKKSCCSCCPMSCAKCAQGCICKGAS------EKCSCCA
MT1B      MDPNCSCTT-GGSCACAGSCKCKECKCTSCKKCCCSCCPVGCAKCAQGCVCKGSS------EKCRCCA
MT1E      MDPNCSCA-TGGSCTCAGSCKCKECKCTSCKKSCCSCCPVGCAKCAQGCVCKGAS------EKCSCCA
MT1F      MDPNCSCA-AGVSCTCAGSCKCKECKCTSCKKSCCSCCPVGCSKCAQGCVCKGAS------EKCSCCD
MT1G      MDPNCSCAAAGVSCTCASSCKCKECKCTSCKKSCCSCCPVGCAKCAQGCICKGAS------EKCSCCA
MT1H      MDPNCSCEA-GGSCACAGSCKCKKCKCTSCKKSCCSCCPLGCAKCAQGCICKGAS------EKCSCCA
MT1M      MDPNCSCTT-GVSCACTGSCTCKECKCTSCKKSCCSCCPVGCAKCAHGCVCKGTL------ENCSCCA
MT1X      MDPNCSCSPV-GSCACAGSCKCKECKCTSCKKSCCSCCPVGCAKCAQGCICKGTS------DKCSCCA
MT2       MDPNCSCA-AGDSCTCAGSCKCKECKCTSCKKSCCSCCPVGCAKCAQGCICKGAS------DKCSCCA
MT3       MDPETCPCPSGGSCTCADSCKCEGCKCTSCKKSCCSCCPAECEKCAKDCVCKGGEAAEAEAEKCSCCQ
MT4       MDPRECVCMSGGICMCGDNCKCTTCNCKTYWKSCCPCCPPGCAKCARGCICKGGS------DKCSCCP
```

Figure S5 Visual Basic script for extracting coloured sequences and flanking nucleotides from Excel cells
The script example below extracts red (RGB 255, 0, 0) codons and 9 flanking nts. The variables are highlighted in yellow.

```
Function ExtractRedCharactersWithFlankingCharacters(inputSequence As Range) As String
    ' Number of characters to include either side of the red characters
    Const NumberOfFlankingCharacters As Integer = 9
    ' Masking for readability
    Const NotSet As Integer = -1

    ' Extracted substring to return
    Dim result As String
    ' Loop variable
    Dim currentCharacter As Characters

    ' Starting and ending index of the red characters within the input string
    Dim RedPartStart As Integer
    Dim RedPartEnd As Integer

    ' Starting and ending index of the substring to extract
    Dim SubstringStart As Integer
    Dim SubstringEnd As Integer

    ' Initialise variables
    RedPartStart = NotSet
    RedPartEnd = NotSet

    ' For each character in the input
    For i = 1 To Len(inputSequence.Value)
        ' Get the character represented by the current index
        Set currentCharacter = inputSequence.Characters(i, 1)

        ' If the current character is red
        If currentCharacter.Font.Color = RGB(255, 0, 0) Then
            ' If this is the first red character encountered, set the index
            ' of the first red character to the current index
            If RedPartStart = NotSet Then
                RedPartStart = i
            End If

            ' If the index of the last of the red characters is less than
            ' the current index, set it to the current index
            If RedPartEnd < i Then
                RedPartEnd = i
            End If
        Else
            ' If we have already found a set of red characters, and this character
            ' is not a red character, we can exit early (we are assuming only one
            ' set of red characters)
            If RedPartEnd > NotSet Then
                Exit For
            End If
        End If

    Next
    ' If no red characters were found, exit with the empty string
    If RedPartStart = NotSet Then
        ExtractRedCharactersWithFlankingCharacters = ""
        Exit Function
    End If

    With Application.WorksheetFunction
        ' Set the starting index for the substring to the greater of 1 (start of string) and
        ' N characters before the first red character
        SubstringStart = .Max(RedPartStart - NumberOfFlankingCharacters, 1)
        ' Set the ending index for the substring to the lesser of the length of the string and
        ' N characters after the last red character
        SubstringEnd = .Min(RedPartEnd + NumberOfFlankingCharacters, Len(inputSequence.Value))
    End With

    ' Extract the substring from the input
    result = Mid(inputSequence.Value, SubstringStart, (SubstringEnd - SubstringStart) + 1)

    ExtractRedCharactersWithFlankingCharacters = result

End Function
```

Figure S6 Average PSI values of exons encoding Zn^{2+}- and Ca^{2+}-coordinating residues in ZnBPs and CaBPs in the indicated tissues

PSI values (%) were obtained from PSI tables of the VastDB (8). Error bars are SEMs. Number of exons in each protein group is as in Figure 2A.

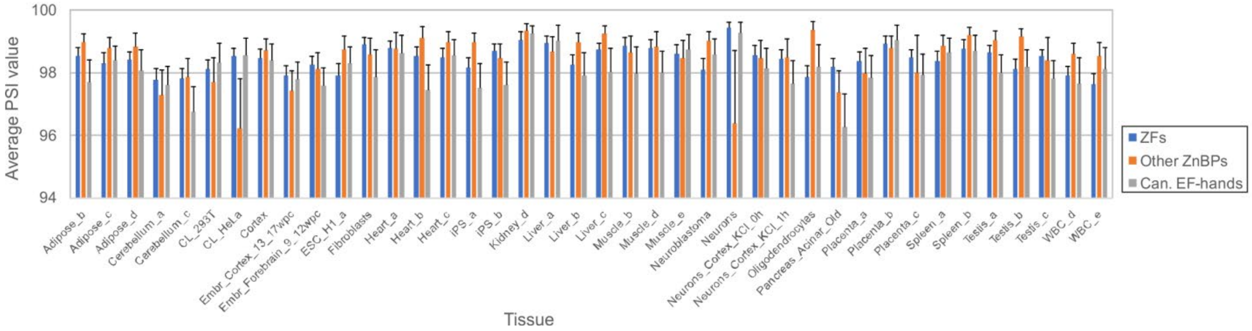

Figure S7 EST-derived exon inclusion data from the HEXEvent database

Exon inclusion types on the Y axis were defined previously (9). Briefly, constitutLevel = ([count]+[alt3]+[alt5]+[alt3+5])/([count]+[alt3]+[alt5]+[alt3+5]+[skip]);
3usageLevel is the usage level of major 3' splice site of the exon = ([count]+[alt5])/([count]+[alt3]+[alt5]+[alt3+5]);
5usageLevel is the usage level of major 5' splice site of the exon = ([count]+[alt3])/([count]+[alt3]+[alt5]+[alt3+5]).
Zn^{2+} and Ca^{2+} exon groups are as in Figure 2C. HEXEvent, control exons. Error bars are SEMs. *, $P<0.01$; **, $P<0.001$.

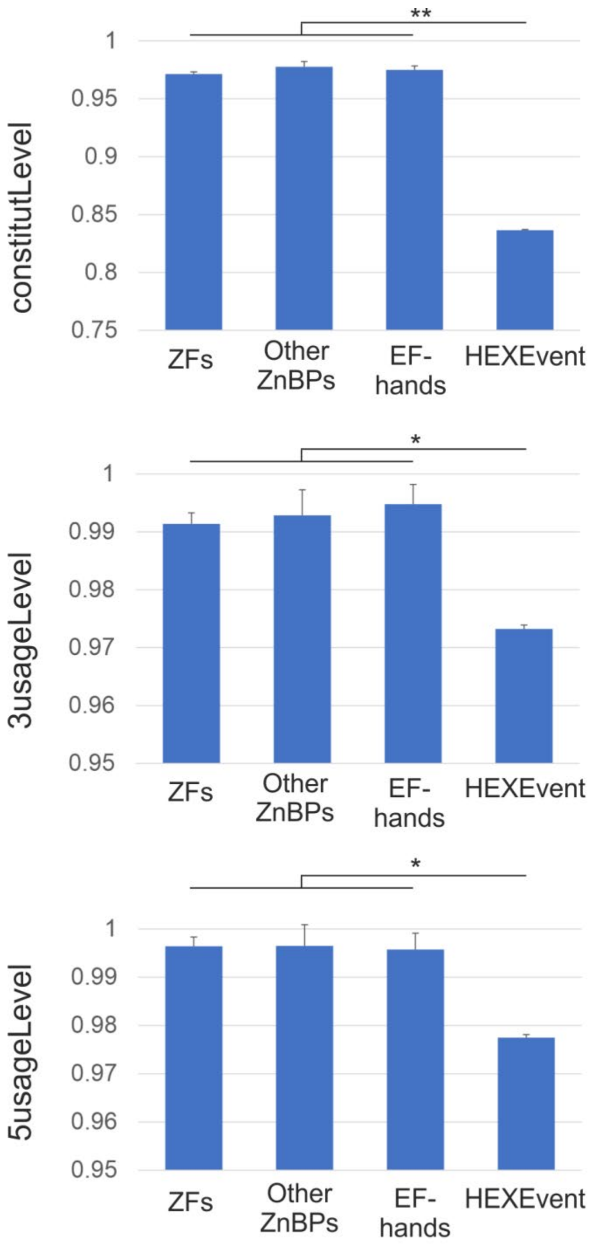

Figure S8 Nucleotide frequencies at position +1 of Zn^{2+} and Ca^{2+} exons
Controls are shown as horizontal red lines.

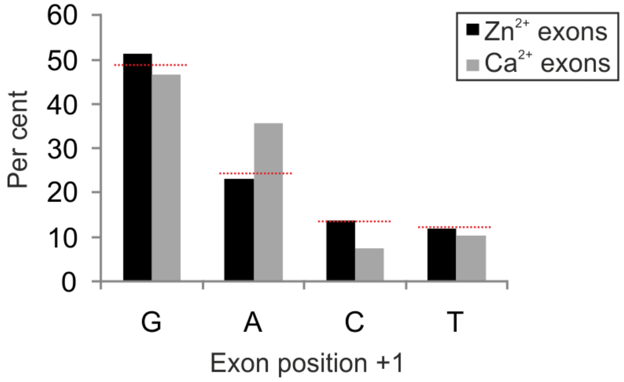

Figure S9 Distribution of PPT lengths in intronic ends preceding Zn^{2+} and Ca^{2+} exons
The PPT length was determined by the SVM-BPfinder (10).

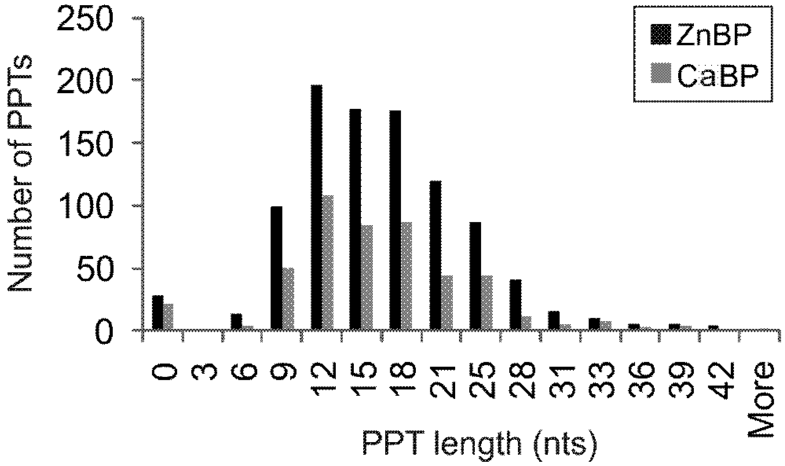

Figure S10 $(U)_4$ and $(C)_4$ motifs in PPTs of Zn^{2+} and Ca^{2+} exons
A, B, Distances between the first $(U)_4$ and $(C)_4$ motifs downstream of BPP-predicted BPs and 3'ss of Zn^{2+} and Ca^{2+} exons. **A,** $(U)_4$ motifs. **B,** $(C)_4$ motifs. **C,** Fractions of PPTs with at least one $(U)_4$ (*black columns*) or $(C)_4$ (*grey columns*) between BPP-predicted BPs and 3'ss in the indicated exon groups.

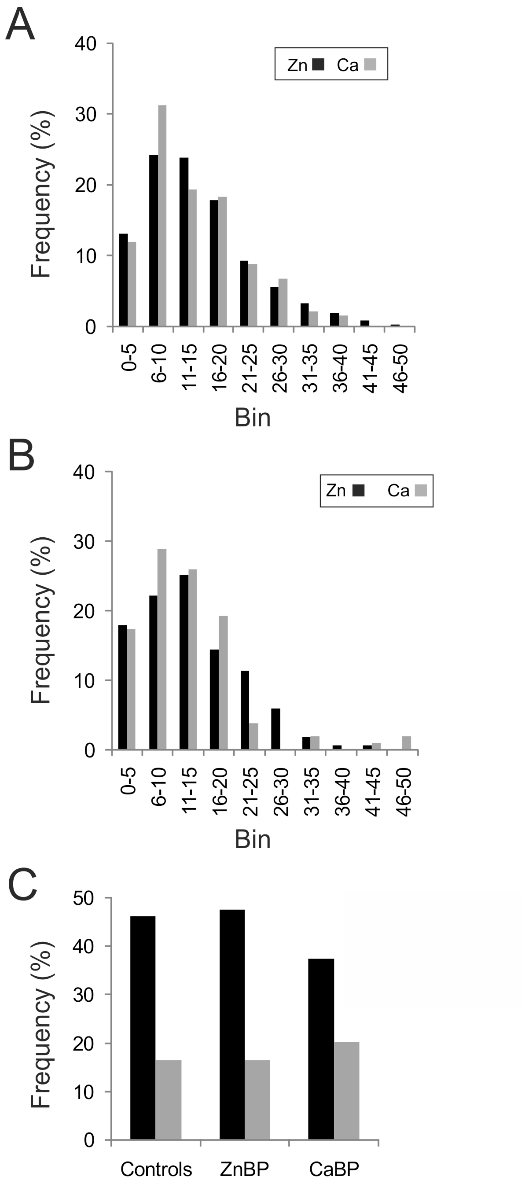

Figure S11 Minigene mutations

Mutations are in red. Exons are in upper case and bold, intronic ends are in lower case. This Figure complements Figures 4A, 4C and Supplementary Table S3.

***F8*-WT-ttt-t**
tcataatgttgatcacaacacaattttattttggttcttcactgtccctttaaaatag**ATTTGGCCAGGTGCGA**
F8-ccc-t
tcataatgttgatcacaacacaattttattttggttcttcactgtcccccaaaatag**ATTTGGCCAGGTGCGA**
F8-ttt-c
tcataatgttgatcacaacacaattttattttggttcttcactgtccctttaaaacag**ATTTGGCCAGGTGCGA**
F8-ccc-c
tcataatgttgatcacaacacaattttattttggttcttcactgtcccccaaaacag**ATTTGGCCAGGTGCGA**

UBE2F-WT-tttt-t
cagagtgtttgctttttgtttcctttttttttaatttaaaggatttttttgttttgttttgtgtttttgatag**ATGAGGGTTACTACCAGG**
UBE2F-cccc-t
cagagtgtttgctttttgtttcctttttttttaatttaaaggatttttttgttttgttttgtgtcccctgatag**ATGAGGGTTACTACCAGG**
UBE2F-tttt-c
cagagtgtttgctttttgtttcctttttttttaatttaaaggatttttttgttttgttttgtgtttttgacag**ATGAGGGTTACTACCAGG**
UBE2F-cccc-c
cagagtgtttgctttttgtttcctttttttttaatttaaaggatttttttgttttgttttgtgtcccctgacag**ATGAGGGTTACTACCAGG**

***HGD*-e10-WT-ttt-t**
atctttcctatgttttggaagtttctaaaagacttttgggttactgttttctag**GGGCCAATGGCTTGGCCAAT**
***HGD*-WT-ccc-t**
atctttcctatgttttggaagtttctaaaagacttttgggttactgtccccctag**GGGCCAATGGCTTGGCCAAT**
***HGD*-WT-ttt-c**
atctttcctatgttttggaagtttctaaaagacttttgggttactgttttccag**GGGCCAATGGCTTGGCCAAT**
***HGD*-WT-ccc-c**
atctttcctatgttttggaagtttctaaaagacttttgggttactgtccccccag**GGGCCAATGGCTTGGCCAAT**

Template F8-WT-ttt-t
tcataatgttgatcacaacacaattttattttggttcttcactgtccctttaaaatag**ATTTGGCCAGGTGCGA**
F8-TTT-T-CGC
tcataatgttgatcacaacacaattttattttggttcttcactgtccctttaaaatag**CGCTGGCCAGGTGCGA**
F8-TTT-T-GAA
tcataatgttgatcacaacacaattttattttggttcttcactgtccctttaaaatag**GAATGGCCAGGTGCGA**

Template F8-ttt-c
tcataatgttgatcacaacacaattttattttggttcttcactgtccctttaaaacag**ATTTGGCCAGGTGCGA**
F8-TTT-C-CGC
tcataatgttgatcacaacacaattttattttggttcttcactgtccctttaaaacag**CGCTGGCCAGGTGCGA**
F8-TTT-C-GAA
tcataatgttgatcacaacacaattttattttggttcttcactgtccctttaaaacag**GAATGGCCAGGTGCGA**

Template HGD-ccc-t
atctttcctatgttttggaagtttctaaaagacttttgggttactgtccccctag**GGGCCAATGGCTTGGCCAAT**
HGD-CCC-T-CGC
atctttcctatgttttggaagtttctaaaagacttttgggttactgtccccctag**CGCCCAATGGCTTGGCCAAT**
HGD-CCC-T-GAA
atctttcctatgttttggaagtttctaaaagacttttgggttactgtccccctag**GAACCAATGGCTTGGCCAAT**

Template HGD-ccc-c
atctttcctatgttttggaagtttctaaaagacttttgggttactgtccccccag**GGGCCAATGGCTTGGCCAAT**
HGD-CCC-C-CGC
atctttcctatgttttggaagtttctaaaagacttttgggttactgtccccccag**CGCCCAATGGCTTGGCCAAT**
HGD-CCC-C-GAA
atctttcctatgttttggaagtttctaaaagacttttgggttactgtccccccag**GAACCAATGGCTTGGCCAAT**

Figure S12 Association of TAG 3'ss and CAG 3'ss with nucleotides at exon positions +1 through +6

The proportions were computed for haplotypes at 195,421 intron-exon boundaries of the human reference sequence. This Figure complements Figure 4B.

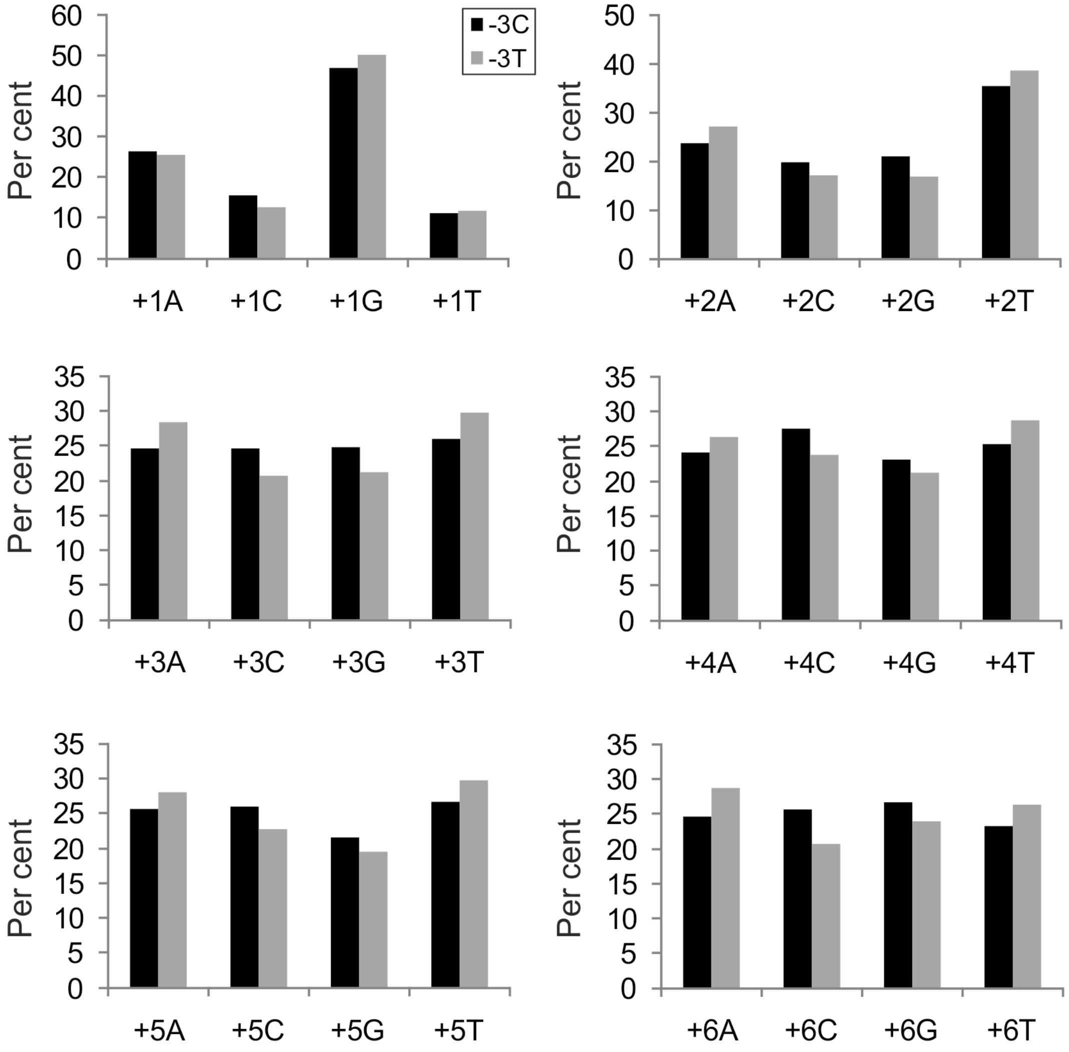

Figure S13 Comparison of two protein disorder scales showing inconsistencies for residues that bind tight metals (H and C)
DisProt was described in ref. (11) and TOP-IDP in ref. (12).

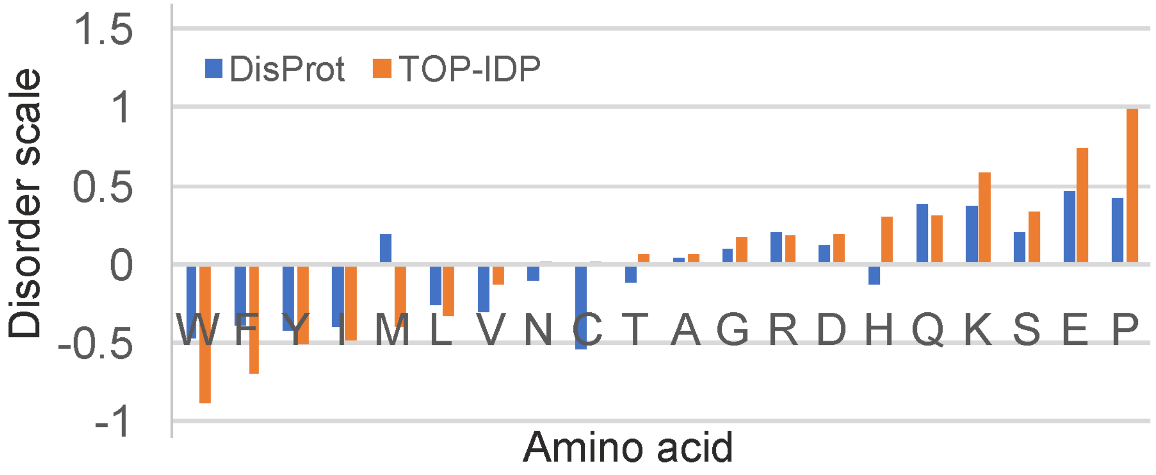

Figure S14 ESEf/ESSf ratios and molecular weight of amino acids
ESEf/ESSf scores are unweighted (A) or weighted (B) for residue frequencies at binding sites.

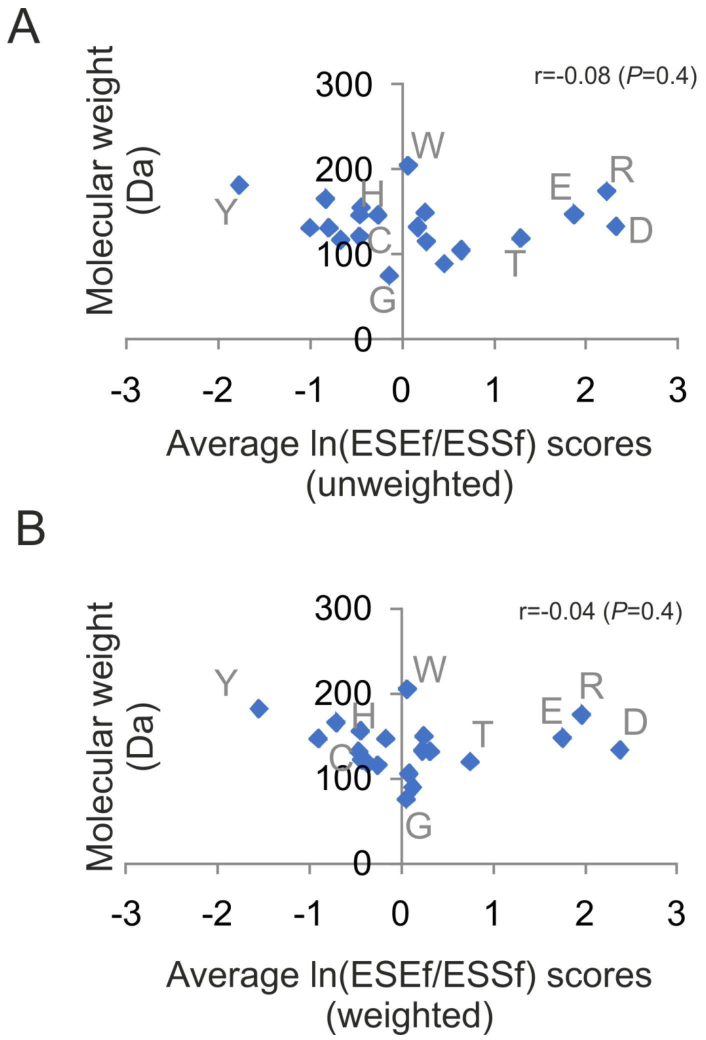

SUPPLEMENTARY TABLES

Table S1 Total number of Zn^{2+}-coordinating residues in stringent and extended datasets for the indicated species

Organism	Stringent dataset	Extended dataset
H. sapiens	3,251	3,854
M. musculus	456	456
R. norvegicus	114	114
Other mammals	41	41
Total	3,862	4,465

Table S2 Validation RT-PCR primers for alternative splicing of Zn^{2+}- or Ca^{2+} exons

Protein group	Gene symbol[1]	Forward primer (5'-3')	Reverse primer (5'-3')	Skipped/ spliced sizes (nts)	RT-PCR-detected alternative splicing[2]
**Human**					
ZnBP	*CA5A*	GCACCCGGCAGTCTCCTATTA	CGCCTATCACAGCCAAACCAT	244-363	
ZnBP	*KLK2*	TGGGACCTGGTTCTCTCCATC	GCCCAGGACCTTCACAACATC	257-417	
ZnBP	*NEIL3*	TGGTCTCCACCCAGCTGTTAA	ACAAACACTAGCAGGAGGAGAGT	258-425	ES
ZnBP	*ADH4*	TTAAATGCAAAGCAGCCATCGC	AGTGGACTCAGACAAAACTTGCA	162-304	
ZnBP	*CPA2*	GTCCGAGTTCCCTTCGTCAAC	TTGTGACTGGCAGGAGGAAGA	169-268	
ZnBP	*RNF144A*	CTGCTGACTCTGACATGCCC	AGCATCTGGGCAGCTAATTGC	257-403	ES, CSA
ZnBP	*ZNHIT3*	TCCTTCCACAAAACCATGGCG	CGAGTTTCAGGGTTGCACTGT	123-155	ES
ZnBP	*PHF5A*	GATCTTTTGCCGCAAGCA	ACTCATCACATATGCGCACCA	98-122	
ZnBP	*MYT1*	CTGTCCCACCCCTGGCTG	CTCCGGTGTGAAGCGTAGTTC	126-195	
ZnBP	*ZNF346*	AGAGCAGCAGAAGCAAAGACA	GGTTCTTGTGTTTGAAGCCGC	237-423	ES
ZnBP	*CDA*	TAGAAAATGCCTGCTACCCGC	CCATCCGGCTTGGTCATGTAC	144-202	
ZnBP	*DPYS*	GGTGACAGACCTGGAGCTGTA	TTCATGAGGAAGTCGGGTGTTG	242-432	ES
ZnBP	*ADAT2*	GCCAAAGAAGCCCTCGAAAAT	TGGATTTTCTTGTTTGTAGAAGGTCT	281-432	ES
ZnBP	*ZFAND1*	TTTTCTTCCATTTGTGTGTGATGA	TGCCACAAGTTCTCTCTCAGC	132-172	ES
ZnBP	*PHF7*	TATGCCCACACATCAGCAAAGC	CTGTGGCTGCAGCAGGTG	226-343	ES
ZnBP	*PHF7*	TGCCCCCATCTGTCTGTATGA	TTCTGACACCCTTGGACCTCC	249-371	ES
ZnBP	*DTX2*	CATCATCTGCATGGAGAAGCTGT	TGGGCGTTGTCTGGAAGGTAG	234-396	ES
ZnBP	*NAALADL1*	ACGACAACTGGATCCGGTACT	TGCGGAGTGTCTCACTGTAGT	260-342	
ZnBP	*SLC30A2*	TGACAGTCACTGTGACCCCAA	ATGATGTTCACAGCCACAGCG	249-396	
ZnBP	*SLC30A6*	ACACCACCCCATGTTATTGGT	AGGCAGGCCTAATCCAGTCAT	181-250	ES
ZnBP	*SLC39A5*	CCTTAGCGGTCTTCTGCCATG	GCCTCCATTCCTGTGGTTGG	243-434	
ZnBP	*SLC39A9*	GAACGACTGAAGCTGGTGACT	TGGACAACCAGACCCAGCG	170-368	ES
ZnBP	*SLC39A9*	TCCATGAACATGAGCACAGCC	TGAAGTAGATGCTGCTGCTCC	167-236	
CaBP	*CABP4*	CGAGGAGTTTGACACTGACCG	CACTTCTCGGAGCATCTCGTC	242-352	
CaBP	*CAPN12*	CATACCTCCACCCCCAGAGAG	CATTCAGTGCCAGCCTCAGC	136-199	
CaBP	*EFCAB1*	GCGGCCCGATTCTCTGAG	TCTTCCAAAGATCCTCGAAGAAACA	178-334	
CaBP	*EFCAB3*	AAGAAAAGAAGCTAAGTGCTTCAC	CCACTGCCTTGAGGAAGAGAT	105-249	
CaBP	*EFCAB5*	GCTCCAGTTTGTGCAACTCCT	TGGTCTTCATTGCCCCTGAGT	251-408	
CaBP	*MYL10*	CTCGGAAAAGAGCAGAAGGC	CAGCGCGGCAAAGGTGTC	155-257	ES
CaBP	*CABP4*	CTGATAGGCCCAAAGCTGAG	GCAGGGGCAACAGATATTCC	135-283	
CaBP	*ACTN1*	GGGCCTCCTTCAACCACTTTG	GCCAGGATCTTGAAGGAAGCC	236-317	ES
CaBP	*ACTN4*	GCAGCTGCTCACCACCATT	AAGTCGATGAAGGCTTGGAA	210-291	
CaBP	*DGKB*	CCTGCAAATACGTGTTCCCCA	GCAACATGCATCATCTGACTGA	135-185	ES
CaBP	*EFCAB3*	GTGCACTGTAGCTAAGCTGGG	CTGGGTAGGGCTGAAGTCTCT	174-246	ES
CaBP	*MYL10*	CTCGGAAAAGAGCAGAAGGC	TCTCCCCAAACATGGTCAGGA	278-354	
CaBP	*NECAB2*	CAGCTCTTCTTTGCAGATGGC	GGAGGCCAGGACATCCTCATA	127-153	ES
CaBP	*NUCB2*	AGGCTTAGAATGAGGGAACATGT	AGCCTCCAGTTGATCATGCTG	183-273	ES
CaBP	*SPATA21*	GTGGATGCACAGAGCCTGAAG	CAGCATCTCTACCAGCAGGGA	180-252	
**Rat**					
ZnBP	*Phf7*	AAATATGCCCACACGTCAGC	AAGCAGGCAGACACTCATTACA	219-336	ES
ZnBP	*Dtx2*	GACTGCATCATCTGTATGGAGAA	GTAGCACTGCCGGGGAAAG	222-384	
ZnBP	*Slc39a9*	CTGAAGCTGGTGACTGTGTTG	CCCAGTGTGGTGGTGATTT	152-353	
CaBP	*Nucb2*	GTATAACCCGCAGAATGCAGA	CGCTGCAGCTCCTCCTTC	210-300	
ZnBP	*Zfand1*	TTCCTTCCATTTGTATGTGACG	GTAAGGACAGATCACTGCCACA	149-189	ES

[1]If available, hyperlinks in the Gene symbol column show VastDB data for comparison. [2], ES, exon skipping; CSA, cryptic splice-site activation.

.

Table S3 Mutagenic primers for *F8, UBE2F* and *HGD* minigenes

**Primer designation**	**Primer sequence (5' to 3')**
**Mutations at PPT and position -3 relative to 3'ss**	
F8-CCC-T	GTTCTTCACTGTCCCCCCAAAATAGATTTGGC
F8-TTT-C	TGTCCCTTTAAAACAGATTTGGCCAG
F8-CCC-C	GTTCTTCACTGTCCCCCCAAAACAGATTTGGCCAGGTGC
UBE2F-CCCC-T	GGATTTTTTTGTTTTGTTTTGTGTCCCCTGATAGATGAGGGTTAC
UBE2F-TTTT-C	GTTTTGTGTTTTTTGACAGATGAGGGTTAC
UBE2F-CCCC-C	GATTTTTTTGTTTTGTTTTGTGTCCCCTGACAGATGAGGGTTACTACC
HGD-WT-CCC-T	CTTTTGGGTTACTGTCCCCTAGGGGCCAATGGCT
HGD-WT-TTT-C	GTTACTGTTTTCCAGGGGCCAATG
HGD-WT-CCC-C	GACTTTTGGGTTACTGTCCCCCAGGGGCCAATGGCTTG
**Mutations at exon positions +1 through +3**	
F8-TTT-T-CGC	CCCTTTAAAATAGCGCTGGCCAGGTGCGA
F8-TTT-T-GAA	CCCTTTAAAATAGGAATGGCCAGGTGCGA
F8-TTT-C-CGC	CCCTTTAAAACAGCGCTGGCCAGGTGCGA
F8-TTT-C-GAA	CCCTTTAAAACAGGAATGGCCAGGTGCGA
HGD-CCC-T-CGC	TACTGTCCCCTAGCGCCCAATGGCTTGGC
HGD-CCC-T-GAA	TACTGTCCCCTAGGAACCAATGGCTTGGC
HGD-CCC-C-CGC	TACTGTCCCCCAGCGCCCAATGGCTTGGCC
HGD-CCC-C-GAA	TACTGTCCCCCAGGAACCAATGGCTTGGCC

Table S4 Minigene cloning primers

Primer designation	Sequence 5'-3'
**Disease-associated**	
ABCA4F	ACCACTCGAGTCTTTGAATCTTTACACTCAGGGC
ABCA4R	ACCATCTAGATGCTTAAAAGGTTCCGTGA
AMELX-F	ACCACTCGAGTCATAATTTTGTGTTTGTTTTGCTC
AMELX-R	ACCATCTAGAGCAGTGTTTTCTTTGCCATTATG
ANO3-F	ACCCCTCGAGTGGAAGCAGTTAACCACCATTAG
ANO3-R	CAAGTCAAAAGCAAAACCGTAGA
ARX-F	ACCACTCGAGTCTGGTAATGTGCTTCGTGT
ARX-R	ACCATCTAGATGGAACTGAGCGGATTTGTC
CFTR-E11F	ACCACTCGAGTGGACCTATGGATGATCTACACA
CFTR-E11R	ACCATCTAGAGGTGTGTACATTGGAGTGGC
CFTR-E4F	ACCACTCGAGAAGGGAAATGCTTTAGAAACTGTT
CFTR-E4R	ACCATCTAGATGTAGGCTGTGTGAGTCATCT
LAMB3-F	ACCACTCGAGCCACATTCACGCATCCTTGG
LAMB3-R	ACCATCTAGACCAGACCTCAACCATCCCAT
OTC-F	ACCACTCGAGCTGAAAGGTCTACTGTTCCACT
OTC-R	ACCATCTAGAGCGGATCAAGGGTGGTAAGA
PKHD1-F	ACCACTCGAGTTACTAGCTGTGGGGTTATTGAA
PKHD1R	TGCTTATGGGGCTTTAGTGC
SGCE-F	ACCACTCGAGCCAGTGCTTAATGGCCTTCT
SGCE-R	ACCATCTAGACAGGCTCTCACCCTATCAGC
CFTR-E3F	ACCACTCGAGTGAAATCAATTCCCTGCACA
CFTR-E3R	ACCATCTAGACAGCAAAATAAAGGCACCA
COL4A3-F	ACCACTCGAGACGTCCTTACTATTGCTGTCAATG
COL4A3-R	ACCATCTAGATAGAGCCCCGAGACTGACA
CRB2-Fnew	ACCACTCGAGCTGCCCTCTCTCTTGGACT
CRB2-Rnew	ACCATCTAGACCCAAAGGTCAGAGTCATCC
FRMD7-F	ACCACTCGAGCAAGCCCTTAAACCTGAGA
FRMD7-R	ACCATCTAGATTCGCGTGGTCTGAAAATC
KIF5A-F	ACCACTCGAGCCTGGGCAGCATAGTAAAG
KIF5A-R	ACCATCTAGACGGCAGGGACTATTTGTGTT
NIPBL-F	ACCACTCGAGCGGATAAAGCACACAAAGGA
NIPBL-R	ACCATCTAGATGGGTAAAATTAGGGCGAAG
PARNFnew	ACCACTCGAGAAGTTTAGCATTTTCAGTGTTAATTTG
PARNRnew	ACCATCTAGAGCCCCAAAGCCCAAAGTTAA
TGFBI-F1	ACCACTCGAGTATTGGGGCCTCTCTAACCG
TGFBI-R1	ACCATCTAGATACTGCGCAGAGAGTGAGAG
SMN1-F	ACCACTCGAGGGTGTCAAGCTCCAGGTCTC
SMN1-R	ACCATCTAGAAAAGTCTGCTGGTCTGCC
**Zn^{2+}/Ca^{2+} exons**	
DTNAf	ACCACTCGAGTTTCATTTCATTTCCCCACAG
DTNAr	ACCATCTAGAGCTACACTGGGTTCCAACAGA
EPS15f	ACCACTCGAGCCTATGGATAAGTCCTAACTGACCT
EPS15r	ACCATCTAGAGCTTCAGGTAACCCATTCTCA
GUCA1Cf	ACCACTCGAGCACTAGGGAAAAGTTTTATGCTGAA
GUCA1Cr	ACCATCTAGATTTGTGAATGTCAGGGTGGA
HERC2f	ACCACTCGAGTATGACATGGCTAGGTGCAGTT
HERC2r	ACCATCTAGATGGAAAAACAAGCAAACAAGC
KDM5Df	ACCACTCGAGAAACCTGCGATAAGGTGTCA
KDM5Dr	CCTTACATGCTTCATGCCATT
KMT2C-f	ACCACTCGAGCCAGCATTATCATTGGCATTAGGA
KMT2C-r	ACCATCTAGACGACCAGAGCAATTTAAATGATT
MBNL2f	ACCACTCGAGGCTCCGCATAATCAATCTTT
MBNL2r	ACCATCTAGACCTGCACCTGAGGGATTTACT
PPP3R1f	ACCACTCGAGGAAGGAGACATCCTCATAGTCA
PPP3R1r	ACCATCTAGATGTAATTTGAGAGGCTACCAACAA

REPS2f	ACCACTCGAGCTGATGCTGATCCCATCAATC
REPS2r	ACCATCTAGATTACACAGCCTGGAGGAAAAGT
SLC25A24f	ACCACTCGAGTCAATGCATCATTTTTCTGATTG
SLC25A24r	ACCATCTAGACATTTCAAAGTAATGAAAAAGAAAAGC
CALM1	ACCACTCGAGAGGCCAGTCCAACAGGACTAT
CALM1	GTGATCCCATGCAGAAAACAT
CAPSf	ACCACTCGAGTCCCCTCTCACCTTCCTGA
CAPSr	ACCATCTAGAGGGACAGGAGGTGGTGACT
CHD4f	ACCACTCGAGTATTTGTGATGGGATGAGGTG
CHD4r	ACCATCTAGACCGCAGTCTGAAGCCAGT
CRACR2A	ACCACTCGAGCTGGCAGAAGGCTCTGCAGTC
CRACR2A	AGGGAGCTTGGTAATGGCAGT
CXXC1f	ACCACTCGAGATAGAAGATCCCACGGGAAGA
CXXC1r	ACCATCTAGAGTCATGCCCCATTCATCTG
NOX5f	ACCACTCGAGCGGAACAGGTCTTCCATTAGAG
NOX5r	ACCATCTAGAGGAGAGAGATGTGAGGAAAGGA
SRIf	ACCACTCGAGAAGTTATCTTTTCCCCACAGTAAAAA
SRIr	ACCATCTAGAAATCAACTTACTTCTACCAATGAATG
UHRF1f	ACCACTCGAGCTGTTCCGTTTCATGTTGCTT
UHRF1r	ACCATCTAGACCCCTCTGGGGATTTGTAG
ZNF410f	ACCACTCGAGCCCAAGCTGATCTCAAACTCCT
ZNF410r	ACCATCTAGAGGCTCCTGAAAACAGAAATCAACA
FBXL19f	ACCACTCGAGAGGGAGACAGGCCTTGAGTAG
FBXL19r	ACCATCTAGAGTCAGATCCTTCTGTGCACCT
TRIM28f	ACCACTCGAGTTCTCAGCTATGTTGGGGCA
TRIM28r	ACCATCTAGACTAGGTAGGGTCTTGCTGCC
TRIM23f	ACCACTCGAGTGCCCCAACACTTAGTATTTCA
TRIM23r	ACCATCTAGAGCTTTCTGAAACTTTCTGGGAAT
GCAf	ACCACTCGAGTTCAGTGCCTGTAGTAAACTAGTGA
GCAr	ACCATCTAGAATGTATGCATGTATGGAAGATCA
ITSN1f	ACCACTCGAGGCCAGTATTTACAGGCTATTCC
ITSN1r	ACCATCTAGACTGCATCTCTGCTTTCATTTTG

Mutagenic primers are available on request.

Supplementary Table S5 Compilation of published disease-associated T>C and C>T variants at position -3 relative to 3'ss

Gene symbol	Mutation/variant	Reference	PMID	Reported splicing defect for endo/ exogenous transcripts	Associated phenotype	Exon sequence	End sequence of preceding intron	Tested in this study
	**-3T>C**							
ABCA4	c.5899-3T>C	Nassisi M, et al. Expanding the mutation spectrum in *ABCA4*: sixty novel disease causing variants and their associated phenotype in a large French Stargardt cohort. Int. J. Mol. Sci. 2018;19(8):2196.	30060493	Not tested	Stargardt disease	TGCTTTGGCCTCCTGGGAGTGAATGGTGCCGGCAAAACAACCACATTCAAGATGCTCACTGGGGACACCACAGTGACCTCAGGGGATGCCACCGTAGCAGGCAAGAG	gcctgacattttagaccttcttgatctctagggccaggctagctctgtgttttctcc(t>c)agTGCT…	Yes
ABCC8 (SUR1)	c.2117-3T>C	Song J, et al. *KCNJ11*, *ABCC8* and *TCF7L2* polymorphisms and the response to sulfonylurea treatment in patients with type 2 diabetes: a bioinformatics assessment. BMC Med. Genet. 2017;18:1-7.	28587604	Not tested	Type 2 diabetes	GCCAGCTGACTATGATCGTGGGGCAGGTGGGCTGCGGCAAGTCCTCGCTCCTTCTAGCCGCACTGGGGGAGATGCAGAAGGTCTCAGGGGCTGTCTTCTGGAGCAG	ggtaatggttgttcagactcccccggccccactcacatctgccaccctccctccctg(t>c)agGCCA…	No
ADCY3	c.2578-3T>C	Toumba M, et al. Molecular modelling of novel ADCY3 variant predicts a molecular target for tackling obesity. Int. J. Mol. Med. 2022;49(1):1.	34821371	Not tested	Obesity	GTAGAAAAACTGGCACGGACACTTTTCTTGTGGAAGATTGAGGTCCACGACCAGAAGGAACGTGTCTATGAGATGCGACGCTGGAACGAGGCCTTGGTCACCAACATGTTGCCTGAGCACGTGGCACGCCATTTCCTGGGGTCCAAGAAGAGAGATGAG	ttcatccttctatccagtggccagcttagagcacatgcctcgccttgtctgtgtctg(t>c)agGTAG…	No
AMELX	c.103-3T>C	Leban T, et al. An Intron c. 103-3T> C Variant of the AMELX Gene Causes Combined Hypomineralized and Hypoplastic Type of Amelogenesis Imperfecta: Case Series and Review of the Literature. Genes. 2022;13(7):1272.	35886055	Not tested	Amelogenesis imperfecta	AACTCACATTCTCAGGCTATCAATGTTGACAGGACTGCATTA	cacagtgcctgtcacacaggaagcacccaacaattttttaccttcttctttcttttg(t>c)agAACT…	Yes
ANO3	c.1531-3T>C	Jiang LT, et al. The expanding clinical and genetic spectrum of ANO3 dystonia. Neurosci. Letters 2021;746:135590.	33388357	Not tested	Dystonia	GAAACACTTCGTCCCCAGTTTGAAGCCAAGTATTACAAGATGGAGATTGTAAATCCCATCACGGGAAAACCTGAACCACATCAGCCTTCCTCAGACAAAGTCACTCGTCTTCTTGTTTCTGTCTCAGGAATATTCTTCATG	gggggtatgtttttttagtttatggctttgatatttagataactttcgtttctctca(t>c)agGAAA…	Yes
ARX	c.1074-3T>C	de Souza Gestinari-Duarte R, et al. Mutational screening of ARX gene in Brazilian males with mental retardation of unknown etiology. J. Hum. Genet 2006;51(8):737-40.	16845484	Not tested	Mental retardation	GGAGGAACTGGCCATGAGGCTGGACTTGACCGAGGCCCGAGTCCAG	gctgagagggcattgctggggcctgcagtgacctcctgtctgtgtgttgctttctta(t>c)agGGAG…	Yes
BRCA2	c.6938-3T>C	Brandao RD, et al. Characterisation of unclassified variants in the *BRCA1/2* genes with a putative effect on splicing. Breast Cancer Res. Treat. 2011;129:971-82.	21638052	N.T./No splicing defect	Breast cancer	GCACAATAAAAGATCGAAGATTGTTTATGCATCATGTTTCTTTAGAGCCGATTACCTGTGTACCCTTTCG	gtaacatggatattctcttagattttaactaatatgtaatataaaataattgtttcc(t>c)agGCAC…	No
CFTR	c.1585-3T>C	Raynal C, et al. A classification model relative to splicing for variants of unknown clinical significance: application to the CFTR gene. Hum, Mutat. 2013;34(5):774-84.	23381846	N.A./No splicing defect found	Cystic fibrosis	GACATCTCCAAGTTTGCAGAGAAAGACAATATAGTTCTTGGAGAAGGTGGAATCACACTGAGTGGAGGTCAACGAGCAAGAATTTCTTTAGCAAG	tttcaaattcagattgagcatactaaaagtgactctctaattttctatttttggtaa(t>c)agGACA…	Yes
CFTR	c.274-3T>C[5]	Kılınç MO, et al. Highest heterogeneity for cystic fibrosis: 36 mutations account for 75% of all CF chromosomes in Turkish patients. Am. J. Med. Genet. 2002;113(3):250-7. Alibakhshi R, Mohammadi A, Khamooshian S, Kazeminia M, Moradi K. CFTR gene mutation spectrum among 735 Iranian patients with cystic fibrosis: A	12439892 34525262	Not tested	Cystic fibrosis	GAAGTCACCAAAGCAGTACAGCCTCTCTTACTGGGAAGAATCATAGCTTCCTATGACCCGGATAACAAGGAGGAACGCTCTATCGCGATTTATCTAGGCATAGGCTTATGCCTTCTCTTTATTGTGAGGACACTGCTCCTACACCCAGCCATTTTTGGCCTTCATCACATTGGAATGCAGATGAGAATAGCTATGTTTAGTTTGATTTATAAGAAG	ctcagggtattttatgagaaataaatgaaatttaatttctctgtttttcccctttttg(t>c)agGAAG…	Yes

		comprehensive systematic review. Pediat. Pulmonol. 2021;56(12):3644-56.						
FLT3	c.1310-3T>C	Kassem NM, et al. Targeted next generation sequencing provides insight for the genetic alterations in liquid biopsy of Egyptian brain tumor patients. Egypt. J. Med. Hum. Genet. 2022;23(1):23.	https://doi.org/10.1186/s43042-022-00214-y	Not tested	Glioblastoma	GGAAACCTCAAGTGCTCGCAGAAGCATCGGCAAGTCAGGCGTCCTGTTTCTCGGATGGATACCCATTACCATCTTGGACCTGGAAGAAGTGTTCAGACAAGTCTCCCAA	ttataggagtgaaccactatgcccagccagtgagcttatttcacacgttcttttcta(t>c)agGGAA…	No
LAMB3	c.565-3T>C	Pasmooij AM, et al. Revertant mosaicism in junctional epidermolysis bullosa due to multiple correcting second-site mutations in *LAMB3*. J. Clin. Invest. 2007;117(5):1240-8.	17476356	Not tested	Epidermolysis bullosa	GTCCAACTTAACCTTATGGATTTAGTGTCTGGGATTCCAGCAACTCAAAGTCAAAAAATTCAAG	tgaactcggggcacactatttgtctcttcttcagcgcttctctttcccttccctctg(t>c)agGTCC…	Yes
OTC	c.867-3T>C[1]	Lee JH, et al. *OTC* gene in ornithine transcarbamylase deficiency: clinical course and mutational spectrum in seven Korean patients. Pediat. Neurol. 2014;51(3):354-9.	25011434	Not tested	Ornithine transcarbamylase deficiency	ACTGCTAAAGTTGCTGCCTCTGACTGGACATTTTTACACTGCTTGCCCAGAAAGCCAGAAGAAGTGGATGATGAAGTCTTTTATTCTCCTCGATCACTAGTGTTCCCAGAGGCAGAAAACAGAAAGTGGACAATCATG	aaaacaaatatgactgccacatataatagtcaaaaagtggtcttatccccatctctt(t>c)agACTG…	Yes
PAH	c.61-3T>C	Liu N, et al. Prenatal diagnosis of Chinese families with phenylketonuria. Genet Mol Res. 2015 Nov 19;14(4):14615-28.	26600521	Not tested	Phenylketonuria	GAAACAAGCTATATTGAAGACAACTGCAATCAAAATGGTGCCATATCACTGATCTTCTCACTCAAAGAAGAAGTTGGTGCATTGGCCAAAGTATTGCGCTTATTTGAG	aacaggaatgaattgctaaactgtggaaaatgttttaaacaaatgcatcttatcctg(t>c)agGAAA…	No
PKHD1	c.2141-3T>C	Li H, et al. A Potential Therapy Using Antisense Oligonucleotides to Treat Autosomal Recessive Polycystic Kidney Disease. J. Clin. Med. 2023;12(4):1428.	36835961	N.A./upstream cryptic 3'ss activation	Polycystic kidney disease	TTTCTCAAGCTGATTCTGGAACGGCTCGCCCAGGGGGCAATCTGGTGGAATCAGTCTCTGTGGTGGGATCCCCTCCGGTCTACAGTGTCACCTCCTGGCTGGCGGGGTGTGGCACGGAGCTCCCGCTCATCACTGCACG	tccacacagcaagtctaccatcttaagtcacttgatagtaattggatcactggtctc(t>c)agTTTC…	Yes
PTCH1	c.2704-3T>C<br>(rs1208489068)	Abe S, et al. Coincident two mutations and one single nucleotide polymorphism of the *PTCH1* gene in a family with naevoid basal cell carcinoma syndrome. Acta Dermatovener. 2008;88(6):635-6.	19002359	Not tested	Gorlin syndrome	TTGACTAAACAGCGTCTGGTGGATGCAGATGGCATCATTAATCCCAGCGCTTTCTACATCTACCTGACGGCTTGGGTCAGCAACGACCCCGTCGCGTATGCTGCCTCCCAGGCCAACATCCGGCCACACCGACCAGAATGGGTCCACGACAAAGCCGACTACATGCCTGAAACAAGGCTGAGAA	cagaagtgtgtttaccccaaccccattctcaaaggcctctgttcttcccgtttttg(t>c)agTTGA...	No
RAB3GAP2	c.2578-3T>C	Chesneau B, et al. First evidence of *SOX2* mutations in Peters' anomaly: Lessons from molecular screening of 95 patients. Clin. Genet. 2022;101(5-6):494-506.	35170016	Not tested	Peter's anomaly	TTTTCCCAAACAGTTTTGGGTGCTGATTCAGAGGCCCTCACTGATTCCTGGGAGGCACTTTCTCTTGACACTGAGTACTGGAAACTCCTTCTGAAACAGCTGGAGGATTGTCTCATACTTCAGACTCTGCTTCACAGCAAAGGGAACACTCAGACCTCCAAAGTGTCATCACTGCAGGCTGAGCCACTTCCAAGGCTTTCTGTTAAAAAGTTATTAGAAGGAGGAAAAG	ctcattcaagttaatattgattcagaatctttggcaaagaatcaccagtgcattttttat(t>c)agTTTT…	No
SCN1A	c.4477-3T>C	Hata Y, et al. Two autopsy cases of sudden unexpected death from Dravet syndrome with novel de novo *SCN1A* variants. Brain Dev. 2020;42(2):171-8.	31677916	Not tested/2-bp insertion (AC) between exons 23 and 24, resulting in protein truncation	Dravet syndrome	TTTGGAGGTCAAGACATCTTTATGACAGAAGAACAGAAGAAATACTATAATGCAATGAAAAAATTAGGATCGAAAAAACCGCAAAAGCCTATACCTCGACCAGGA	cattgaaaaaaatgtacaaaaggacacagttttaaccagtttgatttttcttttcta(t>c)acTTTG…	No
SGCE	c.391-3T>C	Rachad L, et al. Screening for *SGCE* mutations in Moroccan sporadic patients with Myoclonus-Dystonia syndrome. Neurosci. Lett. 2019;703:1-4.	30849405	Not tested	Benign myoclonus-dystonia syndrome	ATAACTGCCT ACAACAGGCGCACCTTTGAGACTGCAAGGCATAATTTGATAATTAATATAATGTCTGCAGAAG	atgaatttcattttacaaagaacatttcacttctttaatatggttttccttttataa(t>c)agATAA…	Yes
	**-3C>T**							
ATM	c.3994-3C>T	Bueno-Martínez E, et al. Minigene-based splicing analysis and ACMG/AMP-based tentative classification of 56 *ATM* variants. J. Pathol. 2022;258(1):83-101.	35716007	Increased exon inclusion (not shown)	Ataxia telangiectasia	ATTGATCACTTATTCATTAGTAATTTACCAGAGATTGTGGTGGAGTTATTGATGACGTTACATGAGCCAGCAAATTCTAGTGCCAGTCAGAGCACTGACCTCTGTGACTTTTCAGG	gctgtcttgacgttcacagatataaaatattaaatatattttaattttgtgcccttg(c>t)agATTG…	No
CFTR	c.165-3C>T	Bienvenu T, et al. Unexpected inactivation of acceptor consensus splice sequence by a -3C to T transition in intron 2 of the *CFTR* gene. Human genetics. 1994;94:65-8.<br>Joynt AT, et al. Evaluation of both exonic and intronic variants for effects on RNA	7518409<br>33085659	Exon 3 skipping	Cystic fibrosis	AGAATGGGATAGAGAGCTGGCTTCAAAGAAAAATCCTAAACTCATTAATGCCCTTCGGCGATGTTTTTTCTGGAGATTTATGTTCTATGGAATCTTTTTATATTTAGGG	taggacaactaaaatatttgcacatgcaacttattggtcccactttttattcttttg(c>t)agAGAA…	Yes

		splicing allows for accurate assessment of the effectiveness of precision therapies. PLoS Genet. 2020;16(10):e1009100.						
COL4A3	c.4028-3C>T	Mohamed M, et al. Pseudodominant Alport syndrome caused by pathogenic homozygous and compound heterozygous *COL4A3* splicing variants. Ann. Hum. Genet. 2022;86(3):145-52.	34888854	Exon 46 skipping/Not tested	Alport syndrome	GTGTACGTGGAGACCCTGGCACACTTAAGATTATCTCCCTTCCAGGAAGCCCAGGGCCACCTGGCACACCTGGAGAACCAGGGATGCAGGGAGAACCTGGGCCACCAGGGCCACCTGGAAACCTAG	aatgaagaaaggtaacacatccgtgaggccatcatcttcttcttatgtttatgtcaa(c>t)agGTGT…	Yes
CRB2	c.941-3C>T	Simaab A, et al. Exome Sequencing Revealed a Novel Splice Site Variant in the *CRB2* Gene Underlying Nephrotic Syndrome. Medicina. 2022 Dec 4;58(12):1784.	36556986	Not tested/cr3'ss 189 nts downstream suggested	Nephrotic syndrome	GAGCCGACTGCGGTGTGGAGGTGGACGAGTGTGCCTCACGGCCATGCCTCAACGGAGGCCACTGCCAGGACCTGCCCAATGGCTTCCAGTGTCACTGCCCAGATGGCTACGCAG	ctctcagcccctgcctggctctgagggtgcaggtgggacccacagctgggcctctta(c>t)agGAGC…	Yes
FAS	c.551-3C>T	Corrionero A, et al. Strict 3' splice site sequence requirement for U2 snRNP recruitment after U2AF binding underlie genetic defect leading to autoimmune disease.	21233219	Exon 6 skipping by minigene assays. The -3C>G mutation reduced U2 snRNP interaction with the 3'ss	Autoimmune lymphoproliferative syndrome	GATCCAGATCTAACTTGGGGTGGCTTTGTCTTCTTCTTTTGCCAATTCCACTAATTGTTTGGG	tcatataaaatgtccaatgttccaaccta(c>t)agGATC…	No
FRMD7	c.498-3C > T	Yan N, et al. X-linked inheritances recessive of congenital nystagmus and autosomal dominant inheritances of congenital cataracts coexist in a Chinese family: a case report and literature review. BMC Medical Genetics. 2019 Dec;20:1-6.	30890130	Not tested	Congenital nystagmus	TGGCAGGAGCCCAGCTGAATCTGACATTCTGCTACTGGACATAGCAAGGAAGCTGGATATGTATGGCATCAGGCCTCACCCCGCCAGTGATGGTGAAGGGATGCAGATTCACCTGGCTGTTGCTCACATGGGAGTACTGGTGTTACGG	cagggtggaaatgtcattcttgaggcatttattaggctcataaattctttccccttt(c>t)agTGGC…	Yes
KIF5A	c.2993-3C>T	Nicolas A, et al. Genome-wide analyses identify *KIF5A* as a novel ALS gene. Neuron. 2018;97(6):1268-83.	29566793	Not tested	Amyotrophic lateral sclerosis	GAAATGCCACAGATATCAATGACAATAG	taacacccaatctccttttttcttcttctaatcctgtgttctcaatgatgatctctt(c>t)agGAAA…	Yes
LMNA	c.1158-3C>T	Wang G, et al. Somatic and germinal mosaicism in a Han Chinese family with laminopathies. Eur. J. Hum. Genet. 2022;16:1-5.	36526864	Splicing not tested. Reduced *LMNA* mRNA in proband, attributed to NMD.	Laminopathy	GCTACGCCTGTCCCCCAGCCCTACCTCGCAGCGCAGCCGTGGCCGTGCTTCCTCTCACTCATCCCAGACACAGGGTGGGGGCAGCGTCACCAAAAAGCGCAAACTGGAGTCCACTGAGAGCCGCAGCAGCTTCTCACAGCACGCACGCACTAGCGGGCGCGTGGCCGTGGAGGAGGTGGATGAGGAGGGCAAGTTTGTCCGGCTGCGCAACAAGTCCAATGAG	agtgtcctctggccggcaactggccttgactagacccccacttggtctccctctccc(c>t)agGCTA…	No
MSH2[2]	c.2635-3C>T, c.2635-5T>C	Menendez M, et al. Founder effect of a pathogenic *MSH2* mutation identified in Spanish families with Lynch syndrome. Clin. Genet. 2010;78(2):186-90.	20095990	Putative reduction in the usage of last exon or cryptic 3'ss. Reduction in mRNA expression.	Lynch syndrome	CAAGGTGAAAAAATTATTCAGGAGTTCCTGTCCAAGGTGAAACAAATGCCCTTTACTGAAATGTCAGAAGAAAACATCACAATAAAGTTAAAACAGCTAAAAGCTGAAGTAATAGCAAAGAATAATAGCTTTGTAAATGAAATCATTTCACGAATAAAAGTTACTACGTGAAAAATCCC	ctaacatgacttttagaaaagatattttaattactaatgggacattcacatgtgt(t>c)t(c>t)agCAAG…	No
NBN	c.321-3C>T, rs751356470	Yurgelun MB, et al. Cancer susceptibility gene mutations in individuals with colorectal cancer. J. Clin. Oncol. 2017;35(10):1086.	28135145	Not tested	Colorectal cancer	AATAGAGTATGAGCCTTTGGTTGCATGCTCTTCTTGTTTAGATGTCTCTGGGAAAACTGCTTTAAATCAAGCTATATTGCAACTTGGAGGATTTACTGTAAACAATTGGACAGAAGAATGCACTCACCTTGTCATGGTATCAGTGAAAGTTACCATTAAA	aaaattgccatctctgcaactctgatactatgactttatttaacttattctcattta(c>t)agAATA…	No
NIPBL	c.3856-3C>T	Rentas S, et al. Diagnosing Cornelia de Lange syndrome and related neurodevelopmental disorders using RNA sequencing. Genet. Med. 2020;22(5):927-36.	31911672	Minor exon 17 skipping by RNA-seq. Picture not convincing, no control shown	Cornelia de Lange syndrome	AATAACGATACTGAAGAAGAAGAAAGGTTATGGAGAGACCTTATTATGGAGAGAGTTACAAAATCAGCGGATGCTTGTCTTACAACTATCAACATTATGACATCCCCTAACATGCCAAAAGCTGTGTACATTGAGGATGTAATTGAAAGAGTTATACAGTACACTAAATTTCATTTGCAGAATACACTTTATCCTCAGTATGATCCTGTTTACAGATTAGATCCTCATGGAG	tgtatattttaaacctataaatgtgtttatttccatttcattaacaatactgttttta(c>t)agAATA…	Yes
PAH	c.1066-3C>T	Heintz C, et al. Splicing of phenylalanine hydroxylase (*PAH*) exon 11 is vulnerable: molecular pathology of	22698810	Exon 11 skipping /exon 11 skipping Aberrant	Phenylketonuria	TACTGCTTATCAGAGAAGCCAAAGCTTCTCCCCCTGGAGCTGGAGAAGACAGCCATCCAAAATTACACTGTCACGG	cagcagggaatactgatcctgatttaacagtgataataacttttcacttggggccta(c>t)agTACT…	No

		mutations in *PAH* exon 11. Mol. Genet. Metabol. 2012;106(4):403-11.		splicing no longer observed when four PPT guanosines were replaced with uridines.		AGTTCCAGCCCCTCTATTACGTGGCAGAGAGTTTTAATGATGCCAAGGAGAAAGTAAG		
PARN	c.178-3C>T	Kropski JA, et al. Rare genetic variants in *PARN* are associated with pulmonary fibrosis in families. Am. J. Resp. Crit. Care Med. 2017;196(11):1481-4.	28414520	cDNA tested and splicing was normal. No figure shown.	Pulmonary fibrosis	CATTCCATGGACTTTTTGCTATTTCAGTTTGGCCTTTGCACTTTTAAGTATGACTACACAGATTCAAA	gctatgctcacatggaatatgcagcacttttgtgacagatgttttatttccctttttc(c>t)agCATT…	Yes
POLR2C	c.206-3C>T	Moriwaki M, et al. *POLR2C* mutations are associated with primary ovarian insufficiency in women. J. Endocr. Soc. 2017;1(3):162-73.	29367954	Not tested	Primary ovarian insufficiency	GATTAATTCCCCTCATTAGTGATGACATTGTGGACAAGCTGCAGTACTCTCGG	gggttctcattttggcttggctgtttggttttctttaaagtgcttttctgtttttta(c>t)agGATT…	No
PRKN	c.934–3C>T	Pigullo S, et al. Mutational analysis of *parkin* gene by denaturing high-performance liquid chromatography (DHPLC) in essential tremor. Parkinsonism & Rel. Dis. 2004;10(6):357-62.	15261877	Not tested	Tremor	TACAACCGGTACCAGCAGTATGGTGCAGAGGAGTGTGTCCTGCAGATGGGGGGCGTGTTATGCCCCCGCCCTGGCTGTGGAGCGGGGCTGCTGCCGGAGCCTGACCAGAGGAAAGTCACCTGCGAAGGGGGCAATGGCCTGGGCTGTGGG	acccttggctgaaatttgcagtcagtttgaaagctcaatctgctttttgggttttg(c>t)agTACA…	No
RAD51C	c.146-3C>T	Sanoguera-Miralles L, et al. Minigene Splicing Assays Identify 20 Spliceogenic Variants of the Breast/Ovarian Cancer Susceptibility Gene *RAD51C*. Cancers. 2022;14(12):2960.	35740625	NA/100% canonical product	Hereditary cancer	AAGTTGGGATATCTAAAGCAGAAGCCTTAGAAACTCTGCAAATTATCAGAAGAGAATGTCTCACAAATAAACCAAGATATGCTGGTACATCTGAGTCACACAAGAAGTGTACAGCACTGGAACTTCTTGAGCAGGAGCATACCCAGGGCTTCATAATCACCTTCTGTTCAGCACTAGATGATATTCTTGGGGGTGGAGTGCCCTTAATGAAAACAACAGAAATTTGTGGTGCACCAGGTGTTGGAAAAACACAATTATG	atcatgttacacttttaaatctctaaaattagggttcttttttcttattttacttt(c>t)agAAGT…	No
SCN1A	c.4582-3C>T	Mahdieh N, et al. Pathogenic significance of SCN1A splicing variants causing Dravet syndrome: Improving diagnosis with targeted sequencing for variants by in silico analysis. Clin. Neurol. Neurosurg. 2018;166:80-90.	29408779	Not tested	Dravet syndrome	AACAAATTTCAAGGAATGGTCTTTGACTTCGTAACCAGACAAGTTTTTGACATAAGCATCATGATTCTCATCTGTCTTAACATGGTCACAATGATGGTGGAAACAGATGACCAGAGTGAATATGTGACTACCATTTTGTCACGCATCAATCTGGTGTTCATTGTGCTATTTACTGGAGAGTGTGTACTGAAACTCATCTCTCTACGCCATTATTATTTTACCATTGGATGGAATATTTTTGATTTTGTGGTTGTCATTCTCTCCATTGTAG	taatgctaatgcacatgagaaaactcctttgttgttaaaagcatttctatttctcta(c>t)agAACA…	No
SLC6A8	IVS12-3C>T	Rosenberg EH, et al. High prevalence of *SLC6A8* deficiency in X-linked mental retardation. Am. J. Hum. Genet. 2004;75(1):97-105.	15154114	Not tested	Mental retardation	CGCTGGCAGCACCTGACCCAGCCCATCTGGGGCCTCCACCACTTGGAGTACCGAGCTCAGGACGCAGATGTCAGGGGCCTGACCACCCTGACCCCAGTGTCCGAGAGCAGCAAGGTCGTCGTGGTGGAGAGTGTCATGTGACAA	agtcaccgtggggacgagcaggtgaccctgggggcttcagcatgtcctcctctcctg(c>t)agCGCT…	No
SMN1[3]	c.835-3C>T	Vezain M, et al. A leaky splicing mutation affecting *SMN1* exon 7 inclusion explains an unexpected mild case of spinal muscular atrophy. Hum. Mutat. 2011;32(9):989-94.	21542063	Exon 7 skipping	Spinal muscular atrophy	GGTTTCAGACAAAATCAAAAAGAAGGAAGGTGCTCACATTCCTTAAATTAAGGA	atatatagctatctatgtctatatagctattttttttaacttcctttattttcctta(c>t)agGGTTTYAG…	Yes
TGFBI[4]	IVS10-3C>T	Tsujikawa K, et al. Allelic homogeneity due to a founder mutation in Japanese patients with lattice corneal dystrophy type IIIA. Am. J. Med. Genetics. 2002;113(1):20-2.	12400061	Not tested	Corneal dystrophy	AGCCTCTGCATTGAGAACAGCTGCATCGCGGCCCACGACAAGAGGGGGAGGTACGGGACCCTGTTCACGATGGACCGGGTGCTGACCCCCCCAATGGGGACTGTCATGGATGTCCTGAAGGGAGACAATCGCTTTAG	caggaggcccctcgtggaagtataaccagtcctttctttctctgtccctcttctgtg(c>t)agAGCC…	Yes
TSC2	c.482-3C>T	Rendtorff ND, et al. Analysis of 65 tuberous sclerosis complex (TSC) patients by *TSC2* DGGE, *TSC1/TSC2* MLPA, and *TSC1* long-range PCR sequencing, and report of 28 novel mutations. Hum. Mutat. 2005;26(4):374-83.	16114042	Not tested	Tuberous sclerosis	CTGACTTTGTCCTGCAGTGGATGGATGTTGGCTTGTCCTCGGAATTCCTTCTGGTGCTGGTGAACTTGGTCAAATTCAATAGCTGTTACCTCGACGAGTACATCGCAAGGATGGTTCA	ggaggtgagtgggagatgtagattcggcgtcctcgcaaactgccgccgcttctcccc(c>t)agCTGA…	No

Legend: UTRs are underlined. [1]c.868-3T>C in the reference. [2]Founder haplotype at positions -3 and -5. [3]Haplotype -3+6 was tested in Figure 4F. [4]Allele -3C is in linkage disequilibrium with variant P501T, 93 nts downstream (…CCCC(A/C)CAATGG…). [5]c.406-3T>C in the two references.

Table S6 ClinVar records of unpublished T>C and C>T variants at position -3 relative to 3' splice site

Gene symbol	Mutation/ variant	ClinVar variant ID /URL	Possible phenotype	Exon sequence	End sequence of preceding intron (lower case)
	**-3T>C**				
ALDOA	c.542-3T>C	1977077	Aldolase A deficiency	GGTTGGATGGGCTGTCTGAGCGCTGTGCCCAGTACAAGAAGGACGGAGCTGACTTCGCCAAGTGGCGTTGTGTGCTGAAGATTGGGGAACACACCCCCTCAGCCCTCGCCATCATGGAAAATGCCAATGTTCTGGCCCGTTATGCCAGTATCTGCCAGCAG	ggagtggaaaccacatgcccctccccaccgtgctctgacccccttcctcttctct(t>c)agGGTT…
BRCA1	c.442-3T>C	409351	Breast cancer	CAGGAAACCAGTCTCAGTGTCCAACTCTCTAACCTTGGAACTGTGAGAACTCTGAGGACAAAGCAGCGGATACAACCTCAAAAGACGTCTGTCTACATTGAATTGG	aggaggaaaagcacagaactggccaacaattgcttgactgttctttaccatactgtt(t>c)agCAGG…
BRCA1	c.548-3T>C (rs397507252)	37677	Breast cancer	GATCTGATTCTTCTGAAGATACCGTTAATAAGGCAACTTATTGCAG	tttaacccttttaattaagaaaacttttattgatttattttttgggggaaattttt(t>c)agGATC…
BRCA1	c.5153-3T>C (rs375639469)	91639	Breast cancer	GGGTGACCCAGTCTATTAAAGAAAGAAAAATGCTGAATGAG	tccgtgaaaagagcacgttcttctgctgtatgtaacctgtcttttctatgatctctt(t>c)agGGGT…
NEXN	c.299-3T>C (rs397517856)	47904	Cardiomyopathy	GAACTGTGAAGGGTAGATTTGCTGAAATGGAGAAACAAAGACAAGAGGAACAAAGGAAGAGAACGGAGGAGGAACGAAAACGCAGAATTGAGCAGGATATGTTAGAAAAGAGGAAAATACAGCGTGAATTAGCAAAAAGGGCTGAACAG	aattaaattgcaaaatagaaacataaccaagtatcaaacttttttttcatatatttt(t>c)agGAAA…
PLEKHG 5	c.3012-3T>C	1798815	Inborn genetic diseases screening	GGAGGTCTGAGCAGAG ...	aagagagggtgaccagagccactctgaatggcgtcagccaaagaaggactctgtcccctccccaccctg(t>c)agGGAGGTCTGAGCAGAG…
RYR2	c.9450-3T>C (rs188671846)	43838	Cardiomyopathy	GCAACGTTCTGCATTAGGAGAATGTCTAGCTGCCTTTGCTGGTGCTTTTCCTGTAGCATTTTTGGAAACTCATCTGGACAAACATAATATTTACTCCATCTACAATACCAAGTCTTCACGAGAAAGAGCAG	tatgactttttagttactcacttggaagaccttaaaacataagcattttccactta(t>c)agGCAA
	**-3C>T**				
AIFM1	c.1574-3C>T	2124664	Charcot-Marie-Tooth neuropathy	GAACTGGTATCCGATCAGAGAGTGAGACAGAGTCCGAGGCCTCAGAAATTACTATTCCTCCCAGCACCCCGGCAGTTCCACAGGCTCCCGTCCAGGGGGAGGACTACGGCAAAGGTGTCATCTTCTACCTCAGGGACAAAGTGGTCGTGGGGATTGTGCTATGGAACATCTTTAACCGAATGCCAATAGCAAGGAAG	cccaagctcataataactgaagttttgattttgcttccatgtttatttgttttgggggctggcca(c>t)agGAAC…
ALG8	c.1350-3C>T	2267117	Inborn genetic diseases screening	AAAAGAAAAACCTCTTTTTAATTGGATGGAAACTTTCTACCTGCTTGGCCTGGGGCCTCTGGAAGTCTGCTGTGAATTTGTATTCCCTTTCACCTCCTGGAAGGTGAAGTACCCCTTCATCCCTTTGTTACTAACCTCAGTGTATTGTGCAGTAGGCATCACATATGCTTGGTTCAAACTGTATGTTTCAGTATTGATTGACTCTGCTATTGGCAAGACAAAGAAACAATGATATGTG...	agatggggaagctttagctaggaaatgtttgctaaaccaggatgactcatctaaaatcagacttttctctctttttttcctt(c>t)agAAAA…
APC	c.730-3C>T (rs786203125)	186663	Familial adenomatous polyposis coli	AGGTCATCTCAGAACAAGCATGAAACCGGCTCACATGATGCTGAGCGGCAGAATGAAGGTCAAGGAGTGGGAGAAATCAACATGGCAACTTCTGGTAATGGTCAG	taagaaagcctacaccatttttgcatgtactgatgttaactccatcttaa(c>t)agAGGT…
ATP13A2	c.289-3C>T	1339099	Spastic paraplegia	GATAGTTCCTGGCAGCTCTTCACTGTCCAGGTGCAGACTGAGGCCATCGGCGAGGGCAG	gacagggcctgggaggggaggggactcttctgagactgagtccatacctctgtgttc(c>t)agGATA…
ATM	c.6348-3C>T	453623	Hereditary cancer	CAAAGAAGTAGAAGGAACCAGTTACCATGAATCATTGTACAATGCTCTACAATCTCTAAGAGACAGAGAATTCTCTACATTTTATGAAAGTCTCAAATATGCCAG	tacatgtatatcttagggttctgtttttaagtatattttttctttgacttatctca(c>t)agCAAA…
BRCA1	c.5075-3C>T (rs398122690)	384512	Breast-ovarian cancer	ATGCTGAGTTTGTGTGTGAACGGACACTGAAATATTTTCTAGGAATTGCGGGAGGAAAATGGGTAGTTAGCTATTTCT	cacttcctgattttgttttcaacttctaatcctttgagtgtttttcattctg(c>t)agATGC…
BRCA2	c.476-3C>T (rs371431745)	37925	Hereditary breast/ovarian cancer	TGGTATGTGGGAGTTTGTTTCATACACCAAAGTTTGTGAAG	gaatactagaaatgttaataaaaataaaacttaacaattttccccttttttttacccc(c>t)agTGGT…
BRCA2	c.8954-3C>T (rs81002844)	822839	Hereditary breast ovarian cancer syndrome	TTATACTGAGTATTTGGCGTCCATCATCAGATTTATATTCTCTGTTAACAGAAGGAAAGAGATACAGAATTTATCATCTTGCAACTTCAAAATCTAAAAGTAAATCTGAAAGAGCTAACATACAGTTAGCAGCGACAAAAAAAAACTCAGTATCAACAACTACCG	acaaacatttaaatgataatcacttcttccattgcatctttctcatctttctccaaa(c>t)agTTAT…
CASP10	c.923-3C>T	333434	Autoimmune lymphoproliferative syndrome	AGATCCTGAGTCATGTGTTCCAGTGGCTTGGGTTCACAGTGCATATACACAATAATGTGACGAAAGTGGAAATGGAGATGGTCCTGCAGAAGCAGAAGTGCAATCCAGCCCATGCCGACGGGGACTGCTTCGTGTTCTGTATTCTGACCCATGGGAGATTTGGAGCTGTCTACTCTTCGGATGAGGCCCTCATTCCCATTCGGGAGATCATGTCTCACTTCACAGCCCTGCAGTGCCCTAGACTGGCTGAAAAACCTAAACTCTTTTTCATCCAGGCCTGCCAAGGTGAAGAGATACAGCCTTCCGTATCCATCGAAGCAGATGCTCTGAACCCTGAGCAGGCACCCACTTCCCTGCAGGACAGTATTCCTGCCGAGGCTGACTTCCTACTTGGTCTGGCCACTGTCCCAGGCTATGTATCCTTTCGGCATGTGGAGGAAGGCAGCTGGTATATTCAGTCTCTGTGTAATCATCTGAAGAAATTGGTCCCAAG	tttattatttccccttctctctctctctctcttttttttttttttgtttttaaa(c>t)agAGAT…
CDKN2A	c.458-3C>T	1741656	Hereditary cancer-predisposing syndrome	ACATCCCCGATTGAAAGAAC	gcaagagaggagggcgggatgtgccacacatctttgacctcaggtttctaacgcctgttttctttctgccctctg(c>t)agACATCCCCGATTGAAAGAAC

					…
CDH1	c.833-3C>T	142944	Hereditary cancer-predisposing syndrome	GAACCTCTGTGATGGAGGTCACAGCCACAGACGCGGACGATGATGTGAACACCTACAATGCCGCCATCGCTTACACCATCCTCAGCCAAGATCCTGAGCTCCCTGACAAAAATATGTTCACCATTAACAGGAACACAGGAGTCATCAGTGTGGTCACCACTGGGCTGGACCGAGAG	gtattgacccagtcccaaagtgcagcttgtctaaaccttcatctccttgaactcttc(c>t)agGAAC…
COL11A2	c.3583-3C>T (rs727502939)	162982	Not specified	GGCCCACAAGGTCCCCCAGGAGGTGTTGGGAACCTGGGTCCCCCTGGAGAGAAG	ttgcccctgaaatggcacctccatcctaagcaatgacacctatttctgttcctcttc(c>t)ag
C1QTNF5	c.215-3C>T	1517901	Not specified	GACTGCCGGGACCTCGAGGGGACCCCGGGCCGCGAGGAGAGGCGGGACCCGCGGGGCCCACCGGGCCTGCCGGGGAGTGCTCGGTGCCTCCGCGATCCGCCTTCAGCGCCAAGCGCTCCGAGAGCCGGGTGCCTCCGCCGTCTGACGCACCCTTGCCCTTCGACCGCGTGCTGGTGAACGAGCAGGGACATTACGACGCCGTCACCGGCAAGTTCACCTGCCAGGTGCCTGGGGTCTACTACTTCGCCGTCCATGCCACCGTCTACCGGGCCAGCCTGCAGTTTGATCTGGTGAAGAATGGCGAATCCATTGCCTCTTTCTTCCAGTTTTTCGGGGGGTGGCCCAAGCCAGCCTCGCTCTCGGGGGGGGCCATGGTGAGGCTGGAGCCTGAGGACCAAGTGTGGGTGCAGGTGGGTGTGGGTGACTACATTGGCATCTATGCCAGCATCAAGACAGACAGCACCTTCTCCGGATTTCTGGTGTACTCCGACTGGCACAGCTCCCCAGTCTTTGCTTAGTGCCCACTGCAAAGTGAGCTCATGCTCTC…	gcaagagaggagggcgggatgtgccacacatctttgacctcaggtttctaacgcctgttttctttctgccctctg(c>t)agGACT…
DMD	c.9362-3C>T (rs752816247)	228596	Muscular dystrophy	TGGATCTCTTGAGCCTGTCAGCTGCATGTGATGCCTTGGACCAGCACAACCTCAAGCAAAATGACCAGCCCATGGATATCCTGCAGATTATTAATTGTTTGACCACTATTTATGACCGCCTGGAGCAAGAGCACAACAATTTGGTCAACGTCCCTCTCTGCGTGGATATGTGTCTGAACTGGCTGCTGAATGTTTATGATAC	ggacactgaaaggaaggttttactctttgagtcatttgtgattttatttgtttttg(c>t)agTGGA…
EYA1	c.1699-3C>T	48105	Otofaciocervical syndrome	CACGCGATGCCCTTCTGGAGGATCTCCAGCCACTCGGACCTCATGGCCCTGCACCATGCCTTGGAACTGGAGTACCTGTAACAGCG…	aagctggcagggctggcgctgggtgctctgtcctcacatgtatgtgtgcctctgctg(c>t)agCACG…
HRAS	c.451-3C>T (rs1158205210)	648589	Costello syndrome	CACAAGCTCAGGACATGGA...	TGACGCAGgtgagggggactcccagggcggccgccacgcccaccggatgaccccggctccccgcccctgccggtctcctggcctgcggtcagcagcctcccttgtgccccgcc(c>t)agCACAAGCTCAGGACATGGA...
LITAF	c.378-3C>T (rs774213197)	571959	Charcot-Marie-Tooth disease	GTGCATAGCGGGCTGCTGCTTCATCCCCTTCTGCGTGGATGCCCTGCAGGACGTGGACCATTACTGTCCCAACTGCAGAGCTCTCCTGGGCACCTACAAGCGTTTGTAGGACTCAGCCAGACG…	agccagtgttcaccctgttgtgatcagtaacgcgctccgtgtgtctctctctcctcc(c>t)agGTGC…
MKS1	c.1589-3C>T (rs752901746)	891162	Meckel syndrome	AGGCCTTCCGTCGAGCCCGGCGCCGCATGCAGGAGGCCCGGGAAAGCCTCCCGCAGGACCTAGTGAGCCCCTCTGGAACCCTGGTCTCCTAGCTCACA...	gtatgtcccctcccccagctggccaccccaggtcagcatgggcctgtttctctctgcagtgcagagagaaataccatatggcagccaaatagcatatctcctttcctta(c>t)agAGGC…
MLH1	c.1897-3C>T (rs748763466)	234627	Colorectal cancer	GAAGGGAACCTGATTGGATTACCCCTTCTGATTGACAACTATGTGCCCCCTTTGGAGGGACTGCCTATCTTCATTCTTCGACTAGCCACTGAG	cacccagagtggcagataggagcacaaggcctgggaaagcactggagaaatgggatttgt ttaaactatgacagcattatttcttgttcccttgtccttttttcctgcaag(c>t)agGAAG…
MSH2	c.1077-3C>T (rs758182607)	232625	Colorectal cancer	ATTGAATTTAGTGGAAGCTTTTGTAGAAGATGCAGAATTGAGGCAGACTTTACAAGAAGATTTACTTCGTCGATTCCCAGATCTTAACCGACTTGCCAAGAAGTTTCAAAGACAAGCAGCAAACTTACAAGATTGTTACCGACTCTATCAGGGTATAAATCAACTACCTAATGTTATACAGGCTCTGGAAAAACATGAAG	ttgataaattttaattttatactaaaatatttttacattaattcaagttaatttattt(c>t)agATTG…
MYH14	c.3468-3C>T	44066	Autosomal dominant nonsyndromic hearing loss	GGCAGAAGACGAGGGTGGGGCCCGGGCCCAGCTGCTGAAATCCCTGCGGGAGGCTCAAGCAGCCCTGGCCGAGGCCCAGGAGGACCTGGAGTCTGAGCGTGTGGCCAGGACCAAGGCGGAGAAGCAGCGCCGGGACCTGGGCGAGGAGCTGGAGGCGCTGCGGGGCGAGCTGGAGGACACGCTGGACTCCACCAACGCACAGCAGGAGCTCCG	aaatcattgcctcactctggcctgcccctgtatcaactccacggttcttgtcacccc(c>t)agGGCA…
MYO15A	c.10217-3C>T (rs727503321)	164574	Not specified	GCCTCCTCAGCGCCTTACCTATGTTCGGCTCCTCCTTCTTCTTCATCCAGAGCTGCAGCAACATTGCTGTGCCAGCCCCTTGCATCCTTGCCATCAACCACAATGGCCTCAACTTTCTCAGCACAGAGACTCAT	catggctgttgtcagtgagccctgcccagcacgtaactgccacccccctctccctgcc(c>t)agGCCT…
NEBL	c.1963-3C>T (rs727504683)	179169	Not specified	CTCCAGTATAAAGAGCAAAACTACAAGGCCACTCCGGTAAGCATGACCCCGGAGATAGAGAGAGTGAGGCGAAACCAGGAGCAGCTGAGTGCG	aatgttttgggacgtatttatttcccataatctcaaactgtcttgcaccttgtctct(c>t)agCTCC...
PCDH15	c.475-3C>T	46485	Usher syndrome	CTCACTCCAGTTGGTACCACAATATTCACAGGATTTTCAGGAGACAATGGAGCTACAGATATAGATGATGGACCAAATGGACAGATAGAGTATGTTATTCAGTATAATCCAGATGATCCG	catttctggtgtgcagttgataaaatgaaaattgatatttaaaatcttttttccttt(c>t)agCTCA…
RAD51C	c.966-3C>T (rs1064796091)	422917	Hereditary breast/ovarian cancer-	GTTGGCAACATTGTACAAGTCACCCAGCCAGAAGGAATGCACAGTACTGTTTCAAATCAAA	tgaacttttaattaattaagttcatgtgtttgtatgtatttattctttttctttaag(c>t)agGTTG...
RYR1	c.7215-3C>T	1339042	Myopathy	CTTTGGTGAGGAACCGCCTGAAGAAAACCGGGTGCACCTGGGACACGCCATCATGTCCTTCTATGCCGCCTTGATCGACCTGCTCGGACGCTGTGCACCAGAGATGCAT	tgagtctcccggccccctcctcaatagggcaacccgccctccctggcccctggctgcctccccaacccacccaccttccctg(c>t)agCTTT...
SPINK2	c.360-3C>T (rs375854132)	1028720	Spermatogenic failure	GGAAGGTGGTCATAATATTAAAATCATTCGAAATGGACCCTGCTGATGGAGC...	aatgagacagtttcagtatggtaggtaataaatgtatttctatgattgttcaatttgcagGGAA...

TP53	c.97-3C>T (rs786203749)	187457	Li-Fraumeni syndrome	TCCCCCTTGCCGTCCCAAGCAATGGATGATTTGATGCTGTCCCCGGACGATATTGAACAATGGTTCACTGAAGACCCAGGTCCAGATGAAGCTCCCAGAATGCCAGAGGCTGCTCCCCCCGTGGCCCCTGCACCAGCAGCTCCTACACCGGCGGCCCCTGCACCAGCCCCCTCCTGGCCCCTGTCATCTTCTGTCCCTTCCCAGAAAACCTACCAGGGCAGCTACGGTTTCCGTCTGGGCTTCTTGCATTCTGGGACAGCCAAGTCTGTGACTTGCACG	taaggacaagggttgggctggggacctggagggctggggacctggagggctgggggctggggggctgaggacctggtcctctgactgctcttttcacccatcta(c>t)agTCCC...
TP53	c.1101-3C>T (rs1057521167)	381768	Li-Fraumeni syndrome	CCACCTGAAGTCCAAAAAGGGTCAGTCTACCTCCCGCCATAAAAAACTCATGTTCAAGACAGAAGGGCCTGACTCAGACTGA CATCCT...	gcattggtcagggaaaaggggcacagaccctctcactcatgtgatgtcatctctcctccctgcttctgtctccta(c>t)agCCAC…
TSC2	c.3815-3C>T (rs770054036)	406073	Tuberous sclerosis	TGGCCTCTTTCTCCTCCCTGTACCAGTCCAGCTGCCAAGGACAGCTGCACAGGAGCGTTTCCTGGGCAG	ctcgacctgtgtgtagcccctcctcctgctgacgtggccgcacacggccttcccttg(c>t)agTGGC…
TTN	c.1399-3C>T	46630	Myopathy	GTAAGAAAGGAAGCGGAGAAGACTGCTGTAACTAAGGTAGTAGTGGCCGCCGATAAAGCCAAGGAACAAGAATTAAAATCAAGAACCAAAGAAGTAATTACCACAAAGCAAGAGCAGATGCACGTAACTCATGAGCAG	gttggcgaggctggtcttgaactaattttatgtaagatgcattaaggtgttttcttcctttctctatgaaaag(c>t)agGTAA…
VHL	c.464-3C>T (rs904414377)	644549	Von Hippel-Lindau syndrome	TGTATACTCTGAAAGAGCGATGCCTCCAGGTTGTCCGGAGCCTAGTCAAGCCTGAGAATTACAGGAGACTGGACATCGTCAGGTCGCTCTACGAAGATCTGGAAGACCACCCAAATGTGCAGAAAGACCTGGAGCGGCTGACACAGGAGCGCATTGCACATCAACGGATGGGAGATTGAAGATTT...	tagtacaggtagttgttggcaaagcctcttgttcgttccttgtactgagaccctagtctgccactgaggatttggtttttgcccttc(c>t)agTGTA…

Table S7 *ln*(ESEf/ESSf) values weighted for codon usage frequencies show reduced or absent correlation with multiple predictors of intrinsic disorder

***ln*(ESEf/ESSf)**	**Top-IDP**	**B-value**	**FoldUnfold**	**DisProt**
R	0.45	0.50	-0.56	0.55
P value	0.05	0.02	0.01	0.011
**M phase**				
R	0.38	0.44	-0.50	0.5
P value	0.10	0.05	0.026	0.02
**Pattern specification**				
R	0.23	0.37	-0.36	0.31
P value	0.34	0.11	0.12	0.18

Codon usage frequencies in genes involved in cell division (M phase) and cell differentiation (pattern specification) were taken from Gingold et al. (13). IDR predictors were defined previously (12).

Table S8 Candidate *trans*-acting factors for differential recognition of Zn^{2+} and Ca^{2+} exons

**Metal coordinating amino acid**	**Codon[1]**	**RNA-binding proteins that bind codon-containing consensus motifs[2]**
C	UGU	KHSRP, FUBP3
	UGC	*CG7903*
H	CAU	RBFOX2
	CAC	HNRNPL, IGF2BP2
E	GAA	TRA2A
	GAG	EWSR1, TAF15, SRSF9, *RNP4F*
D	GAU	*CG14718, HNRNPAB*
	GAC	*ANKHD1, FMR1, FXR1, FXR2, RSF1, RBM45*

Legend: [1], Preferred codons are in bold. [2] Candidate RBPs were compiled from Ray et al. (14) (*italicized*) and Van Nostrand et al. (15).

Table S9 Predicted Zn^{2+} binding to structured metalloproteins and intrinsically disordered metal-binding proteins[1]

	Structured regions	Unstructured regions (IDRs)
**Metal binding site**	Usually one and well-defined for each metal	More sites, poorly defined
**Mutations changing location of metal binding sites**	Rare	Common
**Kinetics**	Slow, often trapped	Fast (<ms for intra- and inter-peptide metal transfers)
**Binding affinity**	High	Modest
**Reactivity**	High	Low
**Selectivity**	High	Low
**Subcellular localization**	Does not strictly require distinct compartments	Metal binding probably requires compartments or conditions with a loosely bound metal

[1]Adapted from Faller et al. (16).

REFERENCES TO SUPPLEMENTARY INFORMATION

1. Sievers, F., Wilm, A., Dineen, D., Gibson, T.J., Karplus, K., Li, W., Lopez, R., McWilliam, H., Remmert, M., Soding, J. *et al.* (2011) Fast, scalable generation of high-quality multiple sequence alignments using Clustal Omega. *Mol. Syst. Biol.*, **7**, 539.
2. Chan, A.C.K., Blair, K.M., Liu, Y., Frirdich, E., Gaynor, E.C., Tanner, M.E., Salama, N.R. and Murphy, M.E.P. (2015) Helical shape of *Helicobacter pylori* requires an atypical glutamine as a zinc ligand in the carboxypeptidase Csd4. *J. Biol. Chem.*, **290**, 3622–3638.
3. Ferraroni, M., Tilli, S., Briganti, F., Chegwidden, W.R., Supuran, C.T., Wiebauer, K.E., Tashian, R.E. and Scozzafava, A. (2002) Crystal structure of a zinc-activated variant of human carbonic anhydrase I, CA I Michigan 1: evidence for a second zinc binding site involving arginine coordination. *Biochemistry.* **41**, 6237-6244.
4. Katz, B.A., Clark, J.M., Finer-Moore, J.S., Jenkins, T.E., Johnson, C.R., Ross, M.J., Luong, C., Moore, W.R. and Stroud, R.M. (1998) Design of potent selective zinc-mediated serine protease inhibitors. *Nature*, **391**, 608-612.
5. Ohana, E., Hoch, E., Keasar, C., Kambe, T., Yifrach, O., Hershfinkel, M. and Sekler, I. (2009) Identification of the Zn2+ binding site and mode of operation of a mammalian Zn2+ transporter. *J. Biol. Chem.*, **284**, 17677–17686.
6. Xue, J., Xie, T., Zeng, W., Jiang, Y. and Bai, X.-C. (2020) Cryo-EM structures of human ZnT8 in both outward- and inward-facing conformations. *eLife*, **9**, 58823.
7. Lu, M. and Fu, D. (2007) Structure of zinc transporter YiiP. *Science*, **317**, 1746-1748.
8. Tapial, J., Ha, K.C., Sterne-Weiler, T., Gohr, A., Braunschweig, U., Hermoso-Pulido, A., Quesnel-Vallieres, M., Permanyer, J., Sodaei, R., Marquez, Y. *et al.* (2017) An atlas of alternative splicing profiles and functional associations reveals new regulatory programs and genes that simultaneously express multiple major isoforms. *Genome Res.*, **27**, 1759-1768.
9. Busch, A. and Hertel, K.J. (2013) HEXEvent: a database of Human EXon splicing Events. *Nucleic Acids Res.*, **41**, D118-D124.
10. Corvelo, A., Hallegger, M., Smith, C.W. and Eyras, E. (2010) Genome-wide association between branch point properties and alternative splicing. *PLoS Comput. Biol.*, **6**, e1001016.
11. Quaglia, F.e.a. (2022) DisProt in 2022: improved quality and accessibility of protein intrinsic disorder annotation. *Nucleic Acids Res.*, **50**, D480–D487.
12. Campen, A., Williams, R.M., Brown, C.J., Meng, J., Uversky, V.N. and Dunker, A.K. (2008) TOP-IDP-scale: a new amino acid scale measuring propensity for intrinsic disorder. *Protein Pept. Lett.*, **15**, 956–963.
13. Gingold, H., Tehler, D., Christoffersen, N.R., Nielsen, M.M., Asmar, F., Kooistra, S.M., Christophersen, N.S., Christensen, L.L., Borre, M., Sorensen, K.D. *et al.* (2014) A dual program for translation regulation in cellular proliferation and differentiation. *Cell*, **158**, 1281–1292.
14. Ray, D., Kazan, H., Cook, K.B., Weirauch, M.T., Najafabadi, H.S., Li, X., Gueroussov, S., Albu, M., Zheng, H., Yang, A. *et al.* (2013) A compendium of RNA-binding motifs for decoding gene regulation. *Nature*, **499**, 172-177.
15. Van Nostrand, E.L., Freeze, P., Pratt, G.A., Wang, X., Wei, X., Xiao, R., Blue, S.M., Chen, J.-Y., Cody, N.A., Dominguez, D. *et al.* (2020) A large-scale binding and functional map of human RNA-binding proteins. *Nature*, **583**, 711-719.
16. Faller, P., Hureau, C. and La Penna, G. (2014) Metal ions and intrinsically disordered proteins and peptides: from Cu/Zn amyloid-beta to general principles. *Acc. Chem. Res.*, **47**, 2252-2259.
